# Organic transformations in the confined space of porous organic cage CC2; catalysis or inhibition†

**DOI:** 10.1039/d2ra03399b

**Published:** 2022-08-26

**Authors:** Ayesha Mukhtar, Sehrish Sarfaraz, Khurshid Ayub

**Affiliations:** Department of Chemistry, COMSATS University Abbottabad Campus KPK Pakistan 22060 khurshid@cuiatd.edu.pk +92-992-383591

## Abstract

Porous organic cages have shape persistent cavities which provide a suitable platform for encapsulation of guest molecules with size suitably fitting to the cavity. The interactions of the guest molecule with the porous organic cage significantly alter the properties of the guest molecule. Herein, we report the effect of encapsulation on the kinetics of various organic transformations including 2 + 4 cycloaddition, 1,5-sigmatropic, 6π-electrocyclization, ring expansion, cheletropic, dyotropic, trimerization and tautomerization reactions. Non-bonding interactions are generated between the CC2 cage and encapsulated species. However, the number and nature/strength of interactions are different for reactant and TS with the CC2 cage and this difference detects the reaction to be accelerated or slowed down. A significant drop in the barrier of reactions is observed for reactions involving strong interactions of the transition state within the cage. However, for some reactions such as the Claisen rearrangement, reactants are stabilized more than the transition state and therefore an increase in activation barrier is observed. Furthermore, non-covalent analyses of all transition states (inside the cage) confirm the interaction between the CC2 cage and substrate. The current study will promote further exploration of the potential of other porous structures for similar applications.

## Abstract 2

Porous organic cages have shape persistent cavities which provide a suitable platform for encapsulation of guest molecules with size suitably fitting to the cavity.

## Graphical abstract

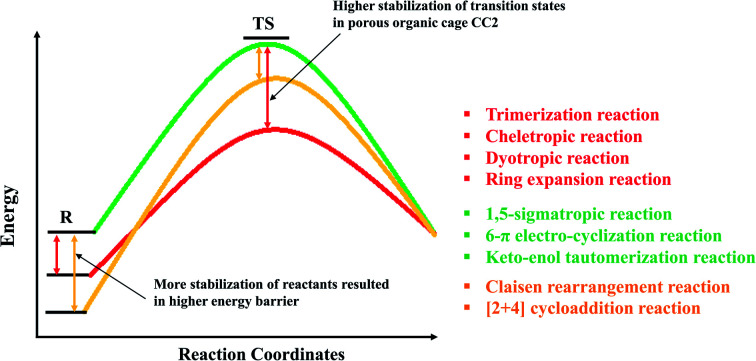

## 1 Introduction

A rapid increase in the applications of porous materials in various applications has been witnessed recently because of their unique structural features such as porosity, persistent shapes and large surface area. Metal organic frameworks (MOFs) are typical examples of porous materials which find application in the field of catalysis,^1,2^ adsorption,^3,4^ photocatalysis^5^ separation and purification. Besides MOFs, zeolites are also porous materials with many similar applications. In 2009, Karapınar, N. reported some natural zeolites as a good choice for the removal^6^ of phosphorus and ammonium through aqueous solution. Later on Mingyu Li *et al.*^7^ published work on the removal of ammonia from edible or drinking water through modified zeolites. Catalysis,^8^ food production, agriculture,^9^ adsorption^10^ and separation^11,12^ are the most reported applications of zeolites.

Over the last few decades, several porous materials have been synthesized including covalent organic frameworks. Porous organic cages (POCs)^13^ are one of the commodious and latest class of porous compounds with single and multiple cavities. Shape persistency and covalently bonded framework^14^ of porous organic cages make suitable substrate for guest molecules. POCs bear more than one opening door through which analytes can enter to the cavity. Sensing ability of certain porous compounds depends upon their structural skeleton. Porous organic cages (POCs) are one of highly potent affinity^15^ compounds which are used as a sensing object in quartz crystal microbalances (QCMs). For the adsorption of sulfur hexafluoride (SF6),^16^ porous organic cages are reported as superior platform than metal organic framework and fullerene. Similarly, Chen *et al.* successfully separated the rare gases and chiral compounds^17^ with the help of porous organic cages, which is also a challenging task ever. In 2015, CC10 was reported for separation of enantiomers.^18^

The porous organic cages are also reported for the adsorption of radioactive species^19^ such as strontium and cesium ions. These confinements have greater effects in separation, adsorptions catalysis and kinetic controls.^20^ Selectivity has been shown by the cage system in some common industrial gases such as H_2_/N_2_,^21^ CO_2_/CH_4_,^22^ SF_6_/N_2_,^23^ CO_2_/N_2_.^24,25^ Large sized porous organic cages have been investigated for the separation of large organic molecules such as organic molecules and noble gases. Recently, In 2019, another chiral porous organic cage CC3-R was introduced with potentiometric sensing properties^26^ of chiral alcohol amines (*S*-2-amino-1-butanol and *R*-2-amino-1-butanol).

POCs have been reported multiple times for their application in gas chromatography columns. The capillary columns^27^ coated with molecular cage have shown very good selectivity for chiral resolution and separation of a number of structural isomers, such as aromatic hydrocarbons, *n*-alcohols and *n*-alkanes. Specific adsorption properties can be incorporated into porous compounds, such as, water tolerance. Stable non-covalent organic frameworks which are based on electron rich pyrazoles have shown very good adsorption properties for ozone-depleting substances (fluorocarbons and CFCs) and hydrocarbons.^28^ Some porous organic cages with high electron delocalization in their π-conjugated system along with rigid structure are fluorescent and these compounds have been used for chiral^29^ fluorescent sensing. In 2018 Zhenyu Lu and his co-workers have designed a new modified chiral porous organic cage with sensing abilities. The CD@RCC3 POC for 349 fluorescent sensing possesses a stable and strong fluorescent property. CD@RCC3 has been reported as fluorescence chemosensor^30^ for the sensing of phenyl alaninol, phenyl ethanol enantiomers and nitrophenol isomers.

Covalently bonded crystal structure of porous organic cages makes them feasible for catalysis applications. Cage immobilized catalyst have the potential to solubilize the heterogeneous catalytic particles. The metal nanoparticles confinement in the cavity of cage also raised the possibilities of size selective catalysis by the control of guest accessing through the window of cage. CCR-3 cage has been used to support rhodium (Rh) nanoparticles with reasonable size.^31^ Isolation of nanoparticles also reduced the aggregation between nanoparticles. The obtained Rh/CC3-R-homo (homogenized heterogeneous catalyst) exhibits very significant catalytic performance towards different liquid phase catalytic reactions (*e.g.* methanolysis of ammonia borane), while Rh/CC3-R-hetero^32^ did not show such efficiency. The recyclability and durability of Rh/CC3-R-homo is also excellent. Porous organic cage with well-dispersed embedded nanoparticles (NPs)^33^ have efficient catalytic application in cyanation, of aryl halide under additive free and heterogeneous conditions.^34^ Porphyrin-based POCs has been reported by Kim and Chang for electrochemical reduction of CO_2_.^35,36^ The encapsulation of metal nanoclusters with high catalytic activity inside POCs is elucidated by Xu *et al.*^37^ POCs (CC3-R cage) are reported to exhibit remarkably improved catalytic activities towards the selective hydrolysis of ammonia borane. Dong *et al.* further reported that CC3-R cage exhibits higher selectivity and conversion under normal conditions for the tandem hydrogenation of quinoline and nitroarenes in water.^38–40^ Recently, Wang *et al.* reported triphenylphosphine-based quasi-POCs as remarkable organic cage system to catalyze hydroformylation reactions with more than 97% aldehyde selectivity.^41^ Furthermore, the performance of Pd doped RCC3 cage toward semi-hydrogenation of alkyne and hydrogenation of 4-nitrophenol compounds was demonstrated both experimentally and theoretically by Kou *et al.*^42^ Covalent organic cages (COCs) are also reported with high catalytic efficiency for hydrogen evolution reaction (HER).^43^

Literature reveals several examples where confinement of reagents in certain cavities has remarkable effect on the activation barriers. Catalytic activities of self-assembled metallocage for Diels–Alder reaction were investigated through encapsulation of Diels–Alder reactants inside the cage.^44^ In 2002 Halls *et al.* theoretically investigated activation energy and enthalpies for Menshutkin SN2 reaction^45^ inside CNTs and achieved a significant reduction in the reaction barrier. Decomposition of chloromethanol and dichloromethanol^46^ was carried out inside the carbon nanotube of different diameters, where the CNT with smaller diameter resulted in lowest activation barrier. Thermodynamic and kinetic study of Diels Alder reactant inside CNTs^47^ was investigated theoretically; whereas minimum energy of activation was obtained in case of SWCNT. Recently Inês Alves and his co-worker^48^ reported the DFT studies on SN_2_ reaction inside the different single walled carbon nanotubes (SWCNTs) for their kinetic barrier. Among the selected SWCNT, BN doped SWCNT lowered the energy barrier for SN2 reaction. Up till now, successful alteration in activation barriers for organic transformations is achieved only with carbon nanotubes. It has been shown previously by Alves *et al.* that BN doping in SWCNT creates electrophilic and nucleophilic sites which significantly affect the energy barriers by interaction with the substrates either in the reactants or transition states. Moreover, these nanotubes have quite wide cavities where the reagents may not feel the proper influence of encapsulations. Another drawback associated with doped SWCNT is the selective placement of dopant because of associated challenges, and to have reproducible results. We, instead of creating electrophilic and nucleophilic sites through doping, were more interested in ordered predefined orientation of electrophilic and nucleophilic sites in porous organic cages where structure is compact and reproducible. Moreover, these cages possess nitrogen atoms which can act as hydrogen bond acceptor in the close proximity of reacting atoms. For this purpose, we have chosen CC2 cage because it contains not only the nitrogen heteroatom (for hydrogen bond type of interaction with the substrate or transition state), but it also has compact structure where the cavity inside is relatively small. This small cavity restricts freedom of reagents inside the cage and force the substrate or transition state to form some non-bonding interaction. Our objective was to study the influence of these interaction on the activation barriers for several organic transformations.

Therefore, in current study, we are interested in studying chemical reactions inside porous organic cage confinement which imparts significant effect on the reactivity. Porous organic CC2 cages exhibit high surface area, appropriate chemical composition, high rigidity, and persistency. The above-mentioned characteristics make CC2 cage an excellent cage to carry out organic transformations within its confined spaces. Therefore, we have selected this class of porous materials (CC2 cage) to investigate some simple chemical reaction for their kinetic transformations within the cage and without cage. We performed DFT study on nine different organic transformations including: 2 + 4 cycloaddition, 1,5-sigmatropic, 6π-electro-cyclization, ring expansion, cheletropic, dyotropic, trimerization and tautomerization reactions. Furthermore, we explored the positive as well as negative catalytic influence of CC2 cage on the studied organic chemical reactions. Since these organic transformations are quite diverse therefore, it is not expected that a similar type of effect may be seen in all of them. Therefore, some organic transformations are catalyzed whereas others are slowed down (increase of barrier).

## 2 Methodology

Gaussian 09 (ref. 49) was used to perform all calculations whereas Gauss-View 5.0 is used for visualization. Optimization of geometries was performed at M06-2X/6-31G(d)^50^ level of theory. [2 + 4] cycloaddition,^51^ 6π-electrocyclization,^52^ dyotropic reaction,^53^ cheletropic,^54^ 1,5-sigmatropic reaction,^55^ Claisen rearrangement,^56^ ring expansion and contraction reaction^57^ keto-enol tautomerization reaction^58^ and trimerization reaction^59^ have been selected to investigate their energies (activation energy and energy of reaction) without CC2 cage and inside the CC2 cage. All optimized structures were confirmed by means of frequency analysis. Transition states have been optimized through Berny algorithm. Whereas stationary points were characterized as minima (no imaginary frequency) or transition state (one imaginary frequency). Furthermore, imaginary frequencies of all transition states were evaluated to confirm that their associated eigenvectors correspond to the motion along the reaction coordinates. Obtained results (activation energy and geometric parameters) of reactions within the cage and without cage were compared. All calculated energies are in kcal mol^−1^ while bond lengths for all optimized structures are reported in angstrom (Å). Non-covalent interactions are studied through NCI analysis. Multiwfn and VMD software have been used for the analysis of non-covalent interactions inside the CC2 cage. Results of non-covalent interactions have been reported in the form of 3D isosurface images and 2D reduced density graphs.

## 3 Result and discussion

Porous organic cages represent novel porous compounds with permanent and shape persistent cavities. Shape persistent [4 + 6] imine cages are generally represented by CC1, CC2, CC3 *etc.* They are different from each other based on vicinal diamine. The cage of our interest is CC2 with tetrahedral geometry with four triangular windows which form window-arene stacks while methyl groups are attached at vicinal diamine. The diameter of pore limiting window for CC2 cage is 3.6 Å at 298 K under 1 bar pressure^45^ and BET surface area is 533 m^2^ g^−1^.

**Fig. 1:**
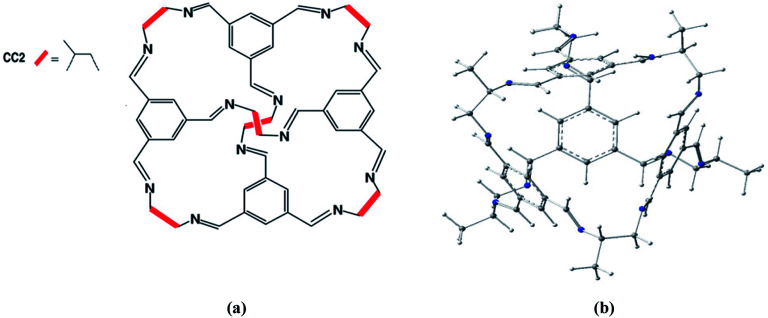
(a) Chemical structure of structure of CC2 porous organic cage. (b) Optimized 3D structure of CC2 porous organic cage at M06-2X/6-31G(d).

CC2 porous organic cage was optimized at M06-2X/6-31G(d) level of theory to get the minimum at PES (Fig. 1). According to Mulliken charges, nitrogen is the most electronegative and electron rich specie of the cage. Carbon atoms with π-electrons have partial negative charge while hydrogen atoms bear positive charges. Electronic density spreads all over the porous organic cage which provides best platform for atoms of guest molecules to interact on both internal and external surfaces. Intrinsically the effect of non-covalent interactions is more pronounced and provides best surface area for adsorption studies. Reactions were investigated inside the CC2 cage and without CC2 cage. R and TS represents the reactants and transition states without cage whereas R′ and TS′ are the corresponding representations inside the cage.

### 3.1 Trimerization reaction

**Fig. 2:**
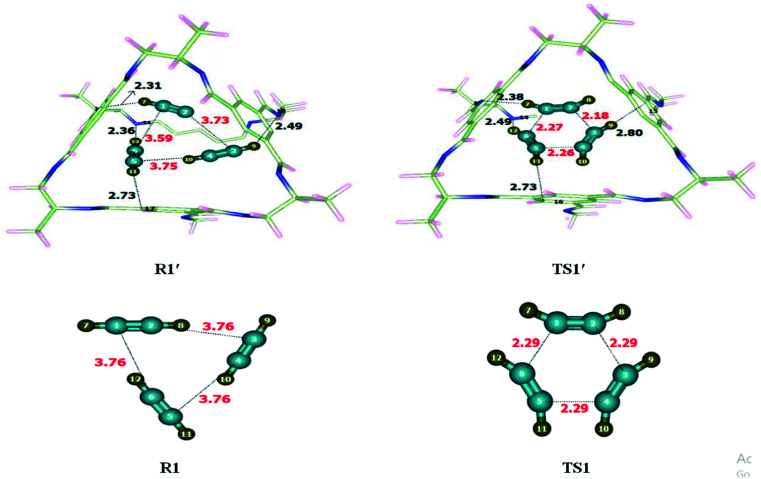
Optimized reactant and transition state for trimerization reaction at M06-2X within and without CC2 cage along with interaction distance and bond lengths. All lengths are in Angstrom (Å).

Alkyne trimerization reaction was theoretically investigated at M06-2X/6-31G(d) level of theory without and within CC2 cage. After encapsulation of R1 inside the CC2 cage, non-covalent interactions have been generated between inner surface of CC2 cage and the encapsulated molecules. The generated non-covalent interactions include strong non-covalent interactions such as hydrogen bonding as well as weak non-covalent interactions such as van der wall forces. H atoms of acetylene molecules show multiple interactions with the atoms of CC2 cage. H atoms interact through H-bond as well as van der Waals interactions with the C atoms of CC2 cage for which interaction distances range from 2.4 Å to 3.9 Å. At the same time, H atoms of acetylene interacts with the N of CC2 cage through H-bond for which the interaction distances range from 2.3 Å to 2.7 Å. Table 1 contains some important interaction distance of TS1′ and R1′. The nature and number of non-covalent interactions in R1′ and TS1′ are different at some points while they are similar at certain other points. For example, H7⋯N13 and H12⋯N14 are the common the interaction interactions. The interaction length of H7⋯N13 is 2.31 Å in R1′ while this interacting distance becomes 2.38 Å in TS1′; in R1′ and TS1′ while this interacting distance becomes 2.38 Å in TS1′; similarly H12⋯N14 interaction distance in R1′ is 2.36 Å while this interaction distance increases to 2.49 Å in TS1′. Some interactions in are different in R1′ than those of TS1′. In R1′ the H11⋯C17 and H9⋯C19 interaction distances are 2.73 Å and 2.49 Å. In TS1′ H9⋯C15 and H11⋯C16 interaction distances are 2.80 Å and 2.73 Å, respectively (Fig. 2). Hydrogen atoms of TS1′ interact with cage at more than one point. The six membered transition state (TS1′) is more stabilized inside the cage as compared to R1′. This stabilization of TS1′ leads to drop in activation energy of the of trimerization reaction.

List of some selected interaction bond lengths of trimerization and cheletropic reactions

**Table:** 

Trimerization reaction					
R1′	Interactions	H7⋯N13	H12⋯N14	H11⋯C17	H9⋯C19
	Interaction distances (Å)	2.31	2.36	2.73	2.49
TS1′	Interactions	H7⋯N13	H12⋯N14	H9⋯C15	H11⋯C16
	Interaction distances (Å)	2.38	2.49	2.80	2.73

**Table:** 

Cheletropic reaction					
R2′	Interactions	H8⋯C15	H9⋯C14	H10⋯C16	H12⋯C17
	Interaction distances (Å)	3.14	2.99	2.69	2.91
TS2′	Interactions	H8⋯C18	H9⋯C14	H10⋯C16	H12⋯C17
	Interaction distances (Å)	2.85	2.79	2.69	2.54

Interactions of the host affect the geometric parameters of TS1′ which appear different than the geometric parameters of TS1 (without cage). For example, C1–C6 bond length is 2.29 Å in TS1 which is reduced to 2.27 Å in TS1′. C2–C3 bond length is 2.29 Å in TS1 which is reduced to 2.18 Å in TS1′. Similarly, C5–C4 bond length in TS1 is 2.29 Å while, this bond length is reduced in TS1′ (2.26 Å). Encapsulation has caused the TS1′ to become early in nature as compared to TS. Their early nature of TS1′ leads to drop in activation barrier.

The activation barrier of the reaction has been calculated. The activation barrier (*E*_a_) for trimerization reaction without cage was 49.40 kcal mol^−1^ whereas it is reduced to 47.60 kcal mol^−1^ in the cage. The result showed an appreciable drop in the energy barrier of trimerization reaction within the cavity of the CC2 cage because of non-covalent interactions between CC2 and guest molecules. The total energy of reactants (*E*_R_) with and without host cage are −165.03 kcal mol^−1^ and −160.70 kcal mol^−1^ respectively.

### 3.2 Cheletropic reaction

**Fig. 3:**
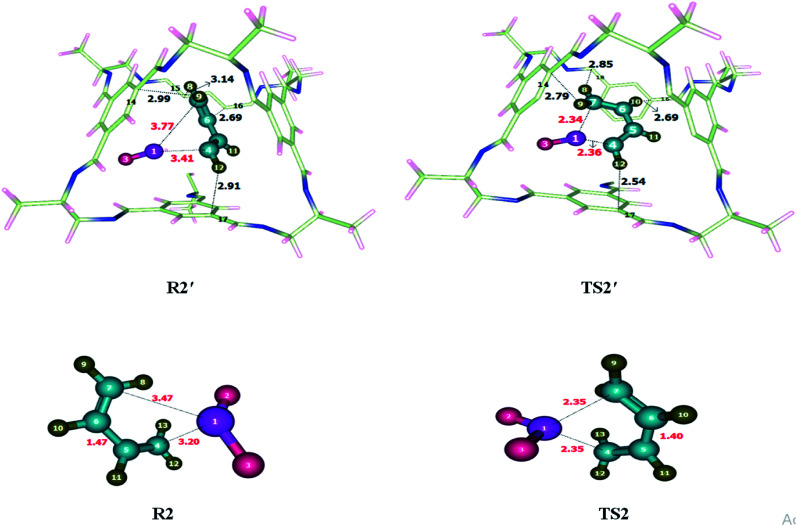
Optimized transition state for cheletropic reaction at M06-2X within and without CC2 cage along with interaction distance and bond lengths.

Addition of sulfur dioxide on butadiene was theoretically investigated. Reactants were encapsulated inside CC2 cage which resulted in the generation of non-covalent interactions including hydrogen bonding and van der Waals forces. Details of non-covalent interactions are explained in NCI analysis. Hydrogen bond distances are usually between 2.7–3.3 Å while interaction distances for van der Waals force lie in the range of 3.3 Å to 4.0 Å (see Fig. 3). Both types of interactions have been observed during the study of cheletropic reaction inside the cage. O atoms of SO_2_ interacts with H atoms of CC2 cage through H-bond at the distance of 2.5–3.0 Å. H atoms of butadiene also interact with the C atoms of CC2 cage through H-bond for which interaction distances range from 2.5–3.3 Å. van der Waals interactions distance between H atoms of butadiene and N atoms of CC2 cage range from 3.2 Å to 3.9 Å. Some important interactions have been reported in Table 1. The nature and number of non-covalent interaction almost remain same for R2′ and TS2′ variations in interaction distances are observed. H9⋯C14 and H12⋯C17 interaction distances are reduced from 2.99 Å and 2.91 Å in R2′ to 2.79 Å and 2.54 Å in TS2′; respectively. Whereas the interaction length of H10⋯C16 remains 2.69 Å in both R2′ and TS2′. H8⋯C15 interaction distance is 3.14 Å in R2′; whereas, in TS2′, the interaction of H8 is shifted from C15 to C18 with reduced interaction distance of 2.85 Å. Overall the non-covalent interactions are more pronounced in TS2′ as compared to R2′ (Fig. 3). This stronger interaction of CC2 cage with TS2′ result in lowering of activation barrier. The geometric parameters of TS2 are also different than those of TS2′. S1–C7 bond length of TS2 is reduced from 2.35 Å to 2.34 Å in TS2′ while S1–C4 bond length of TS2 is 2.35 Å which is slightly increased to 2.36 Å in TS2′.

The activation barrier (*E*_a_) for sigmatropic reaction without cage was calculated as 17.09 kcal mol^−1^ where it is 14.26 kcal mol^−1^ inside CC2 cage. The result showed a significant drop in the energy barrier of cheletropic reaction within the cavity of the CC2 cage mainly due to favorable interactions of transition state (TS2′) with the guest molecules. The energy of reaction (*E*_R_) was calculated as −15.68 kcal mol^−1^ without host cage and −12.88 kcal mol^−1^ within the CC2 cage.

### 3.3 Dyotropic reaction

**Fig. 4:**
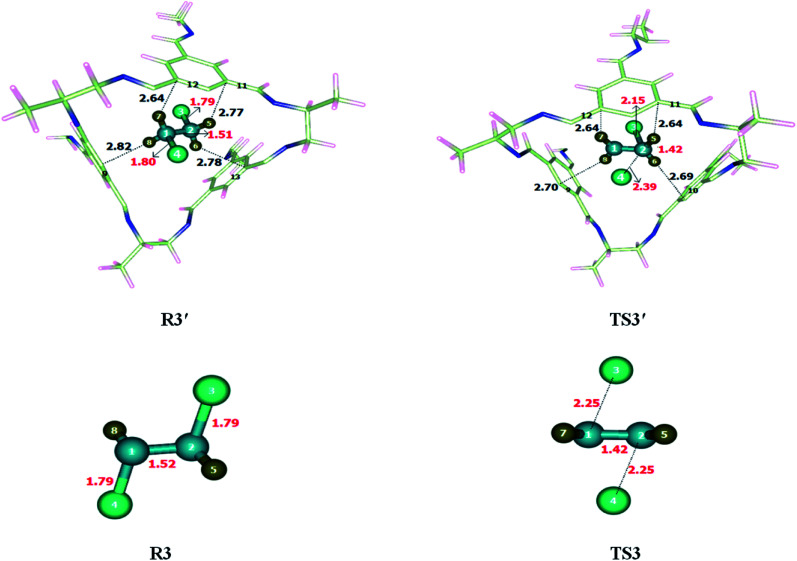
Optimized reactants and transition states for dyotropic reaction at M06-2X within and without CC2 cage along with interaction distance and bond lengths. All bond lengths are in Angstrom (Å).

Dyotropic reaction of di-bromo ethane was investigated inside CC2 cage and without CC2 cage. Noncovalent interactions were generated between the inner surface of the cage and encapsulated molecules. Both strong (hydrogen bond) and weak non-covalent (van der Waals) interactions were observed. Br and H of di-bromo ethane interact with the C and N of CC2 cage through van der Waals forces. The interaction distances for the interaction of Br and H atoms of di-bromo and C and N atoms of CC2 are in the range of 3.4–3.9 Å and 3.5–3.9 Å, respectively. Besides van der Waals forces, H atoms of di-bromo ethane interact with C atoms of CC2 cage through H-bond in the range of 2.6–3.5 Å. Some important interactions have been reported in Table 2. Nature and number of interactions in TS3′ are different than those of R3′. At some points, in R3′ and TS3′ the nature of interacting atoms remain same, but they differ in terms of interaction distances (Fig. 4). For example, H5⋯C11 interaction distance is 2.77 Å in R3′ which is reduced to 2.64 Å in TS3′. H6⋯C13, H7⋯C12, H8⋯C9 interaction distances are 2.78 Å, 2.64 Å, 2.82 Å, respectively in R3′ whereas H6⋯C10, H7⋯C19, H8⋯C12 interaction distances are 2.69 Å, 2.70 Å, 2.64 Å in TS3′, respectively. The non-covalent interaction distances in TS3′ are much shorter than those of R3′ which illustrates the stability of TS3′ in the cage (compared to R3′).

List of some selected interaction bond lengths of dyotropic and ring-expansion and contraction reaction

**Table:** 

Dyotropic reaction					
R3′	Interactions	H5⋯C11	H6⋯C13	H7⋯C12	H8⋯C9
	Interaction distances (Å)	2.77	2.78	2.64	2.84
TS3′	Interactions	H5⋯C11	H6⋯C10	H7⋯C9	H8⋯C12
	Interaction distances (Å)	2.64	2.69	2.70	2.64

**Table:** 

Ring-expansion and contraction reaction					
R4′	Interactions	H10⋯C14	H8⋯C15	H12⋯C16	H13⋯N17
	Interaction distances (Å)	2.71	3.11	2.97	2.67
TS4′	Interactions	H10⋯C14	H12⋯C17	H8⋯N18	H9⋯N19
	Interaction distances (Å)	3.03	2.88	2.73	2.76

The geometric parameters of TS3′ (inside cage) are different than those of TS3 (without cage) at the reaction site. For example, the C2–Br3 bond length in TS3 is 2.25 Å which is reduced to 2.15 Å in TS3′, whereas C1–Br4 and C2–Br4 bond lengths are 2.25 Å each in TS3 which are elongated to 2.39 Å in TS3′.

The observed activation energy (*E*_a_) of the reaction is 56.06 kcal mol^−1^ without the cage and 52.88 kcal mol^−1^ inside the CC2 cage. The activation barrier of the dyotropic reaction is significantly dropped inside the CC2 cage. The calculated total energy of reaction (*E*_R_) is 0 kcal mol^−1^ without cage due to identical reactants and products. Whereas in case of reaction in CC2 the surrounding interactions are different, which results in differences in geometry of R3′ and TS3′. The energy of reactant in this case is −0.13 kcal mol^−1^.

### 3.4 Ring-expansion and contraction reaction

Ring-expansion and contraction reaction was theoretically investigated at M06-2X/6-31G(d) level of theory. Non-covalent interactions such as hydrogen bond and van der Waals forces have been observed when reactant was encapsulated inside the CC2 cage. H atoms of cycloheptatriene interacts with the C and N of CC2 through H-bonds for which interaction distance range from 2.7–3.5 Å and 2.6–3.4 Å, respectively. O atom of cycloheptatriene interacts with the H atoms of CC2 cage through van der Waals forces appearing in the range of 3.1–3.7 Å. The nature and numbers of non-covalent interactions in TS4′ are different than those in R4′ except the interaction between H10⋯C14. H10⋯C14 interaction distance is 2.71 Å in R4′ which is increased to 3.03 Å in TS4′. H8⋯C15, H12⋯C17 and H13⋯N17 interactions distances are 3.11 Å, 2.97 Å and 2.67 Å in R4′, respectively. Nonbonding interactions H12⋯C17, H8⋯N18 and H9⋯N19 for which interaction distances are 2.88 Å, 2.73 Å and 2.76 Å in TS4′, respectively (Table 2). The interaction distances in TS4′ are shorter than those of R4′ which leads to drop in activation barrier. Strengthened in case of TS4′ CC2 cage has stabilized the TS4′ more as compared to R4′. This favorable interaction leads to the drop activation barrier inside the CC2 cage.

**Fig. 5:**
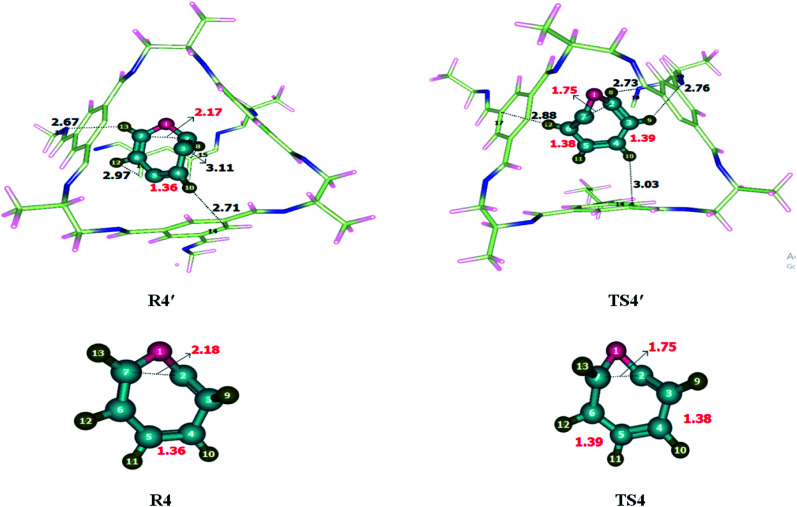
Optimized reactants and transition states for cycloheptatriene–norcaradiene rearrangement at M06-2X within and without CC2 cage along with interaction distance and bond lengths. All bond lengths are in Angstrom (Å).

The geometric parameters of both transition states (TS4 and TS4′) are not significantly different from each other (Fig. 5). C3–C4 bond length is 1.38 Å in TS4 which is elongated to 1.39 Å in TS4′ while the C5–C6 bond length is 1.39 Å in TS4 which is slightly reduced to 1.38 Å in TS4′. The C2–C7 bond length remains 1.75 Å in both TS4 and TS4′. Geometric parameters analysis reveals that the nonbonding interactions are mainly responsible for drop activation barrier. The nonbonding interactions are stronger for TS4′ then R4′.

The activation barrier (*E*_a_) for cycloheptatriene–norcaradiene rearrangement without cage is 28.81 kcal mol^−1^ while it is 26.99 kcal mol^−1^ inside the cage. The result showed a reasonable drop in the energy barrier of cycloheptatriene–norcaradiene rearrangement within the cavity of the CC2 cage. The energy of reaction (*E*_R_) is calculated as 15.43 kcal mol^−1^ without host cage and 12.21 kcal mol^−1^ within the CC2 cage.

### 3.5 1,5-Sigmatropic shift reaction

**Fig. 6:**
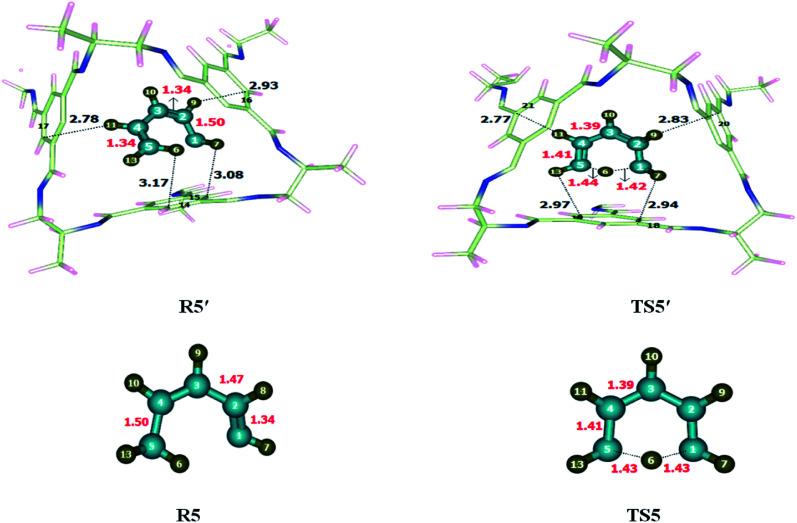
Optimized reactants and transition states for 1,5-sigmatropic shift reaction at M06-2X within and without CC2 cage along with interaction distance and bond lengths. All bond lengths are in Angstrom (Å).

1,5-Sigmatropic was investigated within and without CC2 cage. Atoms of R5′ interact with the inner surface of CC2 cage through non-covalent interactions such as hydrogen bond (2.7–3.3 Å) and van der Waal forces (3.3–4.00 Å). Both types of interactions have been observed during the study of complex (CC2 cage and encapsulated molecule), whereas the interactions lengths vary in each case. H atoms of 1,3 diene interacts with C and N atoms of CC2 cage through H-bonding and van der Waals forces whereas, the interaction lengths in each case inside the complex are 2.6–3.5 Å and 3.2–3.7 Å, respectively. The modes of interactions in R5′ are modified in case of TS5′ (Fig. 6). The interaction distances of H6⋯C15, H7⋯C14, H9⋯C16 and H11⋯C17 in R5′, are 3.08 Å, 3.17 Å, 2.93 Å and 2.78 Å respectively. In TS5′ interaction distance of H7⋯C18, H9⋯C20 Å, H11⋯C21, H13⋯C16 are 2.94 Å, 2.83 Å, 2.77 Å and 2.97 Å, respectively. Some important interaction distances are mentioned in Table 3.

Illustration of some selected interaction bond lengths of 1,5-sigmatropic shift and 6-π electron cyclization reaction

**Table:** 

1,5-Sigmatropic shift reaction					
R5′	Interactions	H6⋯C15	H7⋯C14	H9⋯C16	H11⋯C17
	Interaction distances (Å)	3.08	3.17	2.93	2.78
TS5′	Interactions	H7⋯C18	H9⋯C20	H11⋯C21	H13⋯C19
	Interaction distances (Å)	2.94	2.83	2.77	2.97

**Table:** 

6-π electron cyclization reaction					
R6′	Interactions	H10⋯C14	H8⋯C15	H12⋯C16	H13⋯N17
	Interaction distances (Å)	2.71	3.11	2.97	2.67
TS6′	Interactions	H14⋯C15	H12⋯N16	H11⋯C17	H7⋯C18
	Interaction distances (Å)	2.94	2.63	2.79	2.96

There is no significant difference observed in the bond lengths of TS5 and TS5′ except C1–H6 and C5–H6 which are 1.43 Å in TS5, C1–H6 bond length is reduced to 1.42 Å whereas C1–H6 bond length is elongated to 1.44 Å in TS5′ while remaining bond lengths remained unchanged.

The observed activation energies of the reaction are 36.34 kcal mol^−1^ (without cage) and 36.82 kcal mol^−1^ for the reaction inside the CC2 cage. The exothermicity of 1–5 sigma tropic shift are calculated 0 kcal mol^−1^ without cage and −0.33 kcal mol^−1^ (inside the CC2 cage). The activation barriers for both reactions (without CC2 cage and inside CC2 cage) are almost similar which is attributed to similar strength of interactions in R5′ and TS5′.

### 3.6 6-π electron cyclization reaction

**Fig. 7:**
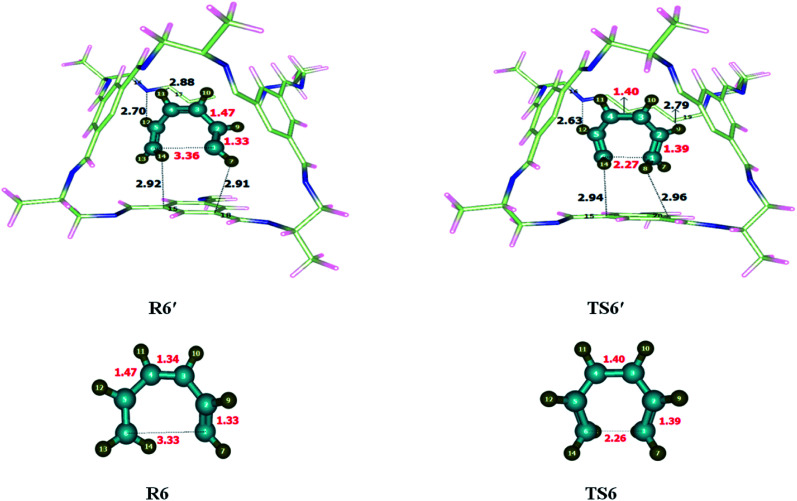
Optimized reactant transition states for 6-π electrocyclization reaction at M06-2X within and without CC2 cage along with interaction distance and bond lengths. All bond lengths are in Angstrom (Å).

Optimization of the reactants and transition state of 6-π electro-cyclization reaction performed at M06-2X/6-31G(d) level of theory within and without CC2 cage. The values of interactions lengths showed the presence of non-covalent interactions between CC2 cage and encapsulated molecules. Interactions distances for R6 are between 2.7 Å to 4.00 Å which indicate the presence of hydrogen bond and van der Waals forces inside the complex. H atoms of 1,3,5-hexatriene shows multiple interactions with the C atoms and N atoms of CC2 cage through H-bond. The nature and number of interactions are different in R6′ and TS6′. H12⋯N16, H14⋯C15, H11⋯C17 and H7⋯C18 distances in R6′ are 2.70 Å, 2.88 Å and 2.91 Å, respectively (Fig. 7). H14⋯C15 and H12⋯N16 are common interactions in both R6′ and TS6′ for which distances are 2.92 Å, 2.70 Å in R6′ whereas 2.94 Å and 2.63 Å in TS6′. H9⋯C19 and H8⋯C20 interaction distances are 2.79 Å and 2.96 Å in TS6′. Table 3 illustrates some important interaction lengths of R6′ and TS6′. The results show that the interaction strength in both R6′ and TS6′ are almost comparable due to which both R6′ and TS6′ have almost same stabilities. This is the reason the net result of interactions in R6′ and TS6′ are cancelled out and no valuable change is observed in the energy of activation.

The geometric parameters of TS6 and TS6′ are shown in Fig. 7. Almost all geometric parameters of TS6 and TS6′ are same except C1–C6 bond length which is 2.26 Å in TS6 while 2.27 Å in TS6′.

The observed activation energies (*E*_a_) of the reaction are 20.44 kcal mol^−1^ without the cage and 20.10 kcal mol^−1^ for the reaction inside the CC2 cage. The activation barriers for both reactions (without CC2 cage and inside CC2 cage) are almost similar. It is due to the same strength of interactions of the cage in R6′ and TS6′. The exothermicity of 6-π electro-cyclization reaction are calculated where the calculated value of *E*_R_ of the selected reaction is −26.87 kcal mol^−1^ without cage and −28.49 kcal mol^−1^ for the reaction inside the CC2 cage.

### 3.7 Claisen rearrangement reaction

**Fig. 8:**
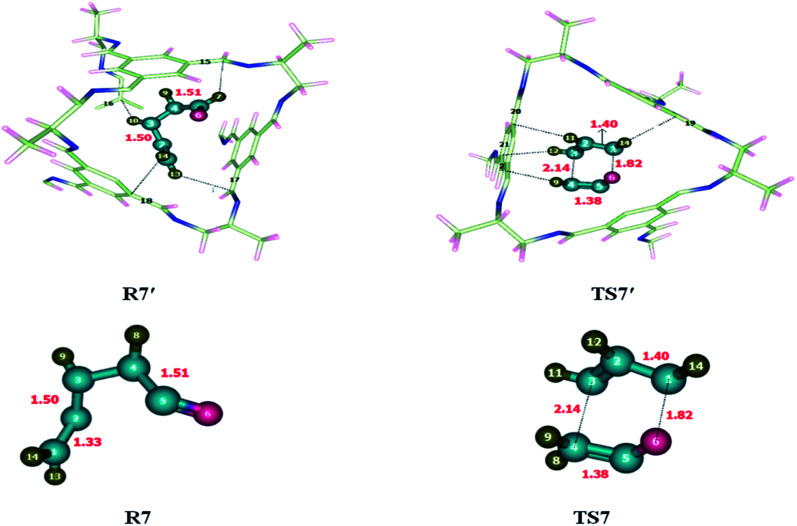
Optimized reactants and transition states for Claisen rearrangement reaction at M06-2X within and without CC2 cage along with interaction distance and bond lengths. All bond lengths are in Angstrom (Å).

To investigate the energetics of Claisen rearrangement, we selected chloroallyl vinyl ether as a reactant for reaction. After encapsulation the reactant atoms such as H, C and O interacts with the atoms of CC2 cage such as N, C and H. The interactions distances (Angstrom Å) are indicative of non-covalent interactions such as hydrogen bond and van der Waals forces. H atoms of allyl vinyl ether interact with the C and N atoms of CC2 cage through H-bonding. O atom of allyl vinyl ether interacts with the H atoms of CC2 cage through van der Waals interactions in the range of 3.1 Å to 2.8 Å. Some important interactions distances are highlighted in Table 4. Interaction distances of H132⋯C81, H132⋯C98, H137⋯C61 and H139⋯H40 are 3.54 Å, 3.01 Å, 2.90 Å and 2.71 Å in R7′, respectively. In TS7′ all new interactions were generated as compared to R7′. The interaction distances of H132⋯C81, H132⋯C98, H137⋯C61 and H139⋯H40 in TS7′ are 2.70 Å, 2.76 Å, 2.59 Å and 2.68 Å, respectively (see Table 4). Interaction generated by CC2 cage stabilized the encapsulated guest molecules (Fig. 8) compared to TS7. The nonbonding interaction analysis reveals that the nonbonding interactions are stronger in R7′ as compared to TS7′, which indicates the higher stability of R7′ in CC2 cage then TS7′. This stability of R7′ resulted in increase in activation barrier. There is no significant change has been observed in the bond lengths of transition states outside of the cage and inside the cage. All bond lengths remain same for TS7 and TS7′.

Illustration of some selected interaction bond lengths of Claisen rearrangement and keto-enol tautomerization reaction

**Table:** 

Claisen rearrangement reaction					
R7′	Interactions	H132⋯C81	H132⋯C98	H137⋯C61	H139⋯H40
	Interaction distances (Å)	3.54	3.01	2.90	2.71
TS7′	Interactions	H132⋯C81	H132⋯C98	H137⋯C61	H139⋯H40
	Interaction distances (Å)	2.70	2.76	2.59	2.68

**Table:** 

Keto-enol tautomerization reaction					
R8′	Interactions	H8⋯C16	H10⋯C15	H11⋯C17	H12⋯C18
	Interaction distances (Å)	3.01	2.83	2.37	2.35
TS8′	Interactions	H8⋯C16	H10⋯C15	H11⋯C14	H12⋯C13
	Interaction distances (Å)	3.01	2.89	2.98	2.33

Unusual behavior is observed by the Claisen rearrangement due to structural features and modified interactions distances in R7′ and TS7′. The observed activation energies are 47.82 kcal mol^−1^ without the cage and 51.24 kcal mol^−1^ for the reaction inside the CC2 cage. The result shows increase in the energy barrier of Claisen rearrangement inside CC2 cage. The calculated *E*_R_ is 17.64 kcal mol^−1^ for reaction without cage and 18.32 kcal mol^−1^ inside the CC2 cage.

### 3.8 Comparison of keto-enol tautomerism reaction within and without CC2 cage

To investigate the keto-enol tautomerism reaction, reaction was carried out individually without cage as well as inside the selected CC2 cage. Studied activation barrier for individual reaction without cage is 27.71 kcal mol^−1^ and total energy of reaction is −13.61 in kcal mol^−1^. After encapsulation H, C and O atoms of reactants interacts through non-covalent interactions such as hydrogen bond and van der Waals forces with the atoms of CC2 cage such as N, C and H. whereas, the interaction lengths in each case are in between 2.6–3.5 Å and 3.1–3.7 Å, respectively. O atoms of the reactant interact with the C atoms of CC2 cage through van der Waals interaction range from 3.1 Å to 3.8 Å.

Interactions in R8′ are different than the interactions in TS8′ except some interactions. For example, H8⋯C16 and H10⋯C15 are the interactions which are common in both R8′ and TS8′. H8⋯C16 interaction distance is 3.01 Å in both R8′ and TS8′ while H10⋯C15 interaction distance is 2.83 Å in R8′ which is increased to 2.89 Å in TS8′. H11⋯C17, H12⋯C1815 interactions distances are 2.37 Å and 2.35 Å in R8′, respectively. H11⋯C14, H12⋯C13 interactions distances are 2.98 Å and 2.33 Å in TS8′, respectively. Interaction distances of R8′ and TS8′ are shown in Table 4. Based on interactions, the stability of R8′ and TS8′ is almost equal due to which no significant change is observed in the energy barrier of reaction inside the cage and without cage.

**Fig. 9:**
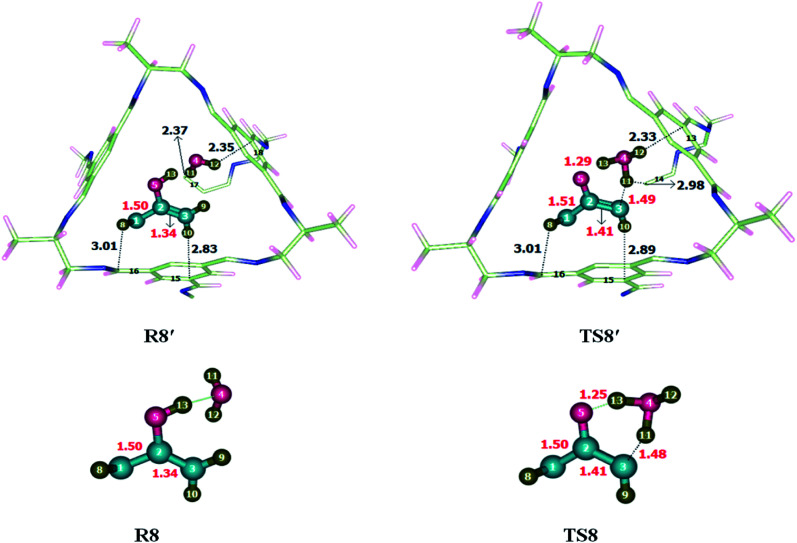
Optimized reactants transition states for keto-enol tautomerism reaction at M06-2X within and without CC2 cage along with interaction distance and bond lengths. All bond lengths are in Angstrom (Å).

The geometric parameters of TS8 are different than those of TS8′. C3–H11, O5–H13 and C1–C2 bond lengths are 1.48 Å, 1.25 Å and 1.50 of TS8 which are increased to 1.49 Å, 1.29 Å and 1.51 Å in TS8′. C2–C3 bond lengths remain same in both TS8 and TS8′ which is 1.40 Å each (Fig. 9).

The observed activation energies are 27.67 kcal mol^−1^ without the cage and 27.97 kcal mol^−1^ for the reaction inside the CC2 cage. In this case, both R8′ and TS8′ have almost same stabilities inside the cage, due to which the energy barrier of keto-enol tautomerism inside the cage is almost same as calculated for reaction without cage. Calculated values of *E*_R_ of the keto-enol tautomerism are −13.61 kcal mol^−1^ without cage and −15.97 kcal mol^−1^ for the reaction inside the CC2 cage.

### 3.9 [2 + 4] cycloaddition reaction

To investigate the kinetics of [2 + 4] cycloaddition reaction, the reactants and transition state were optimized at M06-2X/6-31G(d) level of theory. Reaction was studied individually in its gaseous state as well as inside the selected CC2 cage. Butadiene and ethene (R9) were encapsulated inside the optimized CC2 cage. Atoms of R9′ (H, C) interact with the inner surface of CC2 cage which behaves as guest for the encapsulated molecules through non-covalent interactions such as hydrogen bond and van der Waals forces. H atoms of butadiene and ethane interact with the C and N atoms of CC2 cage through H-bond. Where interaction lengths are 2.6–3.0 Å and 2.6–3.5 Å, respectively.

**Fig. 10:**
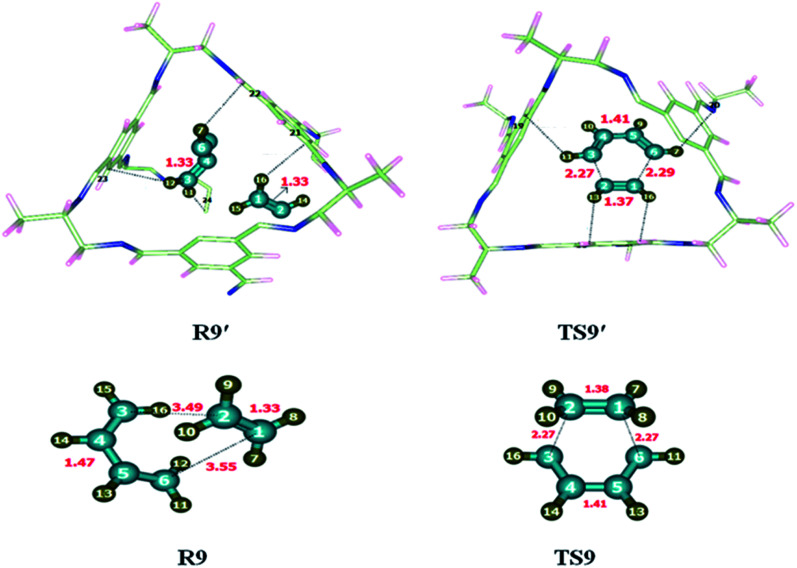
Optimized reactants transition states for [2 + 4] cycloaddition reaction at M06-2X within and without CC2 cage along with interaction distance and bond lengths. All bond lengths are in Angstrom (Å).

The nature and number of non-covalent interactions in R9′ and TS9′ are differing at certain points. H133⋯C24, H135⋯C76, H137⋯C57 and H137⋯C34 interaction distances in TS9 are 2.76 Å, 2.75 Å, 2.81 Å and 2.63 Å, respectively. The interaction bond distances of H133⋯C24, H135⋯C26, H137⋯C57 and H137⋯C34 are 2.81 Å, 3.43 Å, 2.81 and 3.18 Å, respectively inTS9′ (see Table 5). Overall, the interaction distances of R9′ are illustrating strong interaction with CC2 cage which results in the stability of R9′ as compared to TS1′. Due to the more stable reactant activation barrier inside the CC2 cage increased as compared without cage (Fig. 10).

**Table:** Comparison of selected bond lengths of TS9 and TS9′. All bond lengths are in Angstrom (Å)

[2 + 4] cycloaddition reaction					
R9′	Interactions	H133⋯C24	H135⋯C26	H137⋯C57	H137⋯C34
	Interaction distances (Å)	2.81	3.43	3.41	3.18
TS9′	Interactions	H133⋯C24	H135⋯C76	H137⋯C57	H137⋯C34
	Interaction distances (Å)	2.76	2.75	2.81	2.63

The geometric parameters of TS9 are different than TS9′. For example, C1–C2 bond length is 1.38 Å in TS9 which is decreased to 1.37 Å in TS9′. C1–C6 bond length is 2.27 Å in TS9 which is elongated to 2.29 Å in TS9′. Whereas C2–C3 and C4–C5 bond lengths are 1.41 Å each in TS9 and TS9′.

The observed activation energies of the reaction are 17.62 kcal mol^−1^ without the cage and 19.47 kcal mol^−1^ for the reaction inside the CC2 cage. The higher barrier is due to the greater stability of the reactant inside the cage compared to transition state. Calculated value of *E*_R_ of the 2 + 4 cycloaddition reaction is −47.86 kcal mol^−1^ without cage and −50.25 kcal mol^−1^ for the reaction inside the CC2 cage.

### 3.10 Non-covalent interaction (NCI) analysis

**Fig. 11:**
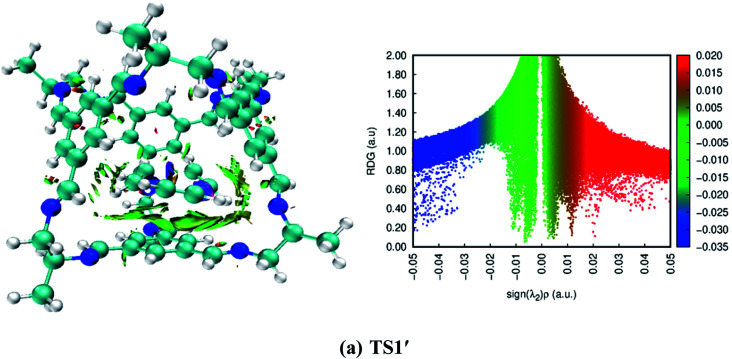
3D-isosurfaces and 2D-RDG graphs at M06-2X of transition states inside CC2 cage for trimerization reaction.

To understand the type of interactions that generated between the CC2 cage molecule and reactants, we performed NCI analysis. Two types of parameters (3D isosurface and 2D reduced gradient density graph) were generated to characterize the type of interactions. The obtained 3D isosurface (NCI of reactions) generally there are two types of non-covalent interactions are found hydrogen-bonding and van der Waals forces. NCI plots reveal the presence of strong hydrogen-bonding and van der Waals forces in all organic transformations. In the 2D reduced density graph, three types of interactions are appeared *i.e.*, blue patches show hydrogen bonding, red patches show repulsion forces and green patches shows non-covalent interactions. The green patches in the 2D-RDG graph ranging from 0.000 a.u. to 0.015 a.u. confirm the presence of van der Waals forces. Slight blue patches are also observed in all organic transformations except 1,5-sigmatropic shift reaction, 6-π electron cyclization reaction and keto-enol tautomerism reaction, which is responsible for hydrogen bonding. 2D-RDG graph also confirms the presence of hydrogen bonding in the above-mentioned reactions (see Fig. 11 and S1 (ESI†))

The obtained 3D isosurface and RDG-2D graph for trimerization reaction is presented in Fig. 11. While for the rest of organic transformations NCI 3D isosurface and RDG-2D graphs are given in ESI (Fig. S1†). The green patches between H17, H12, H9, H11 of host TS1′ and N13, N14, C15, C16 CC2 of guest shows non-covalent interactions. While the 2D reduced density graph shows the green scattered patches ranging from 0.000 a.u. to −0.015 a.u. confirms the presence of van der Waals forces while the wide green patch in the graph shows strong non-covalent interactions. The blue patches ranging from −0.03 to −0.05 indicates the hydrogen bonding.

## 4 Conclusions

In summary we have studied various organic transformation in confined space of CC2 cage. Mullikan charges show nitrogen as the most electronegative specie whereas carbon is electronegative and hydrogen atoms, the most positive specie of optimized cage. Reactions were investigated theoretically within and without CC2 cage. Reactants after encapsulation interacts with the atoms of CC2 cage through non-covalent interactions such as hydrogen-bond and van der Waals forces. For some reactions such as trimerization, cheletropic reaction, ring expansion reaction, the non-covalent interactions of the transition states and CC2 cage are stronger as compared to the reactants which results in the significant drop of activation barrier. NCI analysis also confirms the presence of strong non-covalent interactions for these reactions. For trimerization reaction and cheletropic reaction the activation energies are 49.40 kcal mol^−1^ and 17.52 kcal mol^−1^ without cage, whereas these barriers are 47.70 kcal mol^−1^ and 15.09 kcal mol^−1^, respectively, in the cage. Moreover, reactions where the strength of non-covalent interactions between cage and guest molecules is almost same for reactants and transition states, no change in activation barrier is observed. The activation barrier for 6-π electrocyclization is 20.44 kcal mol^−1^ and 20.10 kcal mol^−1^ without and within CC2 cage, respectively. Similarly for keto-enol tautomerization the activation barrier is almost same with in the cage and outside of the cage. An unusual behavior has been observed in case of Claisen rearrangement R7′ is more stabilized inside the cage as compared to the TS7′ due to its structural geometry, and as a result, the activation barrier increased inside the cage for Claisen rearrangement.

## Author contributions

Ayesha Mukhtar: methodology, conceptualization, investigation, writing-original draft. Sehrish Sarfaraz: software, validation, visualization. Khurshid Ayub: supervision, writing-review & editing.

## Conflicts of interest

There are no conflicts to declare.

## Supplementary Material

RA-012-D2RA03399B-s001

## References

[cit1] Cho H.-Y. (2012). *et al.*, CO2 adsorption and catalytic application of Co-MOF-74 synthesized by microwave heating. Catal. Today.

[cit2] Li M. (2020). *et al.*, Oxidase-like MOF-818 nanozyme with high specificity for catalysis of catechol oxidation. J. Am. Chem. Soc..

[cit3] Singh M. (2019). *et al.*, Highly active ultrasmall Ni nanoparticle embedded inside a robust metal–organic framework: remarkably improved adsorption, selectivity, and solvent-free efficient fixation of CO_2_. Inorg. Chem..

[cit4] Ma S. (2019). *et al.*, Design of double-component metal–organic framework air filters with PM2. 5 capture, gas adsorption and antibacterial capacities. Carbohydr. Polym..

[cit5] Qian X. (2019). *et al.*, Enhanced visible-light-driven photocatalytic activity of Ag_3_PO_4_/metal–organic framework composite. Polyhedron.

[cit6] Karapınar N. (2009). Application of natural zeolite for phosphorus and ammonium removal from aqueous solutions. J. Hazard. Mater..

[cit7] Li M. (2011). *et al.*, Application of modified zeolite for ammonium removal from drinking water. Desalination.

[cit8] Weitkamp J. (2000). Zeolites and catalysis. Solid State Ionics.

[cit9] Eroglu N., Emekci M., Athanassiou C. G. (2017). Applications of natural zeolites on agriculture and food production. J. Sci. Food Agric..

[cit10] Kraus M. (2018). *et al.*, Competing adsorption of toluene and water on various zeolites. Chem. Eng. J..

[cit11] Abdelrasoul A. (2017). *et al.*, Applications of molecular simulations for separation and adsorption in zeolites. Microporous Mesoporous Mater..

[cit12] Cacho-Bailo F. (2017). *et al.*, Structural contraction of zeolitic imidazolate frameworks: membrane application on porous metallic hollow fibers for gas separation. ACS Appl. Mater. Interfaces.

[cit13] Tozawa T. (2009). *et al.*, Porous organic cages. Nat. Mater..

[cit14] Hasell T., Cooper A. I. (2016). Porous organic cages: soluble, modular and molecular pores. Nat. Rev. Mater..

[cit15] Brutschy M. (2012). *et al.*, Porous organic cage compounds as highly potent affinity materials for sensing by quartz crystal microbalances. Adv. Mater..

[cit16] Hasell T. (2016). *et al.*, Porous organic cages for sulfur hexafluoride separation. J. Am. Chem. Soc..

[cit17] Chen L. (2014). *et al.*, Separation of rare gases and chiral molecules by selective binding in porous organic cages. Nat. Mater..

[cit18] Zhang J.-H. (2015). *et al.*, Highly selective separation of enantiomers using a chiral porous organic cage. J. Chromatogr. A.

[cit19] Ghalami Z., Ghoulipour V., Khanchi A. (2019). Highly efficient capturing and adsorption of cesium and strontium ions from aqueous solution by porous organic cage: a combined experimental and theoretical study. Appl. Surf. Sci..

[cit20] Turner C. H. (2002). *et al.*, Effect of confinement by porous materials on chemical reaction kinetics. J. Chem. Phys..

[cit21] Feng S. (2020). *et al.*, Fabrication of a Hydrogen-Bonded Organic Framework Membrane through Solution Processing for Pressure-Regulated Gas Separation. Angew. Chem., Int. Ed..

[cit22] Mastalerz M. (2011). *et al.*, A salicylbisimine cage compound with high surface area and selective CO_2_/CH_4_ adsorption. Angew. Chem., Int. Ed..

[cit23] Skarmoutsos I. (2020). *et al.*, Porous carbon nanotube networks and pillared graphene materials exhibiting high SF6 adsorption uptake and separation selectivity of SF6/N2 fluid mixtures: a comparative molecular simulation study. Microporous Mesoporous Mater..

[cit24] Wang J. (2014). *et al.*, Effect of nitrogen group on selective separation of CO_2_/N_2_ in porous polystyrene. Chem. Eng. J..

[cit25] Jiang S. (2011). *et al.*, Selective gas sorption in a [2+ 3] ‘propeller’ cage crystal. Chem. Commun..

[cit26] Zhang J. H. (2020). *et al.*, Recent advances of application of porous molecular cages for enantioselective recognition and separation. J. Sep. Sci..

[cit27] Zhang J.-H. (2015). *et al.*, Homochiral porous organic cage with high selectivity for the separation of racemates in gas chromatography. Anal. Chem..

[cit28] Chen T.-H. (2014). *et al.*, Thermally robust and porous noncovalent organic framework with high affinity for fluorocarbons and CFCs. Nat. Commun..

[cit29] Wu X. (2019). *et al.*, Chiral BINOL-based covalent organic frameworks for enantioselective sensing. J. Am. Chem. Soc..

[cit30] Lu Z. (2019). *et al.*, Carbon dot-decorated porous organic cage as fluorescent sensor for rapid discrimination of nitrophenol isomers and chiral alcohols. Anal. Chim. Acta.

[cit31] Zhang Y. (2018). *et al.*, Porous organic cage stabilised palladium nanoparticles: efficient heterogeneous catalysts for carbonylation reaction of aryl halides. Chem. Commun..

[cit32] Sun J.-K. (2015). *et al.*, Toward homogenization of heterogeneous metal nanoparticle catalysts with enhanced catalytic performance: soluble porous organic cage as a stabilizer and homogenizer. J. Am. Chem. Soc..

[cit33] Mondal B. (2016). *et al.*, Molecular cage impregnated palladium nanoparticles: efficient, additive-free heterogeneous catalysts for cyanation of aryl halides. J. Am. Chem. Soc..

[cit34] Li H.-X. (2020). *et al.*, A hydroxyl-functionalized homochiral porous organic cage for gas chromatographic separations. Microchim. Acta.

[cit35] Smith P. T. (2018). *et al.*, Iron porphyrins embedded into a supramolecular porous organic cage for electrochemical CO_2_ reduction in water. Angew. Chem., Int. Ed..

[cit36] Hong S. (2015). *et al.*, Porphyrin boxes: rationally designed porous organic cages. Angew. Chem., Int. Ed..

[cit37] Yang X. (2018). *et al.*, Encapsulating highly catalytically active metal nanoclusters inside porous organic cages. Nat. Catal..

[cit38] Song Q. (2019). *et al.*, Ru nanoclusters confined in porous organic cages for catalytic hydrolysis of ammonia borane and tandem hydrogenation reaction. Nanoscale.

[cit39] Zhai Z. (2020). *et al.*, Advanced nanofiltration membrane fabricated on the porous organic cage tailored support for water purification application. Sep. Purif. Technol..

[cit40] Yang J. (2019). *et al.*, Ultrafine palladium nanoparticles confined in core–shell magnetic porous organic polymer nanospheres as highly efficient hydrogenation catalyst. J. Colloid Interface Sci..

[cit41] Wang W. (2021). *et al.*, Enhancing the activity, selectivity, and recyclability of Rh/PPh_3_ system-catalyzed hydroformylation reactions through the development of a PPh_3_-derived quasi-porous organic cage as a ligand. Chin. J. Catal..

[cit42] Kou J. (2022). *et al.*, Precisely controlled Pd nanoclusters confined in porous organic cages for size-dependent catalytic hydrogenation. Appl. Catal., B.

[cit43] Zhang J.-H. (2020). *et al.*, Ultrafine Palladium Nanoparticles Stabilized in the Porous Liquid of Covalent Organic Cages for Photocatalytic Hydrogen Evolution. ACS Appl. Energy Mater..

[cit44] Young T. A. , *et al.*, Rationalizing the “Diels-Alderase” Activity of Pd2L4 Self-Assembled Metallocages: Enabling the Efficient Prediction of Catalytic Scaffolds, 2019

[cit45] Halls M. D., Schlegel H. B. (2002). Chemistry inside carbon nanotubes: the Menshutkin SN2 reaction. J. Phys. Chem. B.

[cit46] Trzaskowski B., Adamowicz L. (2009). Chloromethane and dichloromethane decompositions inside nanotubes as models of reactions in confined space. Theor. Chem. Acc..

[cit47] Smith N. M., Iyer K. S., Corry B. (2014). The confined space inside carbon nanotubes can dictate the stereo-and regioselectivity of Diels–Alder reactions. Phys. Chem. Chem. Phys..

[cit48] Alves I., Magalhães A. L. (2019). BN-Doped Graphene and Single-Walled Carbon Nanotubes for the Catalysis of SN2 Reactions: Insights from Density Functional Theory Modeling. J. Phys. Chem. A.

[cit49] Frisch A. , Gaussian 09W, Wallingford, USA, 2009, vol. 470, p. 25

[cit50] Zhao Y., Truhlar D. G. (2008). The M06 suite of density functionals for main group thermochemistry, thermochemical kinetics, noncovalent interactions, excited states, and transition elements: two new functionals and systematic testing of four M06-class functionals and 12 other functionals. Theor. Chem. Acc..

[cit51] Cheng Q.-Q. (2017). *et al.*, Cycloaddition reactions of enoldiazo compounds. Chem. Soc. Rev..

[cit52] Shoji M. (2003). *et al.*, Reaction modes of oxidative dimerization of epoxycyclohexenols. Tetrahedron Lett..

[cit53] Fernandez I., Cossio F. P., Sierra M. A. (2009). Dyotropic reactions: mechanisms and synthetic applications. Chem. Rev..

[cit54] Wooi G. Y., White J. M. (2005). Structural manifestations of the cheletropic reaction. Org. Biomol. Chem..

[cit55] Hess B. A., Baldwin J. E. (2002). [1,5] Sigmatropic hydrogen shifts in cyclic 1,3-dienes. J. Org. Chem..

[cit56] Ito H., Taguchi T. (1999). Asymmetric Claisen rearrangement. Chem. Soc. Rev..

[cit57] Jarzȩcki A. A., Gajewski J., Davidson E. R. (1999). Thermal rearrangements of norcaradiene. J. Am. Chem. Soc..

[cit58] Moreno M., Miller W. H. (1990). On the tautomerization reaction 2-pyridone ⇌ 2-hydroxypyridine: an ab initio study. Chem. Phys. Lett..

[cit59] Cioslowski J., Liu G., Moncrieff D. (2000). The concerted trimerization of ethyne to benzene revisited. Chem. Phys. Lett..

