# Supplementary material for: Organic transformations in the confined space of porous organic cage CC2; catalysis or inhibition
Source: RSC Adv. 2022 Aug 26;12(37):24397–411. doi: 10.1039/d2ra03399b (PMC9415023; doi:10.1039/d2ra03399b)
Supplement: RA-012-D2RA03399B-s001 [file RA-012-D2RA03399B-s001.pdf]

Electronic Supplementary Material (ESI) for RSC Advances.
This journal is © The Royal Society of Chemistry 2022

Organic transformations in the confined space of porous organic cage CC2; Catalysis or inhibition

*Ayesha Mukhtar, Sehrish Sarfaraz, & Khurshid Ayub**

Department of Chemistry, COMSATS University, Abbottabad Campus, KPK, Pakistan 22060

*Corresponding author.

Tel: +92-992-383591.

E-mail: khurshid@cuiatd.edu.pk (K.A.)

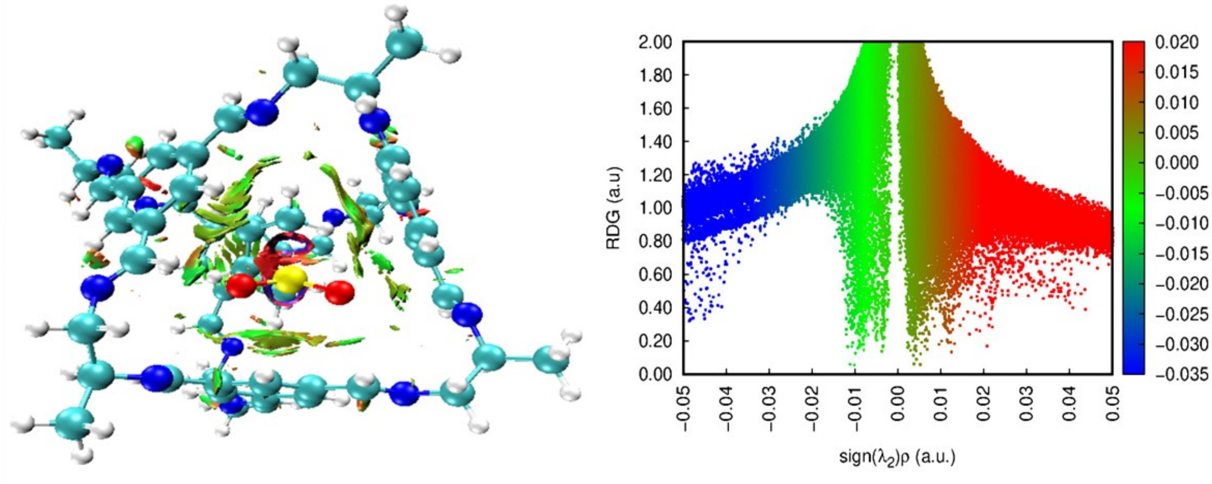

(a) TS2′

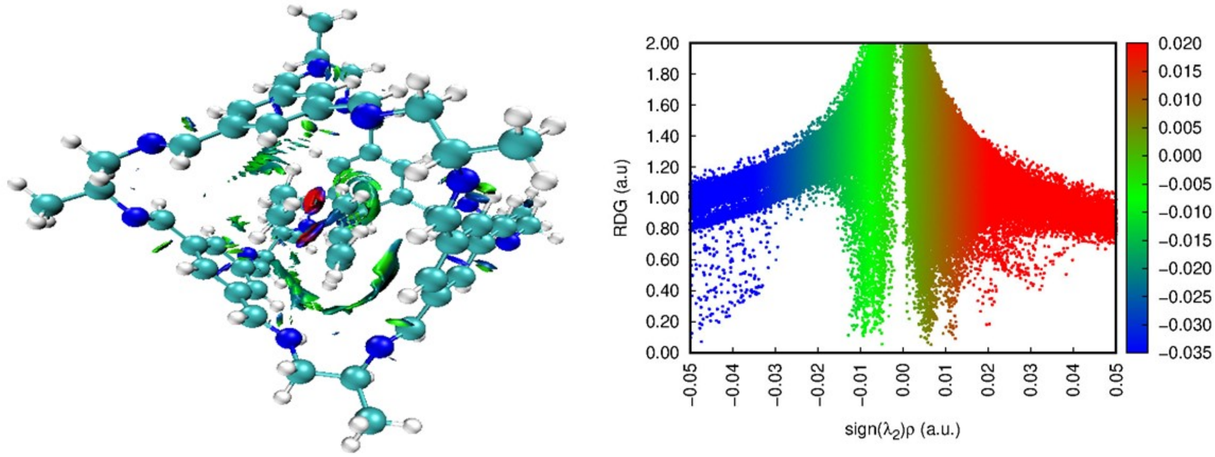

(b) TS3′

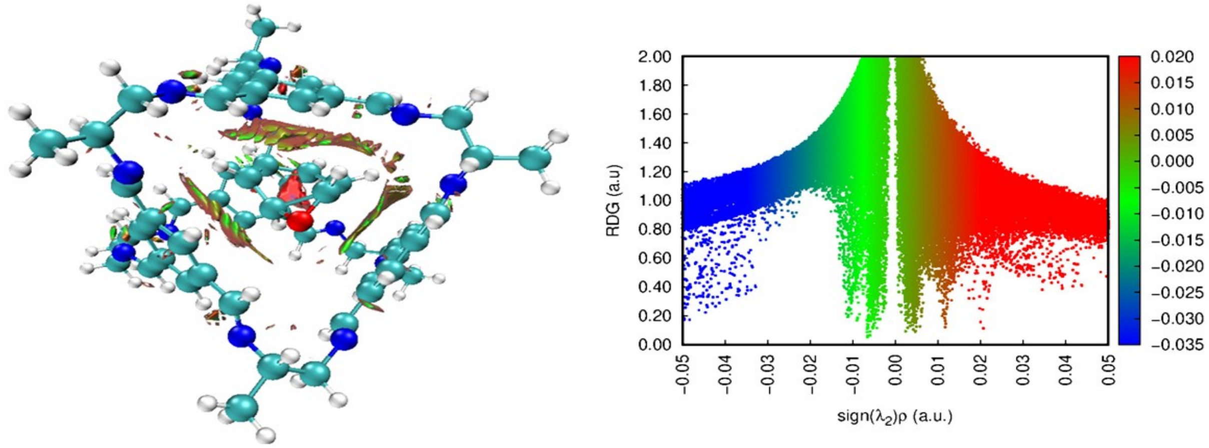

(c) TS4′

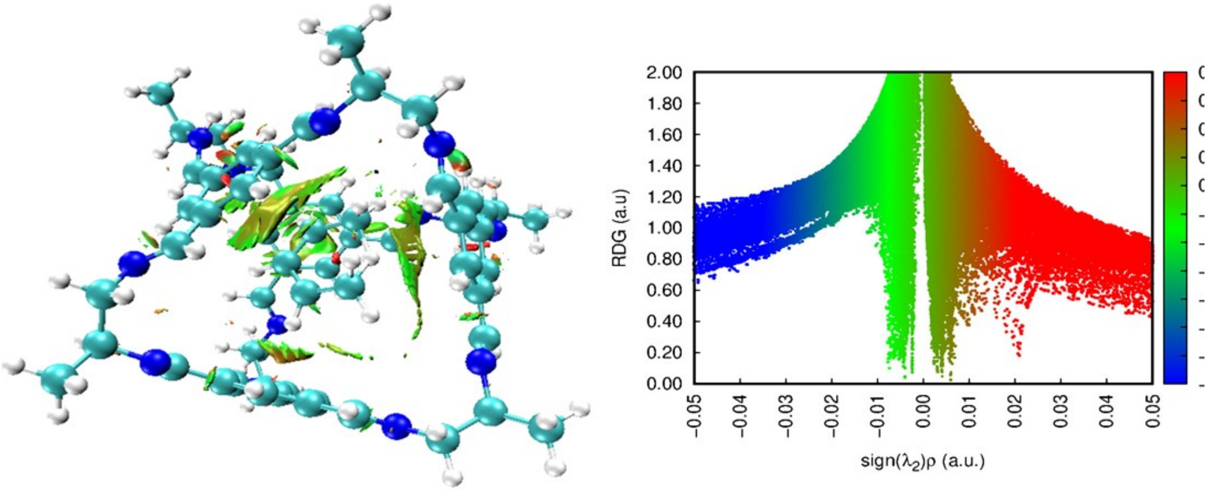
RDG (a.u)
sign(λ2)ρ (a.u.)
2.00
1.80
1.60
1.40
1.20
1.00
0.80
0.60
0.40
0.20
0.00
-0.05
-0.04
-0.03
-0.02
-0.01
0.00
0.01
0.02
0.03
0.04
0.05

(d) TS5′

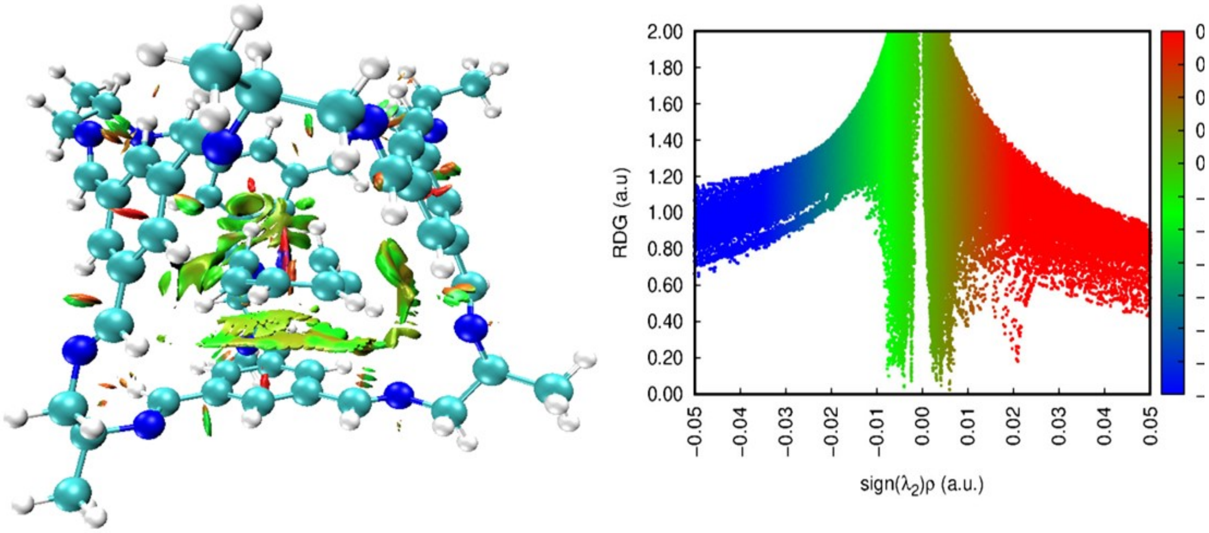
RDG (a.u)
sign(λ2)ρ (a.u.)
2.00
1.80
1.60
1.40
1.20
1.00
0.80
0.60
0.40
0.20
0.00
-0.05
-0.04
-0.03
-0.02
-0.01
0.00
0.01
0.02
0.03
0.04
0.05

(e) TS6′

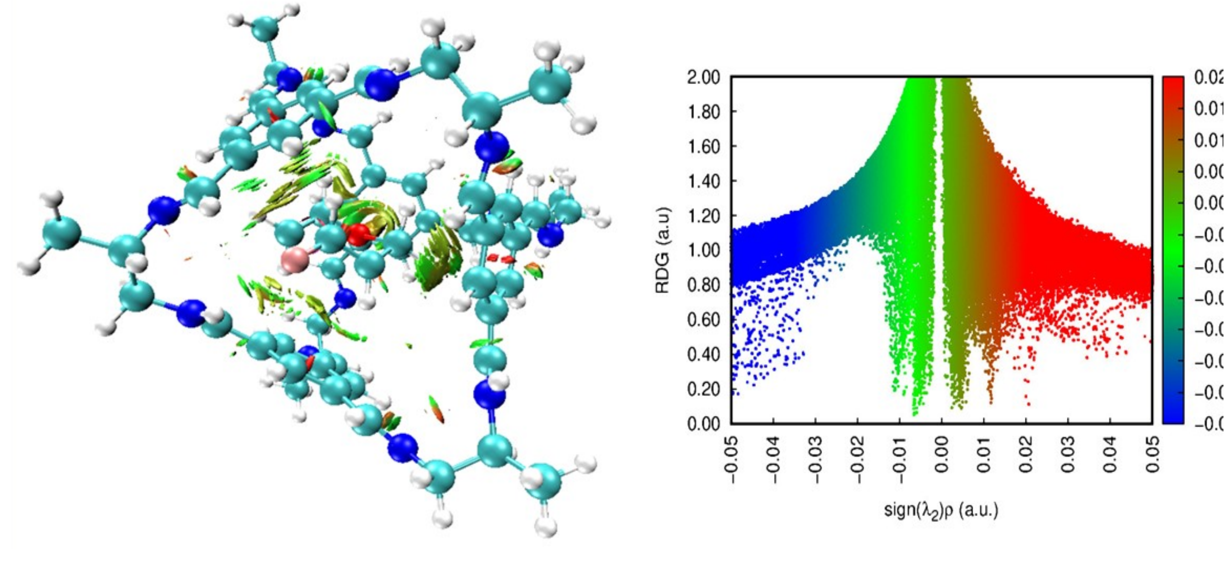
RDG (a.u)
sign(λ2)ρ (a.u.)
2.00
1.80
1.60
1.40
1.20
1.00
0.80
0.60
0.40
0.20
0.00
−0.05
−0.04
−0.03
−0.02
−0.01
0.00
0.01
0.02
0.03
0.04
0.05

(f) TS7′

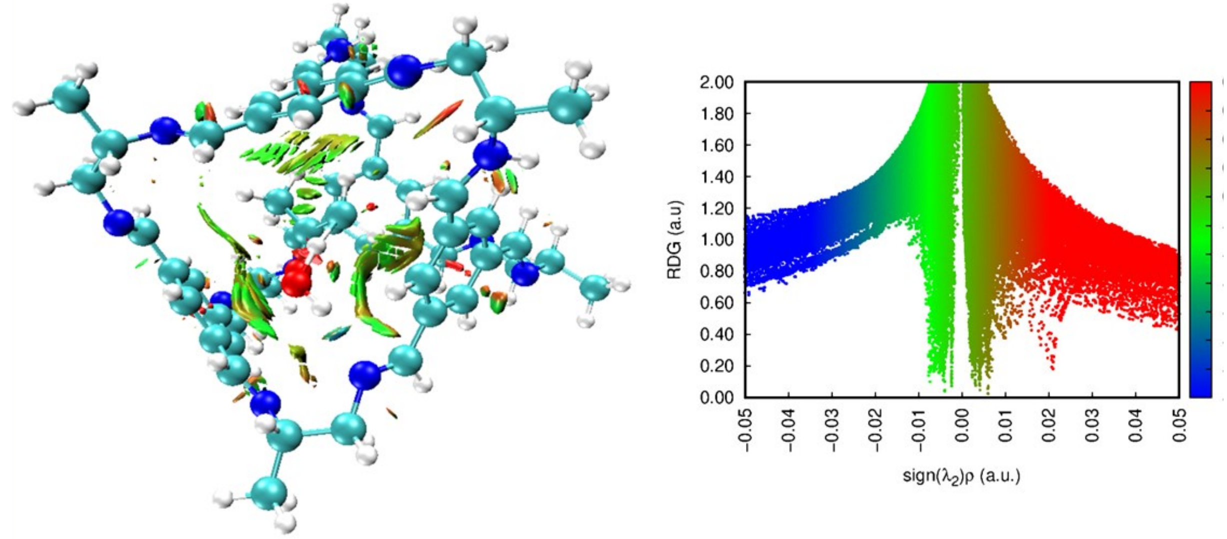
RDG (a.u)
sign(λ2)ρ (a.u.)
2.00
1.80
1.60
1.40
1.20
1.00
0.80
0.60
0.40
0.20
0.00
−0.05
−0.04
−0.03
−0.02
−0.01
0.00
0.01
0.02
0.03
0.04
0.05

(g) TS8′

(h) TS9′

Figure S1: 3D-isosurfaces and 2D-RDG graphs at M06-2X of transition states inside CC2 cage: where (a) Cheletropic reaction, (b) Dyotropic reaction, (c) Ring-expansion and contraction reaction, (d) 1,5-Sigmatropic shift reaction, (e) 6-π electron cyclization reaction, (f) Claisen rearrangement reaction, (g) Keto-enol tautomerism reaction and (h) [2+4] cycloaddition reaction.

Table S1: Energies of reactants, products and transition states (TS) with and without CC2 cage in a.u.

Organic Transformation	Without CC2 cage			With CC2 cage		
	Reactant	TS	Product	Reactant	TS	Product
Trimerization reaction	-231.87	-231.79	-232.14	-2981.55	-2981.47	-2981.81
Cheletropic reaction	-704.40	-704.37	-704.42	-3454.10	-3454.06	-3454.10
Dyotropic reaction	-998.91	-998.82	-998.91	-3748.58	-3748.50	-3748.59
Ring-expansion and contraction	-307.20	-307.16	-307.18	-3056.88	-3056.83	-3056.86
1,5-Sigmatropic shift	-195.19	-195.14	-195.20	-2944.91	-2944.81	-2944.89
6-π electron cyclization	-233.25	-233.22	-233.30	-2982.93	-2982.90	-2982.98
Claisen rearrangement	-270.40	-270.32	-270.37	-3020.08	-3019.99	-3020.05
[2+4] cycloaddition	-234.44	-234.41	-234.52	-2984.12	-2984.08	-2984.20

Cartesian coordinates for all computed structures

1. **Trimerization Reaction**

Without CC2 cage

Reactant

0 1

C	-1.83745000	0.88200100	0.00127200
C	-2.88744200	0.29340500	-0.00079800
C	0.15479100	-2.03219000	0.00004100
C	1.18970800	-2.64689600	0.00111600
C	1.68247700	1.15003500	-0.00302500
C	1.69792100	2.35365000	0.00130300
H	-0.89401000	1.39049000	0.00310000
H	-3.82580800	-0.21573900	-0.00259900
H	-0.75754800	-1.46975800	-0.00036100
H	2.10004300	-3.20461100	0.00217000
H	1.65100400	0.07872000	-0.00687500
H	1.72628700	3.42085800	0.00510700

TS

0 1

C	0.75841800	1.61722300	-0.00047500
C	-0.46218000	1.72541900	0.00034200
C	-1.78000700	-0.15179700	0.00015700
C	-1.26360300	-1.26306100	-0.00050100
C	1.02177900	-1.46548800	0.00041600
C	1.72553600	-0.46233500	0.00006000

H	1.75883500	1.98753100	-0.00134600
H	-1.38189400	2.26589700	0.00108900
H	-2.60079600	0.52957200	0.00060500
H	-1.27122400	-2.32978100	-0.00131400
H	0.84199200	-2.51696700	0.00104800
H	2.65342200	0.06398000	-0.00008400

Product

0 1

C	-0.59734600	1.25840200	-0.00036000
C	0.79118300	1.14642100	0.00039500
C	1.38853000	-0.11187800	0.00026100
C	0.59733900	-1.25837300	-0.00029400
C	-0.79120600	-1.14650800	0.00010400
C	-1.38852900	0.11193100	-0.00010600
H	-1.06279600	2.23931200	-0.00059000
H	1.40789100	2.04024900	0.00035800
H	2.47080200	-0.19910600	-0.00016500
H	1.06308700	-2.23944700	-0.00087100
H	-1.40784100	-2.04009800	0.00076300
H	-2.47097100	0.19912600	0.00051600

With CC2 cage

Reactant

0 1

N	-1.42660300	4.87470800	-2.25383800
N	4.85574300	-0.04139300	-3.29898900

N	2.00424500	4.50984500	3.14075400
N	3.73665600	-4.43521300	1.26255100
N	2.30692500	-3.84826900	3.71928800
N	4.68015400	2.74267500	-2.45670500
N	-0.26583800	-3.95205400	-3.73261000
C	0.93143700	4.46552300	0.48928900
H	0.29262800	4.81389400	1.29639700
C	3.55511300	-4.58043300	3.66981900
H	4.43559000	-3.92310600	3.75811600
N	-3.60640400	-1.37908900	4.23723100
C	3.46492100	3.05565200	-2.63688100
H	2.98927000	3.00009800	-3.62847500
N	1.54951600	2.36016300	5.04401200
C	2.59705500	3.53122100	-1.54299300
C	2.68820600	4.00465800	2.20003100
H	3.68056700	3.55951100	2.37317200
C	2.21182200	3.99487400	0.80301500
C	-0.88441400	4.94181600	-1.10685700
H	-1.41873500	5.36854300	-0.24398900
C	-2.57411500	-2.01992100	3.87534700
H	-2.62399500	-3.06388400	3.52947500
C	3.03713900	3.52544300	-0.21319100
H	4.03587300	3.15363700	0.00159200
C	1.32452700	4.00409300	-1.84212700
H	0.97769500	4.03019000	-2.87142300

C	0.48002000	4.46369200	-0.82471400
C	1.78003600	-3.74572000	-1.72549700
H	1.05636500	-4.55124700	-1.63178600
C	4.72854400	-0.70958500	-2.22944000
H	5.42566600	-0.59381000	-1.38479200
C	1.70595400	-2.89685000	-2.83868200
C	-0.10673400	-2.24570000	3.76936800
H	-0.21738300	-3.30072300	3.53257900
C	0.65390500	-3.08503200	-3.85658800
H	0.72561200	-2.43766200	-4.74420900
C	-1.22014000	-1.43220100	3.94730200
C	3.71472900	-2.55339800	-0.93056400
H	4.49021200	-2.43862600	-0.17743800
C	2.37937200	-2.58626200	3.81448200
H	3.34651300	-2.06161800	3.86235000
C	-1.04354600	-0.07704800	4.26268500
H	-1.92628600	0.54148400	4.40674100
C	5.42820900	2.29490200	-3.61174900
H	4.82661100	2.29885200	-4.53537300
C	0.41050000	1.87114000	4.77791300
H	-0.51099900	2.47306300	4.82622100
C	2.65482600	-1.89275900	-2.99582900
H	2.63165000	-1.22870700	-3.85630400
C	2.56436400	4.48146100	4.47372900
H	3.55244000	3.99404600	4.51103500

C	3.66548100	-5.37661900	2.36628200
H	2.77271600	-6.01865300	2.28623500
C	-1.19004400	-4.11309300	-4.83852200
H	-1.08009500	-3.32043600	-5.59573300
C	1.18604100	-1.72349800	3.91861400
N	-3.90054400	3.27020700	-2.22345500
C	2.80909200	-4.47516400	0.39966700
H	1.97842100	-5.19451200	0.47099500
C	2.76780800	-3.57271300	-0.76641600
C	5.97372900	0.88300900	-3.38355400
H	6.55380300	0.89353800	-2.44590000
C	0.23474000	0.45168300	4.40565700
C	1.34970700	-0.37915500	4.22710400
H	2.34053900	0.04857200	4.35595700
C	3.67162800	-1.72385200	-2.04464400
C	1.61836000	3.75691300	5.43525800
H	0.62993000	4.24040500	5.36760900
C	-2.75763500	5.44866200	-2.39282900
H	-3.17930500	5.70014900	-1.40591500
N	-5.43663200	-1.43475600	1.93696900
C	-3.68977600	4.43825000	-3.05785600
H	-3.29175100	4.17509400	-4.05199600
N	-3.00140300	-2.84691200	-3.83440400
C	-4.74317200	-0.35557800	-0.09475600
C	-3.87077100	2.13750700	-2.79806100

H	-3.64957800	2.03865700	-3.87150200
C	-4.87637400	-2.07309000	4.20051000
H	-4.78387800	-3.11067500	3.84037800
C	-4.57702800	0.85226900	-0.76478100
H	-4.80465000	1.79476800	-0.27230700
C	-3.96248200	-1.54274600	-2.05526800
C	-5.88244400	-1.33365800	3.31437300
H	-5.92184200	-0.28359100	3.64841500
C	-3.80734500	-0.32542600	-2.72410000
H	-3.41109800	-0.32953500	-3.73600200
C	-4.43883900	-1.55410200	-0.74970100
H	-4.54640800	-2.48667100	-0.20201400
C	-4.10401400	0.87180100	-2.08244600
C	-2.64229100	-4.15074800	-4.36384100
H	-2.72960900	-4.93332000	-3.59106500
C	-3.54636100	-2.80654700	-2.68987900
H	-3.71871800	-3.71750300	-2.09403200
C	-5.20309200	-0.35962800	1.30720600
H	-5.32632500	0.63017900	1.77379500
C	-2.65188900	6.71358900	-3.24389800
H	-2.21869200	6.47519600	-4.22008400
H	-3.63846100	7.16211200	-3.39562500
H	-2.00623300	7.44821900	-2.75588700
C	-3.57304500	-4.47592700	-5.53153300
H	-3.50064200	-3.69776700	-6.29737200

H	-3.31179000	-5.43938200	-5.97994200
H	-4.61025500	-4.52036600	-5.18945500
C	4.92618700	-6.23719100	2.36684200
H	5.81414900	-5.60330300	2.44907700
H	4.91414200	-6.94046600	3.20512700
H	5.00013500	-6.80612500	1.43643600
C	2.14036000	3.83892600	6.86801000
H	3.12056200	3.35813200	6.93941400
H	2.23350200	4.88201500	7.18529900
H	1.45952600	3.32624800	7.55243600
C	6.87177200	0.45962500	-4.54366700
H	6.30097300	0.45070300	-5.47702800
H	7.71517800	1.14861500	-4.65066700
H	7.26345900	-0.54691400	-4.37538900
H	6.28062300	2.97169500	-3.74948800
H	-4.66239800	4.92694100	-3.20211100
H	3.56600600	-5.28970300	4.50652400
H	-0.96539200	-5.07621600	-5.31530700
H	2.67460100	5.51717400	4.81849700
H	-5.27290700	-2.09492300	5.22336500
C	-7.26640000	-1.96853600	3.43076800
H	-7.62576400	-1.92714900	4.46351500
H	-7.98130900	-1.44360900	2.79188100
H	-7.22851600	-3.01408300	3.11036900
C	-0.79409400	1.73985400	-3.02428000

C	-0.67761200	0.55134000	-2.85995700
C	-1.03230300	-2.52735600	-0.78998000
C	-1.15699900	-1.50495400	-0.16416000
C	-1.15637000	1.96002400	1.27902000
C	-1.87092200	2.27543000	0.36142300
H	-0.92942300	2.79973800	-3.13245100
H	-0.59982300	-0.49973500	-2.66971200
H	-0.93954100	-3.40357700	-1.39914000
H	-1.28349500	-0.57775200	0.35855600
H	-0.49996500	1.67283500	2.07240500
H	-2.49238400	2.58847000	-0.45651300

TS

0 1

N	0.45635100	-2.42238900	-4.94992700
N	-5.18547300	2.19711200	-1.58038800
N	-2.11522200	-5.50245300	0.03798100
N	-2.73138100	2.75092200	4.32777000
N	-1.13196300	0.70497500	5.63127700
N	-5.25156200	-0.53242700	-2.62359300
N	0.24771500	5.53551000	-0.59701400
C	-1.42882000	-3.83704100	-2.18295000
H	-0.75101800	-4.64320900	-1.91519500
C	-2.25161600	1.37433400	6.25755400
H	-3.20758000	0.85254200	6.08845400
N	4.39230400	-1.68648500	3.42902700

C	-4.12784500	-0.69083600	-3.18902400
H	-3.79438000	-0.04193100	-4.01392800
N	-1.07103300	-4.97707500	2.70658900
C	-3.19282000	-1.76824600	-2.81370600
C	-2.82686400	-4.48563000	-0.22102800
H	-3.68834700	-4.20064600	0.40299900
C	-2.55937100	-3.62020700	-1.38593900
C	0.04779700	-3.24393000	-4.07105300
H	0.60473400	-4.16588500	-3.83987600
C	3.41696800	-0.94750200	3.75839900
H	3.56093900	0.07283300	4.14616400
C	-3.43472500	-2.58785100	-1.70345100
H	-4.32241400	-2.40185100	-1.10413500
C	-2.06305500	-1.98659600	-3.59205000
H	-1.85677300	-1.35983700	-4.45607500
C	-1.17316000	-3.02151900	-3.27767900
C	-1.36247300	4.01785000	1.20781000
H	-0.48952800	4.53433200	1.59840800
C	-4.79677000	2.04957000	-0.38301900
H	-5.35439400	1.43237800	0.33845600
C	-1.63549800	4.10897100	-0.16492800
C	1.01010000	-0.64460500	4.26313600
H	1.23417600	0.30875000	4.73497600
C	-0.75544000	4.90228400	-1.04650400
H	-1.02888600	4.92096900	-2.11402600

C	2.01686300	-1.41410800	3.69032100
C	-3.31198600	2.63410200	1.50675600
H	-3.95740400	2.07261700	2.17729600
C	-1.38217600	-0.31940700	4.92817800
H	-2.40549800	-0.70463500	4.79967100
C	1.69583200	-2.64840500	3.10436100
H	2.49811300	-3.24196300	2.67271100
C	-6.09055000	0.54434200	-3.10490000
H	-5.63194500	1.10020500	-3.93909900
C	0.06568700	-4.43931200	2.54788300
H	0.88631400	-4.94721000	2.01793800
C	-2.74737400	3.45980800	-0.68799700
H	-2.98994800	3.52266200	-1.74651600
C	-2.45341100	-6.28330700	1.20666300
H	-3.34030300	-5.89832400	1.73629300
C	-2.37508300	2.80842000	5.73439200
H	-1.40221800	3.30609600	5.88088900
C	1.03369300	6.31618600	-1.52787000
H	0.73120200	6.15778500	-2.57577900
C	-0.31711300	-1.09707200	4.26421700
N	3.05834700	-1.19774200	-4.42194100
C	-1.92264200	3.25950100	3.49435600
H	-0.97862300	3.72985600	3.81040000
C	-2.19766400	3.29250100	2.04501200
C	-6.41224600	1.52683600	-1.97588800

H	-6.83914300	0.94976800	-1.13896500
C	0.38253400	-3.10667600	3.10372200
C	-0.62437700	-2.31933000	3.68030700
H	-1.64285800	-2.69856000	3.67815100
C	-3.59124600	2.71461300	0.14885300
C	-1.27250700	-6.31621700	2.18102500
H	-0.38585700	-6.66617900	1.62731700
C	1.65851600	-2.77647600	-5.69109900
H	2.19398400	-3.60313800	-5.19628600
N	5.75526400	-0.14814700	1.36387700
C	2.59182600	-1.56817100	-5.74375900
H	2.07541100	-0.73831000	-6.25396800
N	2.73649100	4.61493500	-1.78020000
C	4.54626400	0.28586900	-0.67393500
C	2.98471100	0.03082400	-4.10650000
H	2.55138100	0.77646300	-4.79155300
C	5.72413200	-1.13746000	3.57209700
H	5.72026100	-0.12113400	3.99842100
C	4.12074800	-0.24272200	-1.88709500
H	4.28958900	-1.28964300	-2.12719200
C	3.64086500	2.44163200	-1.29870600
C	6.44016700	-1.10079700	2.21890300
H	6.40816100	-2.11736600	1.79276600
C	3.22139100	1.90021500	-2.51905100
H	2.68666800	2.53959300	-3.21625900

C	4.30500900	1.63471500	-0.38318800
H	4.63734600	2.02806500	0.57418200
C	3.45833800	0.56514700	-2.81823000
C	2.52178300	5.99364300	-1.38163900
H	2.80261800	6.15437000	-0.32748100
C	3.35018700	3.85027000	-0.97432800
H	3.69551100	4.19936000	0.01177400
C	5.24884300	-0.58472400	0.28604600
H	5.31733500	-1.64622200	0.00054300
C	1.25290600	-3.19844600	-7.10283000
H	0.69599600	-2.39270700	-7.59062200
H	2.13595000	-3.43213100	-7.70541300
H	0.61112600	-4.08272600	-7.06737200
C	3.35661500	6.90100800	-2.28345000
H	3.09000100	6.73689100	-3.33185900
H	3.18927900	7.95385400	-2.03600200
H	4.42058500	6.68010200	-2.16512400
C	-3.46491900	3.56527800	6.48918500
H	-4.43075200	3.06992600	6.35195100
H	-3.23665700	3.60715600	7.55856100
H	-3.55078100	4.58728600	6.11123400
C	-1.56239000	-7.25842300	3.34734800
H	-2.44363400	-6.91583100	3.89788800
H	-1.74388600	-8.27497700	2.98541300
H	-0.71670600	-7.28096200	4.03956100

C	-7.41518500	2.57734800	-2.44665400
H	-6.99829900	3.14741000	-3.28234200
H	-8.34713400	2.10408100	-2.77059400
H	-7.64263900	3.27708100	-1.63828800
H	-7.03396200	0.10803500	-3.45610900
H	3.46789200	-1.84545400	-6.34439800
H	-2.06300600	1.41609500	7.33710900
H	0.88781300	7.37588800	-1.28195000
H	-2.65961300	-7.31117700	0.88367300
H	6.29001800	-1.79490200	4.24344600
C	7.89057800	-0.65657700	2.39193000
H	8.43039700	-1.34291500	3.05158500
H	8.39861500	-0.63138900	1.42446000
H	7.92686100	0.34942900	2.82066800
C	0.19034600	0.19661200	-2.75444300
C	-0.13101400	1.22187600	-2.15412900
C	0.66544100	1.56639900	-0.15716500
C	0.80890600	0.59070900	0.57930300
C	0.97187600	-1.45549100	-0.36155900
C	1.19327100	-1.46368200	-1.56950100
H	0.24935400	-0.48176100	-3.58463400
H	-0.66381900	2.14444800	-2.05221700
H	0.72006700	2.61204400	-0.38842400
H	1.01731600	0.10215200	1.50814000
H	0.88092700	-1.87101000	0.61932800

H	1.58208600	-1.80426000	-2.50840400

Product

0 1

N	-1.75040000	5.33791900	-0.46405800
N	4.85875700	1.38407900	-2.76703800
N	1.45241400	3.12951800	4.64497000
N	3.79597600	-4.44167000	-0.24186400
N	2.29152600	-4.92233300	2.19744400
N	4.45511700	3.66634800	-1.01367700
N	0.20024500	-2.34363100	-5.12216700
C	0.47749500	4.03405700	2.10069600
H	-0.21705200	4.02163900	2.93668000
C	3.57822500	-5.52156900	1.91467200
H	4.42024900	-4.91234300	2.28182000
N	-3.77910800	-3.01818100	3.17309800
C	3.24390600	4.01730800	-1.14458600
H	2.83501300	4.34838000	-2.11254100
N	1.08908300	0.38136800	5.59767200
C	2.29431400	4.02157400	-0.01610000
C	2.18526000	3.01681400	3.61657600
H	3.18439400	2.55564500	3.65934300
C	1.76751100	3.52173100	2.29464200
C	-1.28834500	4.99875100	0.66824600
H	-1.90862900	5.02795700	1.57839000
C	-2.68773600	-3.50045200	2.74510300

H	-2.65695000	-4.38783000	2.09394900
C	2.66540500	3.51007700	1.23533700
H	3.66975000	3.11259100	1.35651900
C	1.01514800	4.53090100	-0.19575500
H	0.69991900	4.92250200	-1.16008000
C	0.09540600	4.52864000	0.86138100
C	1.91201900	-2.73374900	-2.84662800
H	1.19309400	-3.51501400	-3.07918500
C	4.75112700	0.35005000	-2.04169200
H	5.41745900	0.16678900	-1.18448900
C	1.87852100	-1.54990600	-3.59602400
C	-0.20901700	-3.59188300	2.74644800
H	-0.24429800	-4.51474900	2.17307500
C	0.91960300	-1.38624600	-4.70710300
H	0.89490100	-0.38977500	-5.17519100
C	-1.37595800	-2.93130300	3.11361500
C	3.77637500	-1.88195300	-1.57371200
H	4.51158600	-2.03603500	-0.78794000
C	2.28910300	-3.78991500	2.76727200
H	3.22208700	-3.27786800	3.05000000
C	-1.29387800	-1.74816800	3.86210000
H	-2.21765500	-1.25387800	4.15297000
C	5.29267800	3.70057500	-2.19247100
H	4.74413400	4.01561500	-3.09543900
C	0.00605100	-0.02068600	5.07634100

H	-0.95227300	0.49388500	5.24767800
C	2.79415500	-0.54124400	-3.32231700
H	2.79906000	0.38166800	-3.89720400
C	1.96781300	2.62049200	5.89672000
H	2.98087000	2.19647600	5.80083600
C	3.74794100	-5.73997400	0.40821200
H	2.88765000	-6.33176600	0.05435400
C	-0.67014000	-2.09120000	-6.25033300
H	-0.59308300	-1.05739600	-6.62481400
C	1.04293300	-3.08222700	3.12090000
N	-4.05891200	3.68416100	-1.26131000
C	2.89436400	-4.17800200	-1.09309000
H	2.09578700	-4.89568600	-1.33704900
C	2.85652500	-2.90622000	-1.84181800
C	5.91713300	2.32622100	-2.44506600
H	6.45259700	2.02555600	-1.52942500
C	-0.05855800	-1.23497400	4.23796200
C	1.11164300	-1.90697000	3.85944800
H	2.06890400	-1.49519600	4.16825800
C	3.74980000	-0.70216700	-2.30865500
C	1.03545400	1.54672300	6.46318000
H	0.01794100	1.96933500	6.50277800
C	-3.11887100	5.82919500	-0.50383700
H	-3.62943000	5.65610000	0.45792700
N	-5.32047900	-2.28931100	0.81822900

C	-3.89559600	5.08564100	-1.59004700
H	-3.38990100	5.23534700	-2.55815000
N	-2.56083300	-1.38129900	-4.91647700
C	-4.53563100	-0.51410400	-0.60522900
C	-3.74354300	2.83822800	-2.15296400
H	-3.33322000	3.14906500	-3.12703400
C	-5.00388600	-3.67871500	2.77482900
H	-4.82412200	-4.54197800	2.11339900
C	-4.46882000	0.85903700	-0.80018900
H	-4.84994900	1.54903000	-0.05099800
C	-3.46021000	-0.86748800	-2.74653600
C	-5.94201400	-2.69965200	2.06464100
H	-6.10350600	-1.83803400	2.73358800
C	-3.39354900	0.52023200	-2.93065600
H	-2.93713700	0.90198100	-3.84025800
C	-4.02205500	-1.37709900	-1.58337400
H	-4.08023500	-2.44890500	-1.41228100
C	-3.88575900	1.38353000	-1.96161800
C	-2.12875600	-2.37491000	-5.88264800
H	-2.17938200	-3.39285400	-5.46183300
C	-2.96126700	-1.78954300	-3.78494900
H	-2.97901700	-2.85909400	-3.52346300
C	-5.14013300	-1.04856200	0.62972000
H	-5.43467900	-0.29708600	1.37947900
C	-3.08579800	7.32696500	-0.80609000

H	-2.57089900	7.50802100	-1.75448700
H	-4.10051000	7.73144700	-0.87190600
H	-2.54739200	7.86236200	-0.01970300
C	-3.02222500	-2.27855400	-7.11725200
H	-2.96826200	-1.27197900	-7.54278100
H	-2.71149800	-3.00193000	-7.87728200
H	-4.06319400	-2.47878800	-6.85067700
C	5.04720800	-6.48610400	0.11545200
H	5.90316400	-5.90402900	0.46980900
H	5.05262700	-7.46177900	0.61086100
H	5.16384700	-6.64126400	-0.96022400
C	1.48036300	1.13211900	7.86406700
H	2.49304800	0.71920100	7.83040000
H	1.47033000	1.99101200	8.54199900
H	0.81369800	0.36411300	8.26476200
C	6.89166600	2.38600600	-3.61930500
H	6.36405800	2.68114300	-4.53124200
H	7.68965700	3.10844600	-3.42238700
H	7.34392500	1.40569900	-3.79055400
H	6.10539800	4.41573200	-2.01360400
H	-4.89628400	5.53248300	-1.65339900
H	3.61510400	-6.49998800	2.40904100
H	-0.38050800	-2.77575300	-7.05727400
H	2.00550700	3.45210700	6.61128300
H	-5.50564800	-4.03590700	3.68281600

C	-7.27810400	-3.36977300	1.75164800
H	-7.75984200	-3.72032500	2.66958100
H	-7.94960300	-2.66618500	1.25275200
H	-7.12337600	-4.22317100	1.08464400
C	-0.70644500	1.90799000	-0.86134500
C	0.34662200	1.06115800	-1.19541000
C	0.57983800	-0.08957200	-0.44408600
C	-0.23936600	-0.39027300	0.64170900
C	-1.29032500	0.46121400	0.97862800
C	-1.52007200	1.61199400	0.23003800
H	-0.89599700	2.81352500	-1.43497000
H	0.99490200	1.29748600	-2.03577600
H	1.40107100	-0.75046600	-0.70677900
H	-0.06066100	-1.28802800	1.22651600
H	-1.93288300	0.21778300	1.82144300
H	-2.34162500	2.28178800	0.47753300

2. Cheletropic reaction

Without CC2 cage

Reactant

0 1

C	-1.02839200	-1.34356700	-1.00209300
C	-1.53071500	-1.07743900	0.20832500
C	-2.21347500	0.17475900	0.57456400
C	-1.95425800	1.36563300	0.02868600
S	1.34873300	0.33853400	0.33483500

H	-1.13510900	-0.64422400	-1.82715000
H	-1.44190600	-1.82853100	0.99201400
H	-2.96066400	0.10331100	1.36277600
H	-1.18473200	1.49294800	-0.72926500
O	2.16038900	-0.83029100	0.59584800
O	1.40336300	0.92557900	-0.98771400
H	-0.50427400	-2.27197300	-1.20463800
H	-2.50202100	2.25330900	0.32693900

TS

0 1

C	0.89016100	1.39887100	-0.55493400
C	1.82358300	0.69969100	0.20420800
C	1.82362100	-0.69961600	0.20432000
C	0.89024300	-1.39896700	-0.55470800
S	-0.90171100	-0.00000500	0.05724400
H	0.63545000	1.08337100	-1.56424100
H	2.35241800	1.21846200	0.99782900
H	2.35247700	-1.21822800	0.99803200
H	0.63549100	-1.08362100	-1.56405300
O	-1.15168000	0.00016000	1.48748000
O	-2.04271600	-0.00012300	-0.84445400
H	0.72047100	2.45426900	-0.36069400
H	0.72059100	-2.45433900	-0.36029700

Product

0 1

C	-0.48846700	-1.34701900	-0.17562000
C	-1.81478200	-0.66674300	0.00011000
C	-1.81478300	0.66674300	0.00011600
C	-0.48846900	1.34702000	-0.17560700
S	0.71844100	0.00000000	0.02455500
H	-0.34869500	-1.78033100	-1.17013700
H	-2.71570300	-1.25817800	0.12401800
H	-2.71570400	1.25817600	0.12403000
H	-0.34869600	1.78034000	-1.17012200
O	1.18040900	-0.00001000	1.40809400
O	1.67108300	0.00000900	-1.07949600
H	-0.26959300	-2.09771300	0.58826700
H	-0.26959400	2.09770800	0.58828500

With cage

Reactant

0 1

N	-4.10556600	4.21829600	-0.74437200
N	3.03337000	2.23344900	-4.14971000
N	0.69999900	4.43215100	3.53321700
N	4.94626500	-2.82120000	-0.67613800
N	4.28812100	-3.13800700	2.12441000
N	2.00420300	4.54164200	-2.72608400
N	-0.07034700	-3.34024200	-4.64940500
C	-1.07253200	4.23085500	1.26949600
H	-1.51839800	4.20556400	2.26060700

C	5.62208200	-3.31879600	1.59248200
H	6.21355100	-2.38936900	1.60363400
N	-1.69133900	-3.29793800	4.43672000
C	0.75779500	4.36157300	-2.58292200
H	0.07805800	4.26189500	-3.44444600
N	1.75770000	2.06914600	4.93266400
C	0.12535800	4.29874900	-1.25048700
C	1.17886500	4.29064100	2.36832500
H	2.25957900	4.19940800	2.18512100
C	0.32398200	4.26590900	1.16487800
C	-3.33229900	4.15676000	0.25783000
H	-3.71517600	4.04884700	1.28455600
C	-0.61988500	-3.44675700	3.77559500
H	-0.38024200	-4.38790200	3.25552600
C	0.91418100	4.29245300	-0.09193000
H	1.99562600	4.31424200	-0.19932600
C	-1.25974500	4.26050900	-1.13773800
H	-1.89618900	4.25272600	-2.01908300
C	-1.86453200	4.22394300	0.12672900
C	2.05909200	-2.56044700	-2.87405700
H	1.69143200	-3.58118900	-2.80312700
C	3.48420000	1.44416400	-3.26572600
H	4.29401100	1.73313300	-2.57748700
C	1.41428000	-1.67576700	-3.75029600
C	1.63756100	-2.65874100	3.11543100

H	1.86280000	-3.64156100	2.70882800
C	0.28296500	-2.12459500	-4.58449400
H	-0.22034600	-1.33834100	-5.16923900
C	0.40049400	-2.38351800	3.68422900
C	3.61932200	-0.82268200	-2.26307100
H	4.48466900	-0.51631900	-1.68169200
C	3.97117800	-1.97447200	2.51609500
H	4.67053200	-1.12772800	2.46685700
C	0.14482300	-1.10401900	4.19800100
H	-0.82894100	-0.90768700	4.63951600
C	2.53046500	4.59964400	-4.07148600
H	1.75574300	4.44302600	-4.83997500
C	0.85176500	1.19918000	4.76588600
H	-0.18571200	1.36566500	5.09542900
C	1.86791700	-0.36758400	-3.85940700
H	1.38511200	0.33769200	-4.53132100
C	1.63786600	4.47238500	4.63482300
H	2.68781300	4.43704100	4.30207800
C	5.55253200	-3.84530000	0.15594000
H	4.94412300	-4.76495700	0.16368200
C	-1.18685000	-3.66391300	-5.51047900
H	-1.55987900	-2.78964200	-6.06845400
C	2.63783400	-1.67547600	3.07972900
N	-5.53357100	1.67571800	-0.66721100
C	3.84973400	-3.09886700	-1.24699100

H	3.36131500	-4.07893400	-1.12944200
C	3.15837200	-2.14154600	-2.13384500
C	3.63188600	3.55264200	-4.25389100
H	4.38514800	3.71337800	-3.46529300
C	1.12537700	-0.12138000	4.16459900
C	2.37847400	-0.41483900	3.60715300
H	3.13545200	0.36637900	3.58970100
C	2.97474400	0.06578100	-3.11683000
C	1.39066600	3.30777500	5.59742500
H	0.32185800	3.31514500	5.86834200
C	-5.53378000	4.12700500	-0.50296400
H	-5.75203500	3.99799500	0.57008500
N	-3.81689300	-3.65838300	2.52233200
C	-6.05997300	2.89498200	-1.24470700
H	-5.81268100	2.99818500	-2.31389000
N	-2.82762700	-3.21794300	-3.78928200
C	-3.99376800	-2.05536200	0.74106000
C	-4.82483800	0.93668000	-1.41526200
H	-4.60378300	1.20624600	-2.45980000
C	-2.61583900	-4.40970000	4.47799100
H	-2.25695900	-5.28218400	3.90786300
C	-4.41266000	-0.78535800	0.36173300
H	-4.84781300	-0.09941500	1.08449100
C	-3.33543200	-2.51872000	-1.54268100
C	-3.98034100	-3.98569200	3.92680500

H	-4.32243500	-3.11120800	4.50472000
C	-3.75195000	-1.23333600	-1.91347200
H	-3.65928700	-0.93593300	-2.95500700
C	-3.45934400	-2.92286200	-0.21969000
H	-3.14582800	-3.91537800	0.09418900
C	-4.29998500	-0.37036200	-0.97253800
C	-2.33732500	-4.24890800	-4.68716100
H	-1.94762000	-5.11466800	-4.12659700
C	-2.81252000	-3.46567100	-2.54582700
H	-2.43372300	-4.42007000	-2.14718800
C	-4.11581000	-2.48443400	2.14727900
H	-4.48966500	-1.72448400	2.85130700
C	-6.20696900	5.39501900	-1.02199900
H	-5.99614000	5.52305400	-2.08793800
H	-7.29057000	5.34388400	-0.87871600
H	-5.82682300	6.27260800	-0.49277800
C	-3.47970200	-4.69278200	-5.59782600
H	-3.87235800	-3.83595700	-6.15337600
H	-3.13467300	-5.44894400	-6.30963700
H	-4.29531100	-5.11728800	-5.00677200
C	6.95030000	-4.15016000	-0.37604800
H	7.55753900	-3.24019100	-0.37954000
H	7.44401500	-4.90353800	0.24537500
H	6.89533800	-4.52615100	-1.40094000
C	2.24618300	3.45744800	6.85326400

H	3.30719300	3.46686200	6.58628000
H	2.00385500	4.38748200	7.37633400
H	2.07487900	2.61972600	7.53425900
C	4.28191000	3.68761500	-5.62896800
H	3.53741800	3.52715300	-6.41451400
H	4.71902600	4.68304700	-5.75315800
H	5.07221300	2.94267300	-5.75242600
H	2.97321400	5.59219500	-4.22135500
H	-7.15175100	2.87153100	-1.14147000
H	6.13652600	-4.06840600	2.20649600
H	-0.85613400	-4.42252400	-6.23008100
H	1.47715400	5.40911600	5.18247400
H	-2.74521000	-4.70726100	5.52581300
C	-4.99117500	-5.12224000	4.05704200
H	-5.10943200	-5.41644300	5.10435800
H	-5.96543400	-4.81228700	3.67035600
H	-4.65688600	-5.99013800	3.48082100
C	-0.49360300	-1.52320200	0.25221600
C	-0.96247600	-0.28370500	0.42580200
C	-0.77324100	0.81630600	-0.53337800
C	-0.69643300	0.65595800	-1.85763500
S	2.23322700	0.50714900	0.51530900
H	0.08940300	-1.79105400	-0.62818200
H	-1.49596500	-0.04267100	1.34437900
H	-0.68495600	1.81437600	-0.10930300

H	-0.83014100	-0.32084600	-2.31903900
O	1.58891200	1.53164700	1.31302400
O	3.68291400	0.45295200	0.58613400
H	-0.68490500	-2.30477000	0.98190500
H	-0.51282000	1.50047600	-2.51718400

TS

0 1

N	-3.95550900	4.25597800	1.40865700
N	4.15423900	3.94477000	-0.05410800
N	-0.99432300	0.31562800	5.52908400
N	5.06291700	-2.37188300	-1.06354000
N	3.48356600	-4.53044900	0.20923700
N	2.43905600	4.57710000	2.19886800
N	1.85386800	1.14018500	-5.34021100
C	-1.82186000	2.18427500	3.51044300
H	-2.58241500	1.54240000	3.94776100
C	4.92880800	-4.47948500	0.13891100
H	5.37963400	-3.96125900	1.00029300
N	-2.91462700	-5.17788600	-0.21533500
C	1.21863500	4.69037800	1.87301700
H	0.87230500	5.46066500	1.16545200
N	-0.32793400	-2.51836700	4.99033600
C	0.17025800	3.81165100	2.43022700
C	-0.11838300	0.93925700	4.85853600
H	0.95874200	0.77226400	4.99603100

C	-0.48473600	1.97138500	3.86656900
C	-3.57552500	3.36481100	2.22643600
H	-4.28255900	2.65441200	2.68258000
C	-1.65964600	-5.11499200	-0.38322400
H	-1.17480400	-5.46475200	-1.30822400
C	0.50472200	2.77162100	3.30991700
H	1.55105400	2.61468500	3.55921000
C	-1.16182800	4.02245300	2.09077700
H	-1.44877700	4.81614600	1.40517700
C	-2.16414000	3.19630200	2.62050200
C	3.18326700	0.00707400	-3.06542600
H	2.83835500	-0.66654700	-3.84599300
C	4.44241200	2.70981000	-0.01755000
H	4.99009200	2.26023800	0.82494500
C	2.90512000	1.37674200	-3.19417500
C	0.61529500	-4.55629200	0.44744100
H	1.05491500	-4.99505800	-0.44502600
C	2.15619900	1.88399000	-4.35968300
H	1.88316700	2.95025400	-4.32757900
C	-0.76137600	-4.55322300	0.64426000
C	4.33476700	0.42780800	-0.98632400
H	4.87283900	0.03211000	-0.12978400
C	2.93379800	-3.97280300	1.20637000
H	3.51205100	-3.45959500	1.98675500
C	-1.29373700	-3.97012400	1.80412100

H	-2.37262700	-3.97287700	1.93664200
C	3.38208100	5.51210000	1.62317900
H	2.93246700	6.13629800	0.83362400
C	-1.03876700	-2.84849300	3.99544400
H	-2.13753600	-2.77218300	4.01145100
C	3.33878800	2.25656000	-2.21191600
H	3.12371300	3.32016900	-2.27686500
C	-0.52065700	-0.63754500	6.51056000
H	0.57824800	-0.65768700	6.58835100
C	5.38504500	-3.78654700	-1.14756500
H	4.85936000	-4.26661800	-1.98972700
C	1.10146700	1.73032500	-6.42492000
H	0.86190300	2.79188100	-6.24967900
C	1.46850500	-3.97951900	1.39989900
N	-5.09053600	2.72328800	-0.77903100
C	4.24476500	-1.90533800	-1.91036700
H	3.77439500	-2.53971200	-2.67841600
C	3.90341400	-0.46947600	-1.97662600
C	4.59558600	4.77661600	1.05164100
H	5.03353400	4.16662600	1.85860000
C	-0.45691000	-3.40866300	2.75937700
C	0.93011000	-3.41534500	2.55155500
H	1.56956700	-2.95468500	3.30009400
C	4.04726200	1.78299400	-1.09673700
C	-1.01203400	-2.04920600	6.18277800

H	-2.10269700	-2.00070200	6.02683900
C	-5.36904000	4.30493900	1.07979600
H	-5.92949200	3.51553100	1.60741700
N	-4.08666000	-3.47930500	-2.21952500
C	-5.51566600	4.06226400	-0.42536400
H	-4.95058500	4.84040200	-0.96391300
N	-1.03484200	1.10543700	-5.46306200
C	-3.71860300	-1.10348700	-2.13030200
C	-4.12834700	2.61959800	-1.59840600
H	-3.62893900	3.50474500	-2.02267700
C	-3.71726200	-5.69295900	-1.30149500
H	-3.11651000	-5.96182500	-2.18581400
C	-4.14668800	0.10898800	-1.60073500
H	-4.89882000	0.14374800	-0.81603100
C	-2.26499200	0.08249100	-3.66533300
C	-4.76865100	-4.65547200	-1.70682600
H	-5.36320400	-4.41139500	-0.81103900
C	-2.69443300	1.29822800	-3.11665500
H	-2.30003500	2.22254900	-3.53091700
C	-2.76789700	-1.11036300	-3.16104900
H	-2.46189500	-2.06716700	-3.57645400
C	-3.63382500	1.31867600	-2.09233900
C	-0.19934400	0.95290500	-6.64199800
H	0.06613800	-0.10429600	-6.80765400
C	-1.35543300	0.05558700	-4.82876300

H	-1.00919100	-0.94229900	-5.14018800
C	-4.31326100	-2.36861600	-1.65142700
H	-4.99225900	-2.28229600	-0.78860800
C	-5.92208500	5.67630300	1.45871700
H	-5.37012700	6.46227500	0.93460100
H	-6.98165600	5.75227200	1.19614400
H	-5.81690700	5.84597700	2.53335400
C	-0.95636500	1.49375300	-7.85238700
H	-1.21991000	2.54353900	-7.69291400
H	-0.34512800	1.41521100	-8.75663700
H	-1.88064200	0.93119800	-8.00710800
C	6.89469000	-3.92744600	-1.32873200
H	7.41849700	-3.47032800	-0.48391100
H	7.17863300	-4.98221900	-1.39342900
H	7.21875300	-3.42264000	-2.24254200
C	-0.69467700	-3.00724600	7.32936600
H	0.38563100	-3.04579000	7.49844700
H	-1.18664300	-2.68237300	8.25123900
H	-1.03681000	-4.01720700	7.08876400
C	5.63154700	5.77239500	0.53405500
H	5.20496000	6.37329600	-0.27473300
H	5.95975700	6.44061100	1.33608900
H	6.50473500	5.24484500	0.14162200
H	3.73335200	6.17241600	2.42651500
H	-6.57635500	4.15991400	-0.68717000

H	5.30255100	-5.51094200	0.11837700
H	1.70145000	1.65516000	-7.34023000
H	-0.93370900	-0.35044700	7.48560000
H	-4.24104100	-6.59058100	-0.95023900
C	-5.68013600	-5.20905800	-2.79953600
H	-6.18475200	-6.11775400	-2.45732500
H	-6.43911900	-4.47183400	-3.07389900
H	-5.09523700	-5.44431300	-3.69367000
C	-0.12363200	-1.15881800	-0.33596200
C	-0.95656300	-0.07091100	-0.08895100
C	-0.41975700	1.21216600	0.05472900
C	0.95644000	1.39818000	-0.05290000
S	1.46847100	-0.47184800	1.26229900
H	0.71245600	-1.08108300	-1.03024200
H	-1.96820700	-0.25290900	0.26158600
H	-1.03119400	1.98622500	0.50827200
H	1.52433100	0.87205000	-0.81753300
O	0.91086000	-0.36352000	2.60524500
O	2.81748200	-1.02433900	1.16342600
H	-0.50353500	-2.16637500	-0.19487900
H	1.41860200	2.32142500	0.29376500

Product

0 1

N	-2.51388500	-3.83289700	-3.70660000
N	5.24091800	-1.45814900	-1.99023400

N	-1.74510000	2.46752300	-4.65667300
N	4.05405400	3.07876300	2.42747500
N	1.63331000	4.84803800	2.44623200
N	3.38258900	-1.31583700	-4.20807600
N	3.10421900	-3.09701200	3.82849900
C	-1.57416200	-0.32978500	-4.03991600
H	-2.56589800	0.11311300	-4.09546600
C	3.01503000	5.27111200	2.53523600
H	3.47189200	5.44979500	1.54848000
N	-4.41503500	2.69648500	2.95158100
C	2.34634000	-2.01012900	-3.98063500
H	2.39640200	-3.09946700	-3.82233500
N	-2.08142600	4.62670800	-2.72519600
C	0.99310600	-1.41819100	-3.95573600
C	-0.61918900	1.96595900	-4.35237000
H	0.30185000	2.56407600	-4.30059800
C	-0.45843000	0.51526500	-4.11515600
C	-2.59792400	-2.57064600	-3.79346300
H	-3.56863400	-2.05014600	-3.78057500
C	-3.18762900	2.99071400	3.07228400
H	-2.65023100	2.87194200	4.02595200
C	0.81887700	-0.02992400	-4.05588200
H	1.69695200	0.60819000	-4.09846600
C	-0.11994400	-2.24703900	-3.87718400
H	-0.01157600	-3.32585900	-3.79425700

C	-1.41106300	-1.70385800	-3.90996800
C	3.56404700	-0.50735200	2.67990000
H	3.16387100	-0.55653500	3.68914400
C	5.02801900	-0.40010900	-1.32353800
H	5.16131200	0.60306700	-1.75622900
C	3.80711300	-1.70967600	1.99628900
C	-1.04955900	3.83473500	2.14750700
H	-0.58683200	3.73198900	3.12552600
C	3.49992600	-3.00709000	2.62783700
H	3.63116700	-3.89417100	1.98814100
C	-2.38582500	3.50782100	1.94685700
C	4.32580500	0.74210900	0.76400200
H	4.49711600	1.70889200	0.29719600
C	1.15222200	4.66764900	1.28841400
H	1.74444500	4.81116000	0.37246600
C	-2.95200800	3.65083800	0.67038900
H	-3.99769700	3.38778900	0.53201500
C	4.65586600	-2.00072200	-4.27024100
H	4.58087900	-3.06443800	-3.99057800
C	-2.75269600	4.18416800	-1.74579100
H	-3.78941300	3.82834000	-1.85716300
C	4.31323800	-1.67227600	0.70388400
H	4.49950800	-2.58671100	0.14636300
C	-1.78299300	3.87658900	-4.98877300
H	-0.78585900	4.34440200	-4.96527400

C	3.86048000	4.23693200	3.28284200
H	3.32455100	3.96840000	4.20829200
C	2.79575500	-4.41428400	4.33863000
H	2.90533700	-5.20349400	3.57673100
C	-0.26196500	4.29291800	1.08244700
N	-3.67339900	-4.38041500	-1.12993600
C	3.61396400	1.96298600	2.83306600
H	3.08086800	1.85486800	3.79012700
C	3.83138800	0.71322100	2.07680000
C	5.68251400	-1.31557200	-3.36538400
H	5.73717100	-0.25480100	-3.66000500
C	-2.17616900	4.09797500	-0.39042200
C	-0.82337700	4.40891400	-0.18152000
H	-0.22474900	4.72675700	-1.03138300
C	4.56558300	-0.44185500	0.07767800
C	-2.70048000	4.64641000	-4.03560200
H	-3.68548900	4.15073900	-4.02472300
C	-3.74949000	-4.58206700	-3.56956200
H	-4.62438200	-3.91112300	-3.55713400
N	-4.50030300	-0.13116800	3.61147100
C	-3.70423400	-5.32239300	-2.22883100
H	-2.83539100	-6.00053800	-2.22888900
N	0.43546100	-4.22346000	3.79455300
C	-3.38129300	-1.74042900	2.21196000
C	-2.66318500	-4.41098200	-0.36432300

H	-1.83960000	-5.12674900	-0.51551200
C	-5.09217700	2.16807500	4.11514000
H	-4.44101200	2.12301100	5.00339800
C	-3.47915900	-2.52583600	1.06941400
H	-4.30395600	-2.39952800	0.37192600
C	-1.38008800	-2.94459000	2.86297200
C	-5.62671100	0.76331500	3.82178500
H	-6.25981200	0.82428800	2.92126000
C	-1.48582900	-3.72706000	1.70447600
H	-0.75186200	-4.51230200	1.54026700
C	-2.32118400	-1.95111900	3.10550700
H	-2.27191600	-1.34145900	4.00410100
C	-2.53080300	-3.52596100	0.81022200
C	1.36746600	-4.44125100	4.88887100
H	1.28584700	-3.64252900	5.64429600
C	-0.33433800	-3.21888200	3.87042500
H	-0.29490100	-2.51666800	4.71742600
C	-4.43747000	-0.74998400	2.50702000
H	-5.19276000	-0.61108500	1.71751800
C	-3.86823700	-5.57019200	-4.72659500
H	-3.00077100	-6.23670800	-4.74240000
H	-4.77632000	-6.17288200	-4.62878800
H	-3.90518400	-5.03778500	-5.68044200
C	1.06089200	-5.79327300	5.52858600
H	1.14120600	-6.58952400	4.78255900

H	1.75933800	-6.00105800	6.34495300
H	0.04405100	-5.80442200	5.92940500
C	5.23263900	4.81360100	3.62628700
H	5.75563100	5.10866200	2.71148200
H	5.13240600	5.68881900	4.27534500
H	5.84320600	4.06628500	4.13980700
C	-2.85193700	6.09638100	-4.49036800
H	-1.87607800	6.59094000	-4.49917000
H	-3.28178500	6.14242800	-5.49556100
H	-3.50503000	6.64471000	-3.80645100
C	7.05391400	-1.96925800	-3.51581600
H	7.00279900	-3.02265200	-3.22432700
H	7.40171800	-1.90476600	-4.55138500
H	7.78500600	-1.47370100	-2.87192800
H	5.01852100	-1.93974400	-5.30427200
H	-4.61610000	-5.92461500	-2.13612600
H	3.04042600	6.20858900	3.10462900
H	3.48243300	-4.63269000	5.16600800
H	-2.18638200	3.96686200	-6.00500700
H	-5.94603200	2.81812600	4.34200700
C	-6.44837700	0.24299400	4.99906600
H	-7.29392100	0.90714300	5.20233200
H	-6.83474400	-0.75652300	4.78335900
H	-5.82389100	0.18042800	5.89519700
C	-0.87541400	1.06673600	-0.59198200

C	-1.08165700	-0.41259700	-0.71998000
C	0.00152300	-1.18831400	-0.69589200
C	1.33017900	-0.51421000	-0.53332300
S	0.93107000	1.25971300	-0.48897300
H	-1.30059300	1.49406500	0.32215600
H	-2.08576700	-0.81115000	-0.82876000
H	-0.04907300	-2.26933000	-0.78177800
H	1.82003500	-0.74866800	0.41643700
O	1.42167500	1.92669000	-1.69934600
O	1.30323400	1.79547300	0.82144000
H	-1.22097400	1.63851000	-1.46054600
H	2.02710100	-0.68838800	-1.36083600

3. Dyotropic reaction

Without CC2 cage

Reactant

0 1

C	0.49001600	-0.57783700	-0.00000200
H	0.37237600	-1.19534000	0.89064200
H	0.37235900	-1.19534700	-0.89064200
C	-0.49000100	0.57784800	0.00000400
H	-0.37235100	1.19536900	0.89066000
H	-0.37237500	1.19535900	-0.89065900
Cl	-2.16113500	-0.07047600	0.00002500
Cl	2.16112900	0.07046900	-0.00002600

TS

0 1

C	-0.00035400	0.70906900	0.00020900
H	-0.00088100	1.25553300	0.93321200
H	-0.00078600	1.25553100	-0.93279400
C	-0.00035700	-0.70905600	0.00020900
H	-0.00086500	-1.25552200	0.93321100
H	-0.00079400	-1.25551700	-0.93279400
Cl	2.13749800	-0.00000300	-0.00009600
Cl	-2.13705100	-0.00000300	-0.00010100

Product

0 1

C	0.48991400	0.57801900	-0.00000200
H	0.37245800	1.19559000	0.89048800
H	0.37248900	1.19557400	-0.89050800
C	-0.48992200	-0.57802200	-0.00001000
H	-0.37248500	-1.19559100	0.89049100
H	-0.37247200	-1.19558600	-0.89051300
Cl	2.16130900	-0.07048300	0.00003000
Cl	-2.16130600	0.07048500	-0.00002300

With CC2 cage

Reactant

0 1

N	-2.71469200	-2.08624900	4.59646800
N	3.73831200	2.64073400	3.38720100
N	1.80064700	-5.27133800	1.34964700

N	4.05989800	2.68570300	-3.01316300
N	3.24432300	0.41988200	-4.62036200
N	3.33950000	0.03660500	4.60817600
N	-0.76797900	5.68516300	-0.01532000
C	0.13222800	-3.41354100	2.76231500
H	-0.34093900	-4.27928400	2.30583000
C	4.50066700	1.11586000	-4.79474100
H	5.31334000	0.68616500	-4.18699100
N	-2.65693500	-2.07132900	-4.59739300
C	2.07816500	-0.08585600	4.59377100
H	1.41505200	0.63268100	5.10046500
N	2.09844400	-5.07091200	-1.51785800
C	1.40676800	-1.22088400	3.92943500
C	2.29698700	-4.18470600	1.77448800
H	3.34384800	-3.90299900	1.58349500
C	1.50934500	-3.22650500	2.57467800
C	-2.04020000	-2.76335500	3.76391200
H	-2.48009000	-3.59788200	3.19637400
C	-1.64326900	-1.31075300	-4.57092100
H	-1.64097900	-0.32295900	-5.05739000
C	2.13975400	-2.13348600	3.15664000
H	3.20560900	-1.96656600	3.02713400
C	0.04040900	-1.41088800	4.10340900
H	-0.54881200	-0.72289000	4.70435300
C	-0.60369100	-2.51488100	3.52552000

C	1.42914400	4.04470500	-0.89475000
H	0.78961900	4.48157900	-1.65745500
C	3.92154000	2.39940900	2.15616800
H	4.75246300	1.77310400	1.79762000
C	1.07803200	4.21541100	0.45177800
C	0.76759800	-0.94781100	-4.12675100
H	0.73670300	-0.03705100	-4.71945600
C	-0.10248700	5.01677200	0.83147600
H	-0.34846100	5.02483300	1.90481000
C	-0.37528700	-1.71282400	-3.93028800
C	3.39336500	2.81040800	-0.22960900
H	4.29151900	2.27080000	-0.51687300
C	3.22064600	-0.55660900	-3.81173700
H	4.11383800	-0.88228500	-3.25719600
C	-0.29733500	-2.88864300	-3.17065900
H	-1.20035600	-3.47761500	-3.03260000
C	3.89328700	1.17652500	5.30575700
H	3.12162400	1.78761400	5.80190700
C	0.99294500	-4.57242700	-1.88715300
H	0.03203200	-5.07592400	-1.69806200
C	1.88114400	3.66420400	1.44415400
H	1.63236600	3.78320600	2.49568200
C	2.65700800	-6.14210500	0.57488100
H	3.68362900	-5.75352600	0.47714600
C	4.34272200	2.59596500	-4.43392300

H	3.51227800	3.00319400	-5.03417400
C	-1.88228600	6.46653200	0.47686900
H	-1.99800800	6.39915400	1.57095100
C	1.99547700	-1.34775800	-3.57787200
N	-4.90409900	-0.94312100	3.05617300
C	2.95640800	3.19883800	-2.65891500
H	2.22420500	3.57443200	-3.39063200
C	2.58220100	3.34844800	-1.23843000
C	4.67608800	2.06624400	4.33564900
H	5.42469400	1.43685800	3.82710200
C	0.91557600	-3.29784500	-2.62956000
C	2.06357700	-2.51621200	-2.82842600
H	3.00300200	-2.84935500	-2.39527900
C	3.04602500	2.96044000	1.10770300
C	2.07008200	-6.34642800	-0.82508200
H	1.03766700	-6.71505400	-0.70750800
C	-4.10482200	-2.45645900	4.80069600
H	-4.39943800	-3.28378700	4.13425800
N	-4.76112800	-0.49288600	-3.35030900
C	-4.98589900	-1.24952000	4.46722800
H	-4.68515000	-0.40263900	5.10571600
N	-3.48232300	4.66554700	0.25416600
C	-4.47141100	0.12762700	-1.04547900
C	-4.48123900	0.20632700	2.72908100
H	-4.19790100	0.95921800	3.48130400

C	-3.83370600	-1.59443800	-5.29165100
H	-3.68480400	-0.60249700	-5.74866500
C	-4.58363000	-0.27876900	0.27816800
H	-4.84425700	-1.30370900	0.52908000
C	-3.90097000	2.36329900	-0.30762100
C	-5.02970100	-1.52209000	-4.33817400
H	-5.14571300	-2.51125700	-3.86511300
C	-4.01275700	1.94196700	1.02470500
H	-3.83226000	2.66592600	1.81516400
C	-4.12568600	1.45520000	-1.33428000
H	-4.04300800	1.75499900	-2.37579900
C	-4.35440400	0.62889200	1.32140100
C	-3.18814000	6.01811600	-0.18462700
H	-3.04669300	6.06271000	-1.27723300
C	-3.57353800	3.76469400	-0.63322600
H	-3.43236200	3.98453200	-1.70302300
C	-4.72089700	-0.83965800	-2.13166200
H	-4.88306200	-1.88166800	-1.81509400
C	-4.29011500	-2.87267400	6.25810200
H	-4.00090000	-2.05334200	6.92301200
H	-5.33376200	-3.13662600	6.45437800
H	-3.66259400	-3.73685000	6.49099700
C	-4.34051100	6.93118500	0.22710300
H	-4.48165100	6.89290200	1.31140400
H	-4.13884800	7.96552100	-0.06776400

H	-5.27061500	6.60866300	-0.24790900
C	5.62721600	3.36467100	-4.73246700
H	6.45374400	2.95946500	-4.14117700
H	5.88225500	3.29293300	-5.79416900
H	5.50952000	4.42002200	-4.47334300
C	2.89698200	-7.35669700	-1.61595700
H	3.92295200	-6.99475200	-1.73122900
H	2.91748400	-8.32362300	-1.10407700
H	2.47307300	-7.49980700	-2.61322800
C	5.37228800	3.19831400	5.08719500
H	4.63197400	3.82400900	5.59467500
H	6.06763800	2.79784300	5.83118100
H	5.93167700	3.82986100	4.39222000
H	4.58762500	0.80450700	6.06886300
H	-6.02554000	-1.50968300	4.70133400
H	4.78292000	1.04872000	-5.85266800
H	-1.70430400	7.51548000	0.20984700
H	2.69828500	-7.11676800	1.07620300
H	-4.07114600	-2.31183600	-6.08683000
C	-6.30228100	-1.15704200	-5.09845900
H	-6.51987400	-1.90271400	-5.86922900
H	-7.15321200	-1.10490500	-4.41437200
H	-6.18831600	-0.17841300	-5.57429000
C	1.38817800	-0.00302200	-0.37528700
H	1.26275500	0.98230900	0.07735700

H	1.31265500	0.07219400	-1.46071100
C	0.40504800	-1.01041500	0.18453100
H	0.42328000	-1.01703300	1.27473300
H	0.60976900	-2.01334100	-0.19485100
Cl	3.05775600	-0.56144800	0.00373300
Cl	-1.25491100	-0.56125100	-0.32472200

TS

0 1

N	-2.24708800	-2.19244200	4.78475900
N	3.76366100	2.93774300	3.07346600
N	2.28058300	-5.10056500	1.31556000
N	3.63088000	2.88778500	-3.31783200
N	2.86282600	0.51002500	-4.77138200
N	3.62142600	0.35209300	4.39285900
N	-1.17672700	5.59066700	-0.03379300
C	0.56217200	-3.32889100	2.77123600
H	0.12784000	-4.23654800	2.35963800
C	4.05604100	1.27824700	-5.05610500
H	4.93130400	0.93270400	-4.48340900
N	-2.82650600	-2.38858700	-4.36670400
C	2.36995300	0.16032600	4.43514500
H	1.68705500	0.85649700	4.94718900
N	2.33038400	-4.97932800	-1.56236100
C	1.74229700	-1.03181200	3.82998100
C	2.71020500	-3.95536900	1.65172400

H	3.70272000	-3.58483000	1.35556100
C	1.90581600	-3.04287200	2.48976200
C	-1.57967500	-2.83208900	3.91756200
H	-1.99028800	-3.71064700	3.39603300
C	-1.87227300	-1.55503800	-4.40086800
H	-1.96888500	-0.57657300	-4.89638600
C	2.49062200	-1.89780600	3.01902500
H	3.52654200	-1.64921900	2.80729800
C	0.40727000	-1.31871400	4.09507400
H	-0.19094700	-0.66740900	4.72728500
C	-0.18779000	-2.47697600	3.57467500
C	1.05842400	4.08894500	-1.04264500
H	0.34282300	4.47658300	-1.76325000
C	3.85358500	2.63919900	1.84425500
H	4.66909100	2.02094200	1.44234600
C	0.78811800	4.25169500	0.32336200
C	0.51869600	-0.99399800	-4.07580100
H	0.38327100	-0.09574900	-4.67335100
C	-0.41513700	4.97941400	0.77426300
H	-0.59050600	4.98617900	1.86133100
C	-0.54633700	-1.85104100	-3.82263600
C	3.13567900	2.98079600	-0.50340800
H	4.03580000	2.47768500	-0.84485500
C	2.95141400	-0.41350800	-3.90621100
H	3.88654400	-0.63970000	-3.37351000

C	-0.33239500	-3.01464400	-3.07082700
H	-1.17327500	-3.68056400	-2.89444000
C	4.14012900	1.54843200	5.01745100
H	3.36253300	2.12829300	5.54126300
C	1.16385900	-4.57576500	-1.84835500
H	0.26224500	-5.14855200	-1.58039000
C	1.69238400	3.76589000	1.26303600
H	1.51030000	3.88442700	2.32830000
C	3.14253000	-5.92734600	0.49951300
H	4.12091400	-5.46020800	0.30369600
C	3.82129100	2.76073600	-4.74991600
H	2.92527400	3.08467900	-5.30507600
C	-2.30767500	6.30312200	0.52080400
H	-2.34870600	6.24461500	1.62070400
C	1.80077300	-1.28542700	-3.58629600
N	-4.60060500	-1.24820300	3.37355700
C	2.51417200	3.31549600	-2.89922000
H	1.70361500	3.60822000	-3.58505300
C	2.22897000	3.46354300	-1.45796800
C	4.79930900	2.44632800	3.96578600
H	5.54221300	1.84050400	3.42235700
C	0.93665200	-3.31765500	-2.58955700
C	2.00293400	-2.43907500	-2.83694400
H	2.98506000	-2.68232800	-2.44092100
C	2.87262800	3.12925100	0.85354800

C	2.46377900	-6.23074100	-0.84031800
H	1.47866500	-6.67828300	-0.62803000
C	-3.58760300	-2.66257300	5.08874300
H	-3.86066300	-3.52489100	4.45837700
N	-4.93783300	-0.90998800	-3.02864500
C	-4.57670600	-1.53223700	4.79156100
H	-4.30396800	-0.65277100	5.39759700
N	-3.78525700	4.39210300	0.41636100
C	-4.52312600	-0.22556400	-0.76033100
C	-4.27465100	-0.08017900	3.00403300
H	-3.99679300	0.70376500	3.72583900
C	-4.07010300	-2.00534600	-5.00021500
H	-4.01083400	-1.01997100	-5.49039500
C	-4.50042200	-0.61713100	0.57202800
H	-4.66803100	-1.65338000	0.85410700
C	-4.07354800	2.05928900	-0.09330100
C	-5.20818200	-1.97662100	-3.97571300
H	-5.23757500	-2.95867400	-3.47530000
C	-4.05139700	1.65331600	1.24827000
H	-3.87092000	2.40290800	2.01442800
C	-4.30567800	1.11980900	-1.08980300
H	-4.32741400	1.40762700	-2.13777800
C	-4.26758900	0.32348100	1.58499100
C	-3.61900700	5.75682700	-0.05051500
H	-3.55303600	5.79847100	-1.15035900

C	-3.87569700	3.47612900	-0.45539700
H	-3.82912700	3.69136800	-1.53436800
C	-4.78654500	-1.22745700	-1.81075100
H	-4.85839100	-2.27109000	-1.46700800
C	-3.64650500	-3.05726700	6.56246900
H	-3.37780000	-2.20347600	7.19162100
H	-4.65245100	-3.39322100	6.83190200
H	-2.94192900	-3.86690900	6.76935600
C	-4.80393200	6.59079100	0.43058200
H	-4.87123700	6.55401900	1.52198700
H	-4.69539300	7.63359000	0.11717700
H	-5.73780400	6.19897200	0.01936200
C	5.02688800	3.59757200	-5.16857000
H	5.91732300	3.27302000	-4.62213600
H	5.21272200	3.49610800	-6.24210100
H	4.85570800	4.65336300	-4.94327500
C	3.30794000	-7.19471800	-1.66945100
H	4.28665600	-6.75282700	-1.87827400
H	3.45160900	-8.13851300	-1.13473300
H	2.81961800	-7.40656700	-2.62414900
C	5.48284500	3.63951700	4.62894200
H	4.74789100	4.23857100	5.17502400
H	6.25547400	3.30332100	5.32708300
H	5.94939600	4.27885800	3.87507300
H	4.90425300	1.25011200	5.74520000

H	-5.57806200	-1.86468900	5.09129600
H	4.27559800	1.17801800	-6.12609500
H	-2.21902100	7.35743000	0.23138600
H	3.30569300	-6.87592000	1.02535500
H	-4.31006800	-2.75872500	-5.76035500
C	-6.54375500	-1.70285100	-4.66239400
H	-6.76404000	-2.47794500	-5.40273500
H	-7.35300100	-1.68299100	-3.92792800
H	-6.51629500	-0.73099600	-5.16418600
C	0.99972400	0.36975400	-0.57231200
H	1.08517600	1.29487400	-0.01369300
H	1.13143200	0.38242800	-1.64779800
C	0.83243700	-0.86378000	0.11438300
H	0.78969000	-0.88039900	1.19749300
H	0.83775700	-1.79836600	-0.43392500
Cl	3.18312900	-0.47525700	-0.08492500
Cl	-1.09880000	-0.06914300	-0.39899200

Product

0 1

N	-2.18803300	-2.65762300	4.62704200
N	3.08005300	3.34194400	3.39314000
N	2.88921900	-4.80947900	1.34644800
N	3.37616700	3.46143700	-2.99644800
N	3.04810800	1.04183000	-4.53917900
N	3.26217800	0.70590800	4.60187600

N	-1.97661300	5.38523500	0.00356600
C	0.84799400	-3.33580400	2.74038900
H	0.56523300	-4.27836100	2.27847300
C	4.13015900	1.98079400	-4.74295300
H	5.01915800	1.74357900	-4.13638400
N	-2.15775800	-2.66670000	-4.53761700
C	2.05830400	0.30980200	4.59975700
H	1.26074800	0.86268700	5.12049800
N	3.17608000	-4.60837100	-1.52046900
C	1.64076300	-0.93760200	3.92983500
C	3.13245400	-3.63501800	1.75850600
H	4.09437100	-3.13424000	1.57000600
C	2.15435600	-2.86273000	2.55092800
C	-1.40023400	-3.17210100	3.77761200
H	-1.66545300	-4.07520900	3.20629900
C	-1.34188500	-1.69888100	-4.46865900
H	-1.56393500	-0.71406200	-4.90777100
C	2.54507600	-1.66898800	3.14693500
H	3.55315800	-1.28428500	3.02016200
C	0.34763200	-1.41468200	4.10992400
H	-0.36837100	-0.87395400	4.72350100
C	-0.05279000	-2.62339100	3.52359200
C	0.52532400	4.26983800	-0.88179800
H	-0.18982300	4.57257400	-1.64236600
C	3.29903700	3.13756200	2.16140900

H	4.23790300	2.69235800	1.79846000
C	0.14120400	4.34244700	0.46539700
C	0.92967000	-0.82540000	-4.01654800
H	0.68135700	0.08173000	-4.56205300
C	-1.18526600	4.86754800	0.84743800
H	-1.43023300	4.81073500	1.91945400
C	-0.00888100	-1.83633700	-3.84865600
C	2.69589100	3.44695100	-0.22040400
H	3.68281600	3.09684200	-0.51054000
C	3.25025400	0.07340800	-3.74569600
H	4.20576000	-0.06387600	-3.21689000
C	0.34509300	-2.99712900	-3.14736200
H	-0.40042600	-3.77902900	-3.02733800
C	3.55997400	1.93541900	5.30336700
H	2.67763700	2.36255500	5.80755100
C	1.99071700	-4.37726200	-1.90469400
H	1.16767500	-5.08537400	-1.72141200
C	1.03876500	3.95799500	1.45570400
H	0.76785700	4.01024700	2.50722200
C	3.92857900	-5.48371900	0.59861000
H	4.84434800	-4.87815800	0.50287200
C	3.67245300	3.40468900	-4.41552600
H	2.77667600	3.62053300	-5.02107900
C	-3.23482900	5.90283600	0.49731200
H	-3.33167100	5.81094800	1.59142500

C	2.22920700	-0.96771600	-3.50881900
N	-4.56208700	-1.97594800	3.10649700
C	2.19399800	3.75539300	-2.64653300
H	1.40311400	3.97588700	-3.38029800
C	1.79731900	3.82896600	-1.22650600
C	4.12523200	2.97722800	4.33379300
H	4.98780700	2.52528600	3.81724800
C	1.62894700	-3.14587800	-2.63479000
C	2.57273000	-2.12444100	-2.81903000
H	3.57140800	-2.25800600	-2.41149500
C	2.32415500	3.51085900	1.11679100
C	3.42809300	-5.84496400	-0.80365500
H	2.50522600	-6.43781500	-0.69149100
C	-3.47107700	-3.30327300	4.84179600
H	-3.59790700	-4.17213300	4.17500800
N	-4.55120400	-1.54217700	-3.29040800
C	-4.57871300	-2.29731300	4.51630900
H	-4.45335900	-1.40963800	5.15782700
N	-4.41012200	3.79816400	0.28308000
C	-4.38403800	-0.85413200	-0.99234500
C	-4.38934000	-0.76311800	2.78033600
H	-4.27150900	0.03274600	3.53236800
C	-3.41099100	-2.43522800	-5.22409800
H	-3.47026600	-1.43026400	-5.67265500
C	-4.37729400	-1.26770100	0.33339200

H	-4.39303100	-2.32359800	0.59057100
C	-4.34180600	1.45681000	-0.26770000
C	-4.59432900	-2.61373900	-4.26865800
H	-4.49597900	-3.60090100	-3.78740700
C	-4.33564500	1.02905700	1.06680100
H	-4.31767800	1.77973700	1.85272100
C	-4.36806700	0.51584100	-1.28936800
H	-4.36967300	0.82136900	-2.33258600
C	-4.35931200	-0.32623000	1.37123300
C	-4.41295900	5.18040900	-0.16188400
H	-4.28503800	5.24986200	-1.25480500
C	-4.32562200	2.89389400	-0.60156800
H	-4.24877600	3.13255700	-1.67375100
C	-4.42151700	-1.86068500	-2.07043300
H	-4.34476200	-2.91027300	-1.74637700
C	-3.55827900	-3.74959700	6.29924100
H	-3.43508100	-2.88941100	6.96402300
H	-4.52598900	-4.21846800	6.50202500
H	-2.76801200	-4.46994800	6.52584000
C	-5.73467300	5.82611300	0.24786100
H	-5.86388900	5.76253100	1.33248000
H	-5.76030600	6.87847300	-0.05101400
H	-6.57381800	5.30920600	-0.22479700
C	4.77300400	4.41304800	-4.73460800
H	5.66309000	4.20024900	-4.13510200

H	5.04089700	4.36948100	-5.79467100
H	4.44058200	5.42781800	-4.50116800
C	4.47827400	-6.65358500	-1.56041200
H	5.39577800	-6.06798500	-1.67090300
H	4.71115600	-7.57983700	-1.02635300
H	4.11610700	-6.90962500	-2.55943600
C	4.56740000	4.22957100	5.08681000
H	3.71409900	4.67864800	5.60380800
H	5.33922200	3.98512300	5.82297100
H	4.97141200	4.97001500	4.39162200
H	4.32384700	1.72002100	6.06035700
H	-5.54423600	-2.76251000	4.75012500
H	4.41240500	1.94636800	-5.80262700
H	-3.29068800	6.96544800	0.23098900
H	4.17858900	-6.41358400	1.12405300
H	-3.49911600	-3.17969600	-6.02471300
C	-5.91594700	-2.53516400	-5.02880900
H	-5.97118700	-3.31895900	-5.79038400
H	-6.75811300	-2.65627600	-4.34262300
H	-6.01207100	-1.56020900	-5.51599600
C	0.63715700	0.67512600	-0.67611700
H	0.68450800	1.55275500	-0.03097900
H	1.09027400	0.90324400	-1.64315900
C	1.30425200	-0.52091100	-0.02898200
H	0.93093500	-0.69886200	0.98126000

H	1.18920500	-1.42034700	-0.63493400
Cl	3.06977400	-0.19052900	0.10167300
Cl	-1.09599200	0.30526000	-0.95491800

4. Ring-expansion and contraction reaction

Without CC2 cage

Reactant

0 1

C	0.09074800	1.38281900	0.02831600
C	1.29696800	0.77580800	0.16624900
C	0.43519800	-1.12917800	-0.43530100
C	-0.57163600	-1.21499500	0.44394400
C	-1.71457300	-0.38589300	0.02525400
C	-1.29131100	0.88365300	-0.18987100
H	0.15076700	2.45823300	0.17476000
H	-0.33102200	-1.26004300	1.50599900
H	-2.76370000	-0.66538600	0.01050300
H	-2.02312800	1.66060100	-0.40924700
O	1.62908900	-0.53846900	-0.05525600
H	0.28260400	-1.10345400	-1.51176500
H	2.17940000	1.34451700	0.44024500

TS

0 1

C	-0.17620300	1.43360200	-0.01203000
C	0.98946500	0.71089400	0.42120800
C	0.64113800	-0.76253800	-0.45861600

C	-0.40107300	-1.37536800	0.29572600
C	-1.54287400	-0.63364400	0.06785400
C	-1.37217400	0.75703000	-0.24364400
H	-0.24582200	2.48658200	0.26246400
H	-0.32069200	-2.25043900	0.93162500
H	-2.54341300	-1.00111700	0.27407500
H	-2.26528900	1.35086500	-0.42406100
O	1.86333100	-0.22117200	-0.18188600
H	0.33783000	-0.47845400	-1.46527000
H	1.30107000	0.88208400	1.45326300

Product

0 1

C	-0.31612200	1.48287200	-0.08984900
C	0.77986200	0.60361900	0.41525000
C	0.77992500	-0.60351300	-0.41544300
C	-0.31561600	-1.48282700	0.09066100
C	-1.44885300	-0.73386200	0.12006200
C	-1.44896600	0.73340400	-0.12062400
H	-0.31911900	2.56547600	-0.17609100
H	-0.31690300	-2.56519900	0.17788800
H	-2.41456500	-1.20871200	0.27068500
H	-2.41496700	1.20785900	-0.27088500
O	1.99814900	0.00030600	-0.00022400
H	0.64928500	-0.41099200	-1.48391000
H	0.64970300	0.41096200	1.48376000

With CC2 cage

Reactant

0 1

N	-1.81020200	-3.42502900	4.41814600
N	5.44885500	-1.41778600	1.41466700
N	-1.32657100	-5.44057300	-1.66139900
N	3.68851300	2.13165900	-3.66114500
N	1.18226000	1.74808200	-5.13866200
N	3.96961900	-3.92210800	1.66128700
N	2.86450000	4.42631700	2.26204200
C	-1.03400700	-4.38718600	0.99289000
H	-2.03949100	-4.58402900	0.63014000
C	2.48099500	1.91345000	-5.76044500
H	2.98433100	0.95017100	-5.94410800
N	-4.80915400	1.86279000	-2.77253600
C	2.94369600	-3.66131100	2.35854400
H	3.02036500	-3.26374900	3.38250600
N	-1.92872600	-3.75490600	-3.97718300
C	1.57128200	-3.89846200	1.86945200
C	-0.18409800	-5.06758700	-1.25889600
H	0.70600300	-5.10766500	-1.90592800
C	0.04314100	-4.58073700	0.11639000
C	-1.96067200	-3.74549200	3.20133200
H	-2.95599100	-3.89239900	2.75422900
C	-3.61586100	2.07706900	-3.14280300

H	-3.20369800	3.09447700	-3.22676200
C	1.33792800	-4.33277400	0.55639400
H	2.19102400	-4.47897500	-0.10156500
C	0.49638700	-3.71048400	2.72999900
H	0.64906900	-3.37131900	3.75129700
C	-0.81425400	-3.95260500	2.29431000
C	3.44114200	2.97620900	-0.13325700
H	2.99965400	3.96533700	-0.22390500
C	5.14938900	-0.90939200	0.29263900
H	5.34986300	-1.43423000	-0.65506500
C	3.61076700	2.43427100	1.14920200
C	-1.46895300	1.28355400	-4.08538500
H	-1.16980100	2.31592500	-4.24985600
C	3.23664900	3.21732300	2.34422300
H	3.31977700	2.69413000	3.30970500
C	-2.69712700	0.98286600	-3.51222100
C	4.36417700	0.97315000	-1.10553900
H	4.64149300	0.41666000	-1.99772100
C	0.73401100	0.56515100	-5.03404600
H	1.31791200	-0.30669000	-5.36956900
C	-3.05674300	-0.35818200	-3.31254800
H	-4.02534300	-0.57488500	-2.86889300
C	5.26288500	-3.68094700	2.26535600
H	5.18438600	-3.27179600	3.28586900
C	-2.61267700	-2.78829200	-3.52422600

H	-3.56979900	-2.94458600	-3.00260700
C	4.15062200	1.16185300	1.28977800
H	4.29259700	0.71805100	2.27186300
C	-1.43626000	-5.92584400	-3.01994600
H	-0.47395100	-5.90973600	-3.55727300
C	3.39787800	2.80093500	-4.91895700
H	2.88094500	3.75955500	-4.74698000
C	2.52996200	5.11654000	3.48781700
H	2.67975800	4.49213400	4.38379700
C	-0.58914000	0.25874000	-4.46003700
N	-3.19839000	-0.87107900	4.65961500
C	3.53006600	2.80187900	-2.59546800
H	3.15142400	3.83651900	-2.61166400
C	3.80580400	2.24897100	-1.25701300
C	6.09222100	-2.72045300	1.41083400
H	6.14720800	-3.13519900	0.39062900
C	-2.19738700	-1.38159500	-3.69287100
C	-0.95900800	-1.06552700	-4.26897400
H	-0.29788900	-1.87905000	-4.55630400
C	4.53944300	0.42763800	0.15943000
C	-2.45551400	-5.09748400	-3.80626400
H	-3.39746000	-5.08824300	-3.23325100
C	-3.00494700	-3.25022100	5.22472400
H	-3.91765600	-3.40633100	4.62626600
N	-4.93662000	3.04336000	-0.12055700

C	-3.02562200	-1.81097400	5.74668400
H	-2.10353300	-1.62984400	6.32310600
N	0.21167400	4.41896400	3.48915200
C	-3.54666100	2.07599900	1.59157500
C	-2.30795400	0.01919700	4.50876400
H	-1.43006600	0.08179900	5.17096900
C	-5.62206400	3.01338600	-2.44250900
H	-5.08914400	3.96693900	-2.58935000
C	-3.42361900	1.04548900	2.51509000
H	-4.12989100	0.21918800	2.52810400
C	-1.60019100	3.15601600	2.54351800
C	-6.09549600	2.93095400	-0.98839600
H	-6.60260900	1.96094100	-0.85472600
C	-1.49451500	2.11885100	3.48082700
H	-0.69487400	2.16401900	4.21580800
C	-2.62383500	3.13099800	1.60383000
H	-2.72859000	3.92435500	0.86818000
C	-2.39959700	1.06623200	3.47245800
C	1.07328000	5.58707500	3.45462700
H	0.92456300	6.16899500	2.53016200
C	-0.64541300	4.28216900	2.56433000
H	-0.73686000	5.00459800	1.73855200
C	-4.67157300	2.06153600	0.63662700
H	-5.28868700	1.14927700	0.64344500
C	-2.96711700	-4.24295500	6.38396800

H	-2.06512500	-4.08731100	6.98338800
H	-3.84459600	-4.12032700	7.02611500
H	-2.95196000	-5.26905500	6.00750500
C	0.76309200	6.45731800	4.67101400
H	0.91005100	5.88371700	5.59111700
H	1.41426800	7.33649500	4.69347400
H	-0.27645200	6.79409900	4.64324300
C	4.71456100	3.05098200	-5.65316900
H	5.24204700	2.10552600	-5.81188900
H	4.53363700	3.52223200	-6.62410900
H	5.36085400	3.70586300	-5.06296700
C	-2.69356500	-5.70756900	-5.18584800
H	-1.75988600	-5.72399200	-5.75603600
H	-3.07207500	-6.73035500	-5.09610800
H	-3.42210400	-5.11427900	-5.74441300
C	7.49963500	-2.57215300	1.98527900
H	7.45127400	-2.15734400	2.99655700
H	8.00566900	-3.54165700	2.02391100
H	8.09377300	-1.89345100	1.36787700
H	5.79521100	-4.63898200	2.31633500
H	-3.88309000	-1.70587500	6.42313300
H	2.32665700	2.40935900	-6.72749400
H	3.17560600	5.99987300	3.56626100
H	-1.79500100	-6.96185700	-2.98031500
H	-6.50598000	3.00440300	-3.09208400

C	-7.06128700	4.06974400	-0.67085000
H	-7.93825500	4.02700900	-1.32405200
H	-7.39704400	4.00639900	0.36743200
H	-6.56332800	5.03416900	-0.80859400
C	1.19006000	0.40663100	-1.58817000
C	0.59726400	1.62387400	-1.66886200
C	-0.19140000	1.39001800	0.34018300
C	-0.83059200	0.22061200	0.20580100
C	0.11328600	-0.90785400	0.27599000
C	1.10850100	-0.71919000	-0.62474100
H	1.82905400	0.21587500	-2.45037100
H	-1.65738300	0.16214900	-0.50464800
H	0.00563900	-1.82183600	0.85024500
H	1.85315200	-1.50373100	-0.76978100
O	-0.15713700	2.28122900	-0.72248500
H	0.63617800	1.54087200	1.03214300
H	0.73511200	2.24154500	-2.55369900

TS

0 1

N	0.56887000	-0.68793100	-5.69856300
N	-5.55955700	-2.02896700	-0.59266100
N	2.38723600	-5.04929800	-1.39288800
N	-2.50153300	-0.49834300	4.86058000
N	0.41651600	-0.83603000	5.30944000
N	-3.86775700	-3.74261000	-2.22832700

N	-4.08427800	4.10932400	0.74253900
C	1.10220400	-3.04099400	-2.97945700
H	2.18648300	-3.10930800	-3.01724800
C	-0.63252700	-1.26461400	6.21291900
H	-0.95848600	-2.29965100	6.01919400
N	5.32393600	1.70463800	1.99534700
C	-3.16143200	-2.91984400	-2.88517200
H	-3.61442300	-2.15895600	-3.53985300
N	3.64304700	-4.46809600	1.14283800
C	-1.68535400	-2.94168200	-2.87007000
C	1.14479500	-4.84073100	-1.24805800
H	0.54063400	-5.39613700	-0.51320400
C	0.41056300	-3.87113900	-2.08589700
C	1.15327400	-1.35687600	-4.79422400
H	2.25048000	-1.38662800	-4.70706700
C	4.34241000	1.41025600	2.74224400
H	3.88041000	2.15074500	3.41372900
C	-0.97757500	-3.81537300	-2.03170300
H	-1.53919400	-4.45943800	-1.35918100
C	-0.98836100	-2.12424500	-3.75116600
H	-1.51411300	-1.44925700	-4.42161200
C	0.41073100	-2.17342800	-3.81416400
C	-3.59250500	1.72425200	2.20047700
H	-3.21927000	2.66321500	2.59981900
C	-4.97771300	-1.94552700	0.53058600

H	-4.78983400	-2.83403300	1.15395200
C	-4.17103600	1.72478700	0.92423400
C	2.66431200	-0.17767100	3.61540000
H	2.28847800	0.60149200	4.27449800
C	-4.30111900	2.99373500	0.18168200
H	-4.60515500	2.91501600	-0.87386800
C	3.73856500	0.06557000	2.76879000
C	-3.94564500	-0.65307800	2.36547200
H	-3.84087200	-1.57070500	2.93945400
C	0.91055100	-1.71251200	4.53618100
H	0.53081700	-2.74624200	4.51245500
C	4.21352400	-0.95955400	1.93887800
H	5.05298300	-0.74984100	1.28045900
C	-5.30506600	-3.64385200	-2.37719400
H	-5.60232900	-2.86471200	-3.09801600
C	4.10125400	-3.28787500	1.07673100
H	4.88515000	-3.00374200	0.35715600
C	-4.61523400	0.53008500	0.37188200
H	-5.05601700	0.49962000	-0.62138200
C	3.00456900	-6.05597900	-0.55694400
H	2.29526200	-6.51164800	0.15325300
C	-1.84962400	-0.34388000	6.15065000
H	-1.50291000	0.69198900	6.29981200
C	-4.19730700	5.30960600	-0.05229800
H	-4.52177600	5.11204600	-1.08719300

C	2.04642000	-1.43484800	3.63747200
N	1.49378300	1.99924900	-5.15068900
C	-2.77231300	0.56007900	4.21464200
H	-2.48683000	1.55677500	4.58676200
C	-3.46815800	0.54298200	2.91618800
C	-5.97673900	-3.34848200	-1.03363900
H	-5.66263100	-4.12648200	-0.31812600
C	3.61332100	-2.21210400	1.96128100
C	2.52511200	-2.44360600	2.81310600
H	2.06198600	-3.42696400	2.80622400
C	-4.51018700	-0.66670500	1.09674100
C	4.17585300	-5.46058700	0.22848300
H	4.87535700	-5.00654700	-0.49281700
C	1.39648400	0.05774900	-6.62989500
H	2.46847400	-0.08528800	-6.41550900
N	4.25161900	3.82391800	0.34142300
C	1.07985900	1.54733500	-6.46101400
H	0.00422900	1.70277100	-6.64492400
N	-1.91119100	5.21987400	-0.84209100
C	2.48035600	3.33302400	-1.22483700
C	0.59087700	2.41382400	-4.36307200
H	-0.46892800	2.45281700	-4.65966000
C	5.80489900	3.06845300	2.04599000
H	5.27060600	3.67855700	2.79244100
C	2.18838900	2.81000700	-2.47887400

H	2.95899300	2.33279100	-3.07889400
C	0.18389900	4.06958200	-1.00064600
C	5.66372700	3.73271500	0.67365200
H	6.20520100	3.11177800	-0.05911800
C	-0.09835400	3.53708100	-2.26615700
H	-1.10392800	3.65114300	-2.66258500
C	1.46711000	3.95207700	-0.47774800
H	1.71172300	4.35772500	0.50074100
C	0.89508100	2.90971400	-3.00702000
C	-2.84678500	6.03205800	-0.08554400
H	-2.51168400	6.16705000	0.95594300
C	-0.85247600	4.82567600	-0.26612700
H	-0.62859900	5.06177000	0.78496500
C	3.86979100	3.26530400	-0.73119500
H	4.57151500	2.71659100	-1.37947800
C	1.08335500	-0.40426800	-8.05044400
H	0.02116600	-0.25770200	-8.26788400
H	1.67518900	0.15820600	-8.77897100
H	1.30975100	-1.46750600	-8.16460200
C	-2.97670800	7.39013100	-0.77138000
H	-3.31809200	7.25947500	-1.80264500
H	-3.69161500	8.02469500	-0.23875800
H	-2.00972700	7.89922600	-0.79601600
C	-2.85078700	-0.71410800	7.24462800
H	-3.20399800	-1.73968300	7.10011200

H	-2.39046000	-0.63468500	8.23421100
H	-3.71706100	-0.04843500	7.20845600
C	4.88998100	-6.54398600	1.03330900
H	4.20081000	-6.99150000	1.75570100
H	5.26958800	-7.32937800	0.37261600
H	5.73114600	-6.11695100	1.58550600
C	-7.49655600	-3.36325500	-1.18025400
H	-7.81340000	-2.59148400	-1.88813500
H	-7.84143000	-4.33736700	-1.54043300
H	-7.97404500	-3.15852900	-0.21852000
H	-5.67437500	-4.61093500	-2.74015100
H	1.64731200	2.10825500	-7.21335900
H	-0.22566300	-1.21855300	7.23142600
H	-4.93091000	5.97077300	0.42490800
H	3.39551400	-6.84506200	-1.21144000
H	6.86940200	3.04903200	2.30944000
C	6.25444500	5.14060800	0.69547000
H	7.31302800	5.11101900	0.97130700
H	6.16391500	5.60758500	-0.28865500
H	5.71730800	5.76243800	1.41778800
C	-0.75289900	-0.73432600	1.75555400
C	-0.34649500	0.60159000	2.10196500
C	0.12529000	1.08250000	0.48623000
C	1.37028400	0.42079900	0.27348900
C	1.08229600	-0.91863200	0.09877300

C	-0.09560200	-1.42771500	0.74216000
H	-1.24283600	-1.31735900	2.53949100
H	2.35766500	0.87364100	0.26566800
H	1.77801400	-1.62994300	-0.33958000
H	-0.32639000	-2.48353800	0.61149600
O	-0.41728900	1.86657500	1.46525400
H	-0.66308900	0.75648900	-0.19190200
H	0.14079700	0.70614500	3.07475500

Product

0 1

N	-4.50299600	-1.91755500	3.15215700
N	2.21421600	2.47470900	4.67709300
N	0.79505100	-5.42275700	2.04653300
N	5.11763000	2.24978300	-1.04796400
N	5.01156800	-0.00905500	-2.86250300
N	1.13575800	-0.08019200	5.56233800
N	-0.28782300	5.68518000	-0.27412900
C	-1.24226900	-3.46345100	2.65871800
H	-1.54444200	-4.34158100	2.09319100
C	6.23989300	0.66937400	-2.51153800
H	6.74162500	0.21595000	-1.64107800
N	-0.52137200	-2.24220800	-5.16393500
C	-0.00888400	-0.12791400	5.01966300
H	-0.77581300	0.64053300	5.20296500
N	2.40452600	-5.37834700	-0.39319200

C	-0.41631400	-1.24939500	4.14907800
C	1.10675400	-4.34500800	2.63676300
H	2.14517900	-4.11942600	2.92634200
C	0.10907600	-3.32321500	3.00981400
C	-3.59753000	-2.68102100	2.69977300
H	-3.82898800	-3.53953000	2.05009200
C	0.46906200	-1.55381300	-4.77451700
H	0.72931500	-0.58881400	-5.23689500
C	0.51360300	-2.21930100	3.75041100
H	1.55067300	-2.09845700	4.05277200
C	-1.74812800	-1.38640000	3.77852400
H	-2.49110500	-0.65160900	4.07790900
C	-2.17047300	-2.49986400	3.03754600
C	2.02125000	3.95544600	-0.15864100
H	1.77554900	4.40311600	-1.11755100
C	2.88111000	2.19693600	3.63537300
H	3.73591100	1.50299300	3.65695900
C	1.15839600	4.18078000	0.92425700
C	2.56978700	-1.35082800	-3.47667800
H	2.82801500	-0.45623500	-4.03848500
C	-0.04610100	5.01957500	0.77644500
H	-0.71465100	5.04832000	1.65152900
C	1.37465000	-2.01871100	-3.70511300
C	3.41904400	2.57526400	1.24511600
H	4.30302200	1.95049900	1.34578400

C	4.72262200	-1.07824700	-2.24469300
H	5.37531700	-1.49746800	-1.46268500
C	1.06481900	-3.15545000	-2.94564100
H	0.12264700	-3.66382700	-3.13341300
C	1.41617900	1.03358100	6.44283000
H	0.55362600	1.70939400	6.56138400
C	1.61561700	-4.85090000	-1.23416100
H	0.63240000	-5.29031000	-1.46358100
C	1.43938100	3.60456800	2.15607400
H	0.78765300	3.76689200	3.01092100
C	1.86420700	-6.35565100	1.75941300
H	2.84168100	-6.00906800	2.13271100
C	5.96799000	2.14734900	-2.22195300
H	5.47613600	2.58069100	-3.10835400
C	-1.49287700	6.48566000	-0.29239300
H	-2.04695000	6.44471400	0.65985600
C	3.47560000	-1.82047500	-2.51412400
N	-5.81234500	-0.71776800	0.87929900
C	4.05957000	2.94418000	-1.14370900
H	3.76586800	3.43179800	-2.08656800
C	3.15076000	3.16221100	-0.00088200
C	2.60698100	1.84432200	5.92383300
H	3.45103200	1.15063500	5.77462100
C	1.95536400	-3.63054500	-1.99120500
C	3.17135400	-2.96176500	-1.78442400

H	3.85780300	-3.34967200	-1.03555500
C	2.57330700	2.79629700	2.32313800
C	1.97166500	-6.60607400	0.25260000
H	0.97719100	-6.91698600	-0.10776200
C	-5.88048900	-2.21945100	2.80111600
H	-5.93415500	-3.05924500	2.08885300
N	-2.83998200	-0.51369000	-4.79798600
C	-6.49473200	-0.99040200	2.12440200
H	-6.45872000	-0.13993700	2.82491600
N	-2.95647000	4.72230900	-1.10420000
C	-3.54533700	0.17801100	-2.60459500
C	-5.27900900	0.42375300	0.73693300
H	-5.34498000	1.19990300	1.51558700
C	-1.31885000	-1.71394600	-6.24943200
H	-0.94588200	-0.74461900	-6.61843300
C	-4.30150000	-0.15304300	-1.48691400
H	-4.73357300	-1.14418900	-1.37551900
C	-3.21411700	2.40882400	-1.71751400
C	-2.77643000	-1.54808400	-5.81516900
H	-3.12756900	-2.51720700	-5.42356800
C	-3.98077400	2.06771900	-0.59598700
H	-4.14111100	2.81999400	0.17248900
C	-2.99094200	1.46047500	-2.70838200
H	-2.39085400	1.70179700	-3.58171300
C	-4.51877200	0.79029200	-0.47348600

C	-2.42191000	6.03597400	-1.42309200
H	-1.82867000	6.01082300	-2.35172100
C	-2.66414100	3.76915200	-1.88758300
H	-2.00999900	3.90685200	-2.76024300
C	-3.36973500	-0.81165300	-3.68544000
H	-3.75823800	-1.82087000	-3.47652000
C	-6.64500000	-2.57079100	4.07539900
H	-6.58730000	-1.74340100	4.78885600
H	-7.69696300	-2.77209800	3.85104000
H	-6.21384000	-3.45749200	4.54737800
C	-3.58827200	7.00928600	-1.57812900
H	-4.16806600	7.05265800	-0.65111000
H	-3.22556300	8.01384100	-1.81657600
H	-4.25481300	6.68001300	-2.37944600
C	7.27509900	2.88716700	-1.94302900
H	7.76138700	2.47006400	-1.05605300
H	7.95766100	2.80147700	-2.79415100
H	7.08098100	3.94676700	-1.75756300
C	2.99488100	-7.70095500	-0.03888500
H	3.98144400	-7.40094800	0.32687700
H	2.70686900	-8.63832600	0.44666300
H	3.07041700	-7.87529400	-1.11523900
C	2.99646700	2.92907300	6.92509700
H	2.15908000	3.61599300	7.08007400
H	3.27437000	2.48632500	7.88643100

H	3.84466100	3.50733900	6.54965100
H	1.67839500	0.62745800	7.42747200
H	-7.54557400	-1.21313900	1.90151200
H	6.91882600	0.61093700	-3.37181800
H	-1.20371500	7.52687100	-0.48163800
H	1.62348500	-7.30784100	2.24779100
H	-1.28619800	-2.43616000	-7.07457900
C	-3.64335600	-1.12301600	-6.99863100
H	-3.59674600	-1.86770600	-7.79912900
H	-4.68505400	-1.01095900	-6.68697100
H	-3.30001200	-0.16050400	-7.38943000
C	1.70321200	0.36542200	-0.78230600
C	0.50981200	1.02661600	-1.39083100
C	-0.40233000	1.35917500	-0.29385900
C	-1.13627000	0.10937800	0.06673100
C	-0.20171700	-0.85457900	0.27679200
C	1.23948800	-0.65718000	-0.01636000
H	2.74631000	0.47958900	-1.06450400
H	-2.16907500	0.00967800	0.38681400
H	-0.48452400	-1.79502700	0.74194400
H	1.92532000	-1.40700600	0.37103800
O	-0.19947800	2.26507100	-1.37708600
H	0.09931800	1.77268400	0.58624000
H	0.06105500	0.38650900	-2.15796900

5. 1,5-sigmatropic shift reaction

Without CC2 cage

Reactant

0 1

C	1.57327700	-0.85580000	-0.15858100
C	1.18306600	0.56345000	0.13056000
C	-0.04731400	1.08991700	0.08012700
C	-1.30051100	0.38410900	-0.22665100
C	-1.63012600	-0.83969600	0.19282500
H	2.51221500	-0.88602000	-0.72023300
H	2.00109700	1.23658900	0.38267400
H	-0.14580400	2.16046400	0.25478800
H	-2.02275000	0.94119600	-0.82341100
H	-2.57937600	-1.29184900	-0.07687900
H	0.80160900	-1.37427700	-0.73305800
H	1.73814100	-1.42102800	0.76721200
H	-0.97548300	-1.41695100	0.83923000

TS

0 1

C	-1.30269300	-0.90370500	0.02043900
C	-1.20627000	0.50486500	0.01410500
C	-0.00024400	1.18297500	-0.15535500
C	1.20593300	0.50519600	0.01388500
C	1.30316500	-0.90339600	0.02066800
H	-2.17453200	-1.32960500	0.51620200
H	-2.03962800	1.08776800	0.40304400

H	-0.00035100	2.26691800	-0.08659700
H	2.03927600	1.08855100	0.40217200
H	2.17517700	-1.32863400	0.51666900
H	0.00014600	-1.19097700	0.52489500
H	-1.03906400	-1.45473500	-0.87966000
H	1.03962700	-1.45489500	-0.87917600

Product

0 1

C	-1.25949300	-0.93250500	0.04203900
C	-1.19907000	0.54086500	0.00690500
C	-0.02904400	1.18297500	-0.15535500
C	1.21313300	0.46919600	0.02108500
C	1.34636500	-0.87459600	-0.00093200
H	-2.14573200	-1.33680500	0.53780200
H	-2.07562800	1.10936800	0.30944400
H	-0.00755100	2.26691800	-0.08659700
H	2.00327600	1.06695100	0.49577200
H	2.20397700	-1.32143400	0.49506900
H	-0.65505400	-1.19097700	0.52489500
H	-1.17586400	-1.40433500	-0.97326000
H	0.90282700	-1.50529500	-0.78557600

With CC2 cage

Reactant

0 1

N	-3.34057800	2.53730000	-4.00928600

N	4.71269400	1.28289400	-3.08304400
N	-1.17848200	5.63795700	1.18223200
N	4.94574600	-1.39058700	2.76978600
N	2.95790700	-0.75683500	4.81426900
N	2.95097700	3.60518600	-3.29375900
N	2.42239800	-4.64553000	-2.16011300
C	-1.63164800	4.10021600	-1.20265000
H	-2.49388800	4.36523000	-0.59611000
C	4.38795200	-0.76463600	5.03858600
H	4.85272500	0.21692500	4.84984000
N	-3.41135300	-1.50777500	4.37167300
C	1.79498200	3.16235000	-3.56792600
H	1.59841200	2.55958500	-4.46826600
N	-0.81452600	4.32037300	3.73916400
C	0.61831500	3.45888700	-2.72636600
C	-0.18955000	5.21285900	0.51300000
H	0.84986800	5.38601400	0.83232100
C	-0.35639700	4.47014100	-0.75142700
C	-3.13340400	3.04398100	-2.86631400
H	-3.95421700	3.25211600	-2.16242500
C	-2.14881400	-1.62176900	4.34638800
H	-1.64878800	-2.59682500	4.46002200
C	0.76066900	4.14494700	-1.51184900
H	1.75954700	4.42605700	-1.18806900
C	-0.64888900	3.09046400	-3.16066700

H	-0.78684100	2.55410000	-4.09583900
C	-1.78207800	3.41266700	-2.40065100
C	3.59502100	-2.79346200	-0.30425400
H	3.27285700	-3.76149800	0.07176900
C	4.80100000	0.99341500	-1.85177200
H	5.20082200	1.70442100	-1.11194900
C	3.40448000	-2.50746200	-1.66341600
C	0.12023200	-0.62353600	4.31703800
H	0.55052700	-1.60189100	4.51659600
C	2.77562100	-3.49560900	-2.56081000
H	2.63825800	-3.17372700	-3.60524400
C	-1.25373300	-0.45796000	4.19032300
C	4.56150300	-0.60944300	0.02511100
H	5.00214900	0.11769100	0.70231900
C	2.43453000	0.32873800	4.42037200
H	3.03478100	1.23589600	4.24869400
C	-1.77722900	0.81901900	3.94539500
H	-2.85528600	0.93048900	3.86049500
C	4.02176600	3.30377100	-4.21954600
H	3.68890700	2.68129900	-5.06616000
C	-1.51146600	3.26407800	3.66144700
H	-2.59850900	3.29544100	3.48736900
C	3.80413600	-1.27619700	-2.16563400
H	3.66502400	-1.02639500	-3.21464500
C	-0.89791500	6.34526200	2.41244100

H	0.18168700	6.48085300	2.58852200
C	5.07306300	-1.80979400	4.15413400
H	4.57552100	-2.77857000	4.32631100
C	1.82897200	-5.54035600	-3.12983700
H	1.71862400	-5.07693500	-4.12392300
C	0.98065900	0.47791500	4.21131900
N	-4.58683700	-0.04336200	-3.47333900
C	4.34468500	-2.16927900	1.97045900
H	3.92664100	-3.13378300	2.29946900
C	4.16757800	-1.85202600	0.54054200
C	5.17420400	2.59229800	-3.50743800
H	5.47455100	3.21010500	-2.64501700
C	-0.93333200	1.91776300	3.84185000
C	0.45100800	1.73967000	3.96962900
H	1.09533900	2.61214100	3.89790600
C	4.38536500	-0.31941200	-1.32155000
C	-1.50096300	5.59334900	3.60265900
H	-2.57522200	5.44826400	3.40113000
C	-4.71211600	2.23209900	-4.37586000
H	-5.40696300	2.45853000	-3.55030800
N	-4.35143200	-3.13758100	2.17097100
C	-4.80638700	0.73335100	-4.67456500
H	-4.08985600	0.48853000	-5.47567500
N	-0.45466900	-4.89249400	-2.61996300
C	-3.76801100	-2.48349400	-0.06932200

C	-3.60011000	-0.83983700	-3.46577300
H	-2.93088400	-0.94563600	-4.33395600
C	-4.18954000	-2.71493800	4.54661700
H	-3.56060200	-3.61440600	4.64884900
C	-4.02094200	-1.61264400	-1.12143400
H	-4.77919500	-0.83795000	-1.03845300
C	-2.07320300	-3.59305600	-1.39700600
C	-5.14456300	-2.91259800	3.36592700
H	-5.76022900	-2.00272100	3.27175100
C	-2.32362400	-2.69964600	-2.44653000
H	-1.75273800	-2.80353000	-3.36589800
C	-2.79379600	-3.47974800	-0.21552900
H	-2.60895900	-4.15188100	0.61869600
C	-3.30001600	-1.71865300	-2.31890800
C	0.45834500	-6.02237200	-2.65182000
H	0.58475600	-6.46160300	-1.64845500
C	-1.06737300	-4.66394000	-1.53316500
H	-0.89784800	-5.27229600	-0.63064000
C	-4.51334500	-2.34309300	1.19575300
H	-5.22433700	-1.50307200	1.24096600
C	-5.08597900	3.05107300	-5.60905600
H	-4.40057700	2.82625400	-6.43172700
H	-6.10805600	2.82464500	-5.92757400
H	-5.01751100	4.12027500	-5.39235000
C	-0.10276600	-7.07275900	-3.60874600

H	-0.23494100	-6.64119300	-4.60545800
H	0.57286300	-7.93061100	-3.68083000
H	-1.07686900	-7.42468900	-3.25928300
C	6.55589200	-1.91859300	4.50151200
H	7.05203500	-0.95728300	4.33763000
H	6.68734600	-2.21129400	5.54767600
H	7.04240200	-2.66431200	3.86746000
C	-1.31819200	6.39078300	4.89177700
H	-0.25331100	6.53866200	5.09468200
H	-1.80322700	7.36855900	4.81326600
H	-1.75370400	5.85292700	5.73782800
C	6.35929400	2.41122600	-4.45365500
H	6.06556200	1.79871700	-5.31137700
H	6.71615000	3.38017300	-4.81591800
H	7.18237400	1.90620600	-3.94150300
H	4.39954700	4.25463300	-4.61588700
H	-5.81924600	0.51862300	-5.03693800
H	4.56319200	-1.03548300	6.08714500
H	2.48377000	-6.41557000	-3.22632900
H	-1.36915200	7.33394800	2.35529500
H	-4.78920000	-2.60426600	5.45854700
C	-6.04299300	-4.12501500	3.59874600
H	-6.63197200	-4.00032800	4.51258400
H	-6.72870900	-4.25850600	2.75803800
H	-5.43597500	-5.03075500	3.68952800

C	0.53778661	-1.96668520	0.34154542
C	-0.72085939	-1.33900020	0.73890242
C	-1.16507739	-0.19397120	0.17026542
C	-0.26504039	0.60571880	-0.61590258
C	0.89818861	0.14129780	-1.14744158
H	0.97178961	-2.62265520	1.10548542
H	-1.22000539	-1.68915620	1.64451842
H	-2.05536439	0.27631280	0.56923742
H	-0.40465039	1.68237280	-0.51452758
H	1.66924661	0.86570580	-1.41214558
H	1.18601339	-1.18944680	0.18838058
H	0.51679161	-2.51046920	-0.62848058
H	0.94738161	-0.78377320	-1.73500858

TS

0 1

N	-3.34057800	2.53730000	-4.00928600
N	4.71269400	1.28289400	-3.08304400
N	-1.17848200	5.63795700	1.18223200
N	4.94574600	-1.39058700	2.76978600
N	2.95790700	-0.75683500	4.81426900
N	2.95097700	3.60518600	-3.29375900
N	2.42239800	-4.64553000	-2.16011300
C	-1.63164800	4.10021600	-1.20265000
H	-2.49388800	4.36523000	-0.59611000
C	4.38795200	-0.76463600	5.03858600

H	4.85272500	0.21692500	4.84984000
N	-3.41135300	-1.50777500	4.37167300
C	1.79498200	3.16235000	-3.56792600
H	1.59841200	2.55958500	-4.46826600
N	-0.81452600	4.32037300	3.73916400
C	0.61831500	3.45888700	-2.72636600
C	-0.18955000	5.21285900	0.51300000
H	0.84986800	5.38601400	0.83232100
C	-0.35639700	4.47014100	-0.75142700
C	-3.13340400	3.04398100	-2.86631400
H	-3.95421700	3.25211600	-2.16242500
C	-2.14881400	-1.62176900	4.34638800
H	-1.64878800	-2.59682500	4.46002200
C	0.76066900	4.14494700	-1.51184900
H	1.75954700	4.42605700	-1.18806900
C	-0.64888900	3.09046400	-3.16066700
H	-0.78684100	2.55410000	-4.09583900
C	-1.78207800	3.41266700	-2.40065100
C	3.59502100	-2.79346200	-0.30425400
H	3.27285700	-3.76149800	0.07176900
C	4.80100000	0.99341500	-1.85177200
H	5.20082200	1.70442100	-1.11194900
C	3.40448000	-2.50746200	-1.66341600
C	0.12023200	-0.62353600	4.31703800
H	0.55052700	-1.60189100	4.51659600

C	2.77562100	-3.49560900	-2.56081000
H	2.63825800	-3.17372700	-3.60524400
C	-1.25373300	-0.45796000	4.19032300
C	4.56150300	-0.60944300	0.02511100
H	5.00214900	0.11769100	0.70231900
C	2.43453000	0.32873800	4.42037200
H	3.03478100	1.23589600	4.24869400
C	-1.77722900	0.81901900	3.94539500
H	-2.85528600	0.93048900	3.86049500
C	4.02176600	3.30377100	-4.21954600
H	3.68890700	2.68129900	-5.06616000
C	-1.51146600	3.26407800	3.66144700
H	-2.59850900	3.29544100	3.48736900
C	3.80413600	-1.27619700	-2.16563400
H	3.66502400	-1.02639500	-3.21464500
C	-0.89791500	6.34526200	2.41244100
H	0.18168700	6.48085300	2.58852200
C	5.07306300	-1.80979400	4.15413400
H	4.57552100	-2.77857000	4.32631100
C	1.82897200	-5.54035600	-3.12983700
H	1.71862400	-5.07693500	-4.12392300
C	0.98065900	0.47791500	4.21131900
N	-4.58683700	-0.04336200	-3.47333900
C	4.34468500	-2.16927900	1.97045900
H	3.92664100	-3.13378300	2.29946900

C	4.16757800	-1.85202600	0.54054200
C	5.17420400	2.59229800	-3.50743800
H	5.47455100	3.21010500	-2.64501700
C	-0.93333200	1.91776300	3.84185000
C	0.45100800	1.73967000	3.96962900
H	1.09533900	2.61214100	3.89790600
C	4.38536500	-0.31941200	-1.32155000
C	-1.50096300	5.59334900	3.60265900
H	-2.57522200	5.44826400	3.40113000
C	-4.71211600	2.23209900	-4.37586000
H	-5.40696300	2.45853000	-3.55030800
N	-4.35143200	-3.13758100	2.17097100
C	-4.80638700	0.73335100	-4.67456500
H	-4.08985600	0.48853000	-5.47567500
N	-0.45466900	-4.89249400	-2.61996300
C	-3.76801100	-2.48349400	-0.06932200
C	-3.60011000	-0.83983700	-3.46577300
H	-2.93088400	-0.94563600	-4.33395600
C	-4.18954000	-2.71493800	4.54661700
H	-3.56060200	-3.61440600	4.64884900
C	-4.02094200	-1.61264400	-1.12143400
H	-4.77919500	-0.83795000	-1.03845300
C	-2.07320300	-3.59305600	-1.39700600
C	-5.14456300	-2.91259800	3.36592700
H	-5.76022900	-2.00272100	3.27175100

C	-2.32362400	-2.69964600	-2.44653000
H	-1.75273800	-2.80353000	-3.36589800
C	-2.79379600	-3.47974800	-0.21552900
H	-2.60895900	-4.15188100	0.61869600
C	-3.30001600	-1.71865300	-2.31890800
C	0.45834500	-6.02237200	-2.65182000
H	0.58475600	-6.46160300	-1.64845500
C	-1.06737300	-4.66394000	-1.53316500
H	-0.89784800	-5.27229600	-0.63064000
C	-4.51334500	-2.34309300	1.19575300
H	-5.22433700	-1.50307200	1.24096600
C	-5.08597900	3.05107300	-5.60905600
H	-4.40057700	2.82625400	-6.43172700
H	-6.10805600	2.82464500	-5.92757400
H	-5.01751100	4.12027500	-5.39235000
C	-0.10276600	-7.07275900	-3.60874600
H	-0.23494100	-6.64119300	-4.60545800
H	0.57286300	-7.93061100	-3.68083000
H	-1.07686900	-7.42468900	-3.25928300
C	6.55589200	-1.91859300	4.50151200
H	7.05203500	-0.95728300	4.33763000
H	6.68734600	-2.21129400	5.54767600
H	7.04240200	-2.66431200	3.86746000
C	-1.31819200	6.39078300	4.89177700
H	-0.25331100	6.53866200	5.09468200

H	-1.80322700	7.36855900	4.81326600
H	-1.75370400	5.85292700	5.73782800
C	6.35929400	2.41122600	-4.45365500
H	6.06556200	1.79871700	-5.31137700
H	6.71615000	3.38017300	-4.81591800
H	7.18237400	1.90620600	-3.94150300
H	4.39954700	4.25463300	-4.61588700
H	-5.81924600	0.51862300	-5.03693800
H	4.56319200	-1.03548300	6.08714500
H	2.48377000	-6.41557000	-3.22632900
H	-1.36915200	7.33394800	2.35529500
H	-4.78920000	-2.60426600	5.45854700
C	-6.04299300	-4.12501500	3.59874600
H	-6.63197200	-4.00032800	4.51258400
H	-6.72870900	-4.25850600	2.75803800
H	-5.43597500	-5.03075500	3.68952800
C	0.53538700	-1.95661700	0.35144800
C	-0.66425900	-1.31713200	0.73110500
C	-1.12617700	-0.13670300	0.15066800
C	-0.26154000	0.64528700	-0.61190000
C	0.95478900	0.15136600	-1.13163900
H	0.98709000	-2.61258700	1.09768800
H	-1.13390500	-1.60238800	1.67212100
H	-2.02236400	0.32178100	0.56144000
H	-0.41885000	1.72194100	-0.58132500

H	1.70814700	0.89347400	-1.40224300
H	1.24741300	-0.79241500	-0.11062200
H	0.63829200	-2.36470100	-0.65397800
H	0.92728200	-0.67340500	-1.84310600

Product

0 1

N	-3.34057800	2.53730000	-4.00928600
N	4.71269400	1.28289400	-3.08304400
N	-1.17848200	5.63795700	1.18223200
N	4.94574600	-1.39058700	2.76978600
N	2.95790700	-0.75683500	4.81426900
N	2.95097700	3.60518600	-3.29375900
N	2.42239800	-4.64553000	-2.16011300
C	-1.63164800	4.10021600	-1.20265000
H	-2.49388800	4.36523000	-0.59611000
C	4.38795200	-0.76463600	5.03858600
H	4.85272500	0.21692500	4.84984000
N	-3.41135300	-1.50777500	4.37167300
C	1.79498200	3.16235000	-3.56792600
H	1.59841200	2.55958500	-4.46826600
N	-0.81452600	4.32037300	3.73916400
C	0.61831500	3.45888700	-2.72636600
C	-0.18955000	5.21285900	0.51300000
H	0.84986800	5.38601400	0.83232100
C	-0.35639700	4.47014100	-0.75142700

C	-3.13340400	3.04398100	-2.86631400
H	-3.95421700	3.25211600	-2.16242500
C	-2.14881400	-1.62176900	4.34638800
H	-1.64878800	-2.59682500	4.46002200
C	0.76066900	4.14494700	-1.51184900
H	1.75954700	4.42605700	-1.18806900
C	-0.64888900	3.09046400	-3.16066700
H	-0.78684100	2.55410000	-4.09583900
C	-1.78207800	3.41266700	-2.40065100
C	3.59502100	-2.79346200	-0.30425400
H	3.27285700	-3.76149800	0.07176900
C	4.80100000	0.99341500	-1.85177200
H	5.20082200	1.70442100	-1.11194900
C	3.40448000	-2.50746200	-1.66341600
C	0.12023200	-0.62353600	4.31703800
H	0.55052700	-1.60189100	4.51659600
C	2.77562100	-3.49560900	-2.56081000
H	2.63825800	-3.17372700	-3.60524400
C	-1.25373300	-0.45796000	4.19032300
C	4.56150300	-0.60944300	0.02511100
H	5.00214900	0.11769100	0.70231900
C	2.43453000	0.32873800	4.42037200
H	3.03478100	1.23589600	4.24869400
C	-1.77722900	0.81901900	3.94539500
H	-2.85528600	0.93048900	3.86049500

C	4.02176600	3.30377100	-4.21954600
H	3.68890700	2.68129900	-5.06616000
C	-1.51146600	3.26407800	3.66144700
H	-2.59850900	3.29544100	3.48736900
C	3.80413600	-1.27619700	-2.16563400
H	3.66502400	-1.02639500	-3.21464500
C	-0.89791500	6.34526200	2.41244100
H	0.18168700	6.48085300	2.58852200
C	5.07306300	-1.80979400	4.15413400
H	4.57552100	-2.77857000	4.32631100
C	1.82897200	-5.54035600	-3.12983700
H	1.71862400	-5.07693500	-4.12392300
C	0.98065900	0.47791500	4.21131900
N	-4.58683700	-0.04336200	-3.47333900
C	4.34468500	-2.16927900	1.97045900
H	3.92664100	-3.13378300	2.29946900
C	4.16757800	-1.85202600	0.54054200
C	5.17420400	2.59229800	-3.50743800
H	5.47455100	3.21010500	-2.64501700
C	-0.93333200	1.91776300	3.84185000
C	0.45100800	1.73967000	3.96962900
H	1.09533900	2.61214100	3.89790600
C	4.38536500	-0.31941200	-1.32155000
C	-1.50096300	5.59334900	3.60265900
H	-2.57522200	5.44826400	3.40113000

C	-4.71211600	2.23209900	-4.37586000
H	-5.40696300	2.45853000	-3.55030800
N	-4.35143200	-3.13758100	2.17097100
C	-4.80638700	0.73335100	-4.67456500
H	-4.08985600	0.48853000	-5.47567500
N	-0.45466900	-4.89249400	-2.61996300
C	-3.76801100	-2.48349400	-0.06932200
C	-3.60011000	-0.83983700	-3.46577300
H	-2.93088400	-0.94563600	-4.33395600
C	-4.18954000	-2.71493800	4.54661700
H	-3.56060200	-3.61440600	4.64884900
C	-4.02094200	-1.61264400	-1.12143400
H	-4.77919500	-0.83795000	-1.03845300
C	-2.07320300	-3.59305600	-1.39700600
C	-5.14456300	-2.91259800	3.36592700
H	-5.76022900	-2.00272100	3.27175100
C	-2.32362400	-2.69964600	-2.44653000
H	-1.75273800	-2.80353000	-3.36589800
C	-2.79379600	-3.47974800	-0.21552900
H	-2.60895900	-4.15188100	0.61869600
C	-3.30001600	-1.71865300	-2.31890800
C	0.45834500	-6.02237200	-2.65182000
H	0.58475600	-6.46160300	-1.64845500
C	-1.06737300	-4.66394000	-1.53316500
H	-0.89784800	-5.27229600	-0.63064000

C	-4.51334500	-2.34309300	1.19575300
H	-5.22433700	-1.50307200	1.24096600
C	-5.08597900	3.05107300	-5.60905600
H	-4.40057700	2.82625400	-6.43172700
H	-6.10805600	2.82464500	-5.92757400
H	-5.01751100	4.12027500	-5.39235000
C	-0.10276600	-7.07275900	-3.60874600
H	-0.23494100	-6.64119300	-4.60545800
H	0.57286300	-7.93061100	-3.68083000
H	-1.07686900	-7.42468900	-3.25928300
C	6.55589200	-1.91859300	4.50151200
H	7.05203500	-0.95728300	4.33763000
H	6.68734600	-2.21129400	5.54767600
H	7.04240200	-2.66431200	3.86746000
C	-1.31819200	6.39078300	4.89177700
H	-0.25331100	6.53866200	5.09468200
H	-1.80322700	7.36855900	4.81326600
H	-1.75370400	5.85292700	5.73782800
C	6.35929400	2.41122600	-4.45365500
H	6.06556200	1.79871700	-5.31137700
H	6.71615000	3.38017300	-4.81591800
H	7.18237400	1.90620600	-3.94150300
H	4.39954700	4.25463300	-4.61588700
H	-5.81924600	0.51862300	-5.03693800
H	4.56319200	-1.03548300	6.08714500

H	2.48377000	-6.41557000	-3.22632900
H	-1.36915200	7.33394800	2.35529500
H	-4.78920000	-2.60426600	5.45854700
C	-6.04299300	-4.12501500	3.59874600
H	-6.63197200	-4.00032800	4.51258400
H	-6.72870900	-4.25850600	2.75803800
H	-5.43597500	-5.03075500	3.68952800
C	0.45178113	-1.94629653	0.30080316
C	-0.67886487	-1.29301153	0.65976016
C	-1.16148287	-0.07118253	0.06552316
C	-0.33824587	0.69010747	-0.66944484
C	0.94018313	0.16168647	-1.17538384
H	0.92418413	-2.60226653	1.02634316
H	-1.11401087	-1.50236753	1.64217616
H	-2.06456987	0.37350147	0.49009516
H	-0.51625587	1.76676147	-0.72166984
H	1.67284113	0.92449447	-1.45288784
H	1.40001887	-0.32663547	-0.39807716
H	0.69958613	-2.19568053	-0.74602284
H	0.82297613	-0.54578453	-2.03175084

6. 6-π electron cyclization reaction

Without CC2 cage

Reactant

0 1

C	-0.71700000	1.14078700	-0.19286300

C	-1.69385800	0.06959500	0.05301600
C	-1.53492000	-1.25624800	0.10043100
C	1.75930000	-1.00837000	-0.29589200
C	1.55200100	0.12529300	0.37350400
C	0.61497500	1.18920300	-0.02188100
H	-1.18104800	2.08361400	-0.47841200
H	-2.70154400	0.44872500	0.22219800
H	-2.38272200	-1.89330300	0.33288000
H	2.50209200	-1.73055400	0.02816600
H	2.17735500	0.34493800	1.23964700
H	1.08102300	2.16591600	-0.15658200
H	1.21012900	-1.23398000	-1.20566400
H	-0.58827600	-1.74691700	-0.08012100

TS

0 1

C	0.69931500	1.23275000	0.16378800
C	1.47065400	0.12233100	-0.19698100
C	1.13112800	-1.19980800	0.06400700
C	-1.13112900	-1.19980700	0.06400600
C	-1.47065400	0.12233300	-0.19698000
C	-0.69931300	1.23275100	0.16378800
H	1.17145600	2.21216400	0.11778300
H	2.30421900	0.32230700	-0.87110000
H	1.61882800	-1.98313900	-0.51400600
H	-1.61883000	-1.98313700	-0.51400700

H	-2.30421900	0.32230900	-0.87110000
H	-1.17145400	2.21216500	0.11778400
H	-0.92375800	-1.48298700	1.08243900
H	0.92375700	-1.48298700	1.08244000

Product

0 1

C	-1.27977900	0.73427300	-0.00009600
C	-0.13861100	1.42554100	-0.00004300
C	1.22251700	0.77686000	0.00015000
C	1.22331500	-0.77566000	-0.00017600
C	-0.13718000	-1.42566200	0.00005600
C	-1.27904100	-0.73553900	0.00005300
H	-2.23497500	1.25155700	-0.00014800
H	-0.15748900	2.51305300	-0.00010900
H	1.77815200	1.14933900	-0.86943700
H	1.77881400	-1.14727100	-0.87022900
H	-0.15497100	-2.51319200	0.00018200
H	-2.23372400	-1.25376800	0.00027300
H	1.77926300	-1.14753900	0.86950600
H	1.77760800	1.14894500	0.87029800

With CC2 cage

Reactant

0 1

N	3.28731200	1.77674200	4.37528000
N	-4.28974400	-1.27047700	3.66989600

N	-0.43997900	5.74013600	0.99152500
N	-4.89692500	-1.57668400	-2.73690500
N	-3.53126700	0.24984100	-4.54351900
N	-3.10571300	1.20057700	4.65280200
N	-0.78643000	-5.47972100	0.31427700
C	0.77870800	3.60711900	2.48643700
H	1.43232500	4.29383900	1.95463000
C	-4.93494300	-0.08811400	-4.64577700
H	-5.57939400	0.59739000	-4.07140200
N	2.81989700	1.19367100	-4.80307100
C	-1.85273300	1.01014400	4.61349700
H	-1.36847400	0.19306800	5.17142900
N	-0.94275800	5.45411400	-1.83774000
C	-0.94748600	1.89246600	3.85135900
C	-1.16777200	4.84885500	1.52397400
H	-2.26308200	4.84481200	1.40803200
C	-0.60813200	3.76682700	2.35613900
C	2.76055400	2.52600900	3.49860400
H	3.36494800	3.19144400	2.86257200
C	1.65169400	0.70954000	-4.71304900
H	1.39347200	-0.27710000	-5.12917200
C	-1.46286200	2.91154100	3.03741800
H	-2.54204100	3.01980500	2.96224300
C	0.42837800	1.74415700	3.97621900
H	0.85313800	0.97458700	4.61620300

C	1.29993200	2.60969800	3.30011500
C	-2.73375000	-3.54303400	-0.57183400
H	-2.29271600	-4.19304000	-1.32329700
C	-4.46711300	-1.06106300	2.43204800
H	-5.10617000	-0.24289400	2.06432400
C	-2.37925900	-3.72983500	0.77001000
C	-0.75930700	0.97435100	-4.19047000
H	-0.97271300	0.04101300	-4.70555600
C	-1.39317800	-4.75405700	1.16013800
H	-1.21088900	-4.84897800	2.24241300
C	0.54118600	1.45359400	-4.08494600
C	-4.18174100	-1.72145600	0.05757200
H	-4.87995100	-0.94409600	-0.24245400
C	-3.22189400	1.23620300	-3.80999200
H	-3.98278800	1.82353500	-3.27256200
C	0.77958100	2.66649000	-3.42328800
H	1.80189000	3.03026500	-3.35858400
C	-3.89802400	0.30180000	5.46355700
H	-3.28870800	-0.46625800	5.96741900
C	-0.01027000	4.69183300	-2.23407900
H	1.05102200	4.96710600	-2.13193300
C	-2.94437900	-2.91921700	1.74593500
H	-2.68755000	-3.04424800	2.79477700
C	-1.09822000	6.75352000	0.19670500
H	-2.19368200	6.63255300	0.17535000

C	-5.18211500	-1.51869000	-4.15985800
H	-4.51584100	-2.18850300	-4.72836800
C	0.13739100	-6.47020600	0.82789600
H	0.27996400	-6.38848200	1.91792400
C	-1.83003400	1.70104100	-3.65127900
N	5.12111100	0.08184000	2.88141700
C	-3.98932400	-2.37411400	-2.35289000
H	-3.42716200	-3.00140200	-3.06236500
C	-3.62784400	-2.54276200	-0.93273900
C	-4.96252200	-0.39359000	4.61073000
H	-5.54030800	0.38700100	4.08854900
C	-0.27501900	3.39480400	-2.88712200
C	-1.58291800	2.90348300	-3.00142000
H	-2.39641600	3.49095700	-2.58314300
C	-3.85080300	-1.91068200	1.39462600
C	-0.56904700	6.72384300	-1.24017400
H	0.52674300	6.83810000	-1.19828200
C	4.73042800	1.83821900	4.53382900
H	5.18150800	2.53721500	3.81022500
N	4.58130000	-0.72547400	-3.48141600
C	5.31488900	0.44875700	4.26689600
H	4.85789800	-0.27067100	4.96614000
N	2.13001900	-5.11172000	0.49851800
C	4.30266500	-1.12173500	-1.12795900
C	4.45358400	-0.96896000	2.64214200

H	4.04628000	-1.59692500	3.45017700
C	3.82656300	0.40568600	-5.48059600
H	3.43875000	-0.56049900	-5.84261000
C	4.54766700	-0.66378400	0.16103700
H	5.02727000	0.29639600	0.33360100
C	3.30459400	-3.13239300	-0.21995600
C	5.01973100	0.14366400	-4.55774400
H	5.36976400	1.11558200	-4.17201700
C	3.56291000	-2.66642200	1.07541800
H	3.26084000	-3.28024800	1.92069100
C	3.68184900	-2.36346500	-1.31237600
H	3.48129000	-2.69841600	-2.32652700
C	4.18405800	-1.43902300	1.27073600
C	1.49893800	-6.37228700	0.14051300
H	1.33658700	-6.43846400	-0.94802100
C	2.59926500	-4.40701000	-0.44688300
H	2.49549200	-4.70802300	-1.50154800
C	4.69830400	-0.30455900	-2.29122200
H	5.11338700	0.68938100	-2.06158800
C	5.04631100	2.29101200	5.95746900
H	4.60384000	1.59801300	6.67931700
H	6.12786900	2.32897400	6.11970600
H	4.63124200	3.28509200	6.14275700
C	2.40710100	-7.51417900	0.59572200
H	2.58446800	-7.44687400	1.67338700

H	1.95203000	-8.48396400	0.37219100
H	3.37359700	-7.45720500	0.08820600
C	-6.63764400	-1.91959800	-4.38751400
H	-7.30258100	-1.25521300	-3.82754400
H	-6.89299000	-1.86304900	-5.45011800
H	-6.80989200	-2.94220700	-4.04194000
C	-1.17839500	7.85577700	-2.06340800
H	-2.26598600	7.74532400	-2.10736900
H	-0.93851300	8.82748600	-1.62100700
H	-0.79303000	7.83429900	-3.08604400
C	-5.89223300	-1.23023100	5.48626800
H	-5.32111000	-2.00193100	6.01107200
H	-6.39861900	-0.60065800	6.22427500
H	-6.64937300	-1.72605700	4.87337600
H	-4.40808200	0.89866100	6.22983500
H	6.39340000	0.48785500	4.46374300
H	-5.22188400	-0.03064000	-5.70302600
H	-0.28055000	-7.46143900	0.60958600
H	-0.86440800	7.73431800	0.62864000
H	4.18097400	0.98328900	-6.34344800
C	6.14684900	-0.54870100	-5.32057700
H	6.48605300	0.07099500	-6.15626700
H	6.99622300	-0.73591300	-4.65851400
H	5.80127200	-1.51092900	-5.71029300
C	-0.04705900	-0.89991600	1.25426100

C	-0.67549100	0.34692000	0.78824600
C	-0.11320100	1.26009300	-0.00388300
C	0.37688700	-1.44126000	-1.93389800
C	0.22005600	-2.25860200	-0.88923800
C	0.35141200	-1.96858300	0.54492600
H	0.03701100	-0.97893400	2.34027200
H	-1.66120700	0.55354100	1.21100400
H	-0.63003400	2.17880700	-0.26762200
H	0.22924000	-1.81064700	-2.94595300
H	-0.00968500	-3.30827200	-1.08816400
H	0.73411100	-2.81418500	1.11948200
H	0.66464700	-0.40251800	-1.83080300
H	0.89889700	1.13874600	-0.38107000

TS

0 1

N	-3.29542600	1.78449100	4.38652900
N	4.26151700	3.65162100	1.62707300
N	0.61864200	-3.27552900	4.71709600
N	4.85637200	-0.71174500	-3.09394900
N	3.57211100	-3.32646300	-3.08580000
N	3.08974600	2.57471000	4.03428000
N	0.52918400	4.05188200	-3.55273100
C	-0.69809800	-0.75868800	4.24987700
H	-1.32336500	-1.63761100	4.38415400
C	4.96284400	-3.11145900	-3.42313300

H	5.62820000	-3.18231200	-2.54718700
N	-2.74350400	-4.34045900	-2.47496200
C	1.83286800	2.65486700	3.88459000
H	1.32355600	3.61902200	3.72797000
N	1.12704900	-5.14065800	2.56052200
C	0.95712900	1.46817900	3.96141000
C	1.29880200	-2.26183100	4.37428800
H	2.38809100	-2.31481300	4.22159300
C	0.69197100	-0.92805300	4.19993800
C	-2.72157200	0.65728100	4.29884300
H	-3.28526000	-0.28673500	4.36154000
C	-1.59301900	-3.90471700	-2.78060800
H	-1.36398200	-3.50460200	-3.78080400
C	1.51068700	0.18304200	4.04810400
H	2.59261000	0.08172600	4.02003600
C	-0.42325700	1.62542600	4.01195400
H	-0.87739300	2.61226000	3.96261100
C	-1.25917900	0.50913900	4.15994000
C	2.54725600	2.09103300	-2.85626000
H	2.03783500	1.96502300	-3.80860100
C	4.50268500	2.64762700	0.89121900
H	5.20899400	1.85795500	1.19232100
C	2.18757100	3.17008900	-2.03779800
C	0.82785800	-3.68742800	-2.27686600
H	1.01771200	-3.40963300	-3.31068200

C	1.14029300	4.12641800	-2.44325700
H	0.93427800	4.93274500	-1.72149200
C	-0.46237000	-3.95055800	-1.83058900
C	4.19094700	1.39522900	-1.23205500
H	4.96605700	0.69151400	-0.93834700
C	3.29478200	-3.54463700	-1.86846700
H	4.07350800	-3.57905800	-1.09021400
C	-0.66969400	-4.31314800	-0.49195300
H	-1.68409300	-4.52274600	-0.16257700
C	3.85328600	3.80314600	3.99650800
H	3.22319000	4.68943200	3.81593600
C	0.17315700	-4.86475000	1.77288700
H	-0.88108200	-4.95550600	2.07696100
C	2.83517000	3.34788400	-0.82365100
H	2.56995200	4.16984600	-0.16369700
C	1.32372200	-4.52718700	4.88758900
H	2.41177400	-4.42642000	4.74268200
C	5.15004100	-1.74003700	-4.07699700
H	4.46030100	-1.68023600	-4.93520200
C	-0.42454700	5.09901600	-3.85834800
H	-0.59026200	5.78036300	-3.00774000
C	1.91763800	-3.79664300	-1.40188100
N	-5.23302300	1.79270000	2.20948700
C	3.90586700	0.08780200	-3.35009700
H	3.30974500	0.00422600	-4.27260300

C	3.53984300	1.20430000	-2.45921800
C	4.93122200	3.73583000	2.91057400
H	5.55076400	2.84347900	3.09974600
C	0.40497400	-4.42879700	0.38165500
C	1.70189000	-4.16568300	-0.07989700
H	2.53067600	-4.26393500	0.61653400
C	3.84332200	2.46316800	-0.41544900
C	0.79118100	-5.57474800	3.90457200
H	-0.30063200	-5.64117300	4.04247800
C	-4.73301500	1.78586900	4.60356500
H	-5.13222200	0.75836200	4.62673400
N	-4.63025600	-2.13814700	-2.87136300
C	-5.41219300	2.52227600	3.44610500
H	-5.01611000	3.54979900	3.39462700
N	-2.40763400	3.86511200	-3.17737100
C	-4.43450900	-0.17289800	-1.50416500
C	-4.65703600	2.39776400	1.25617300
H	-4.31701900	3.44111800	1.35098200
C	-3.77083800	-4.31041100	-3.49373300
H	-3.42076800	-3.86412100	-4.43878300
C	-4.69519500	0.41092000	-0.27056300
H	-5.13160700	-0.16482600	0.54168500
C	-3.54757800	1.93016400	-2.31080200
C	-4.99747400	-3.53564500	-3.00573600
H	-5.30687500	-3.96493100	-2.03832200

C	-3.83337200	2.51567400	-1.07105500
H	-3.58123000	3.56246900	-0.91909100
C	-3.85695500	0.59455900	-2.52482600
H	-3.63634200	0.11425200	-3.47447600
C	-4.39752600	1.76315500	-0.04945600
C	-1.77053900	4.52814900	-4.30368800
H	-1.58530000	3.81997600	-5.12825100
C	-2.85680800	2.69354400	-3.36614200
H	-2.73495600	2.17204900	-4.32884800
C	-4.76152000	-1.59367100	-1.73401600
H	-5.13726700	-2.14860800	-0.86017600
C	-5.02520500	2.48418300	5.92973400
H	-4.63277700	3.50534700	5.91152400
H	-6.10263100	2.52198200	6.11759600
H	-4.54626500	1.95112100	6.75509900
C	-2.68924000	5.65068200	-4.78561100
H	-2.88399200	6.35368800	-3.96995200
H	-2.23307200	6.19368200	-5.61897600
H	-3.64720300	5.24235800	-5.11809200
C	6.58921600	-1.56522500	-4.55589900
H	7.27817100	-1.63086800	-3.70847000
H	6.85069300	-2.33745300	-5.28586100
H	6.71885400	-0.58583000	-5.02354400
C	1.43310900	-6.93500500	4.16240200
H	2.51782500	-6.86900600	4.03568800

H	1.21518000	-7.27855100	5.17819300
H	1.05314900	-7.67677500	3.45520000
C	5.80199500	4.98941700	2.93823500
H	5.19031400	5.87603400	2.74630500
H	6.29045500	5.09988300	3.91122200
H	6.57341900	4.93513700	2.16569900
H	4.35016100	3.92214700	4.96747000
H	-6.48688300	2.57492400	3.66031700
H	5.25927500	-3.87964100	-4.14800600
H	-0.01534500	5.68484600	-4.69172300
H	1.13977400	-4.88724300	5.90715600
H	-4.07597400	-5.34580200	-3.69076200
C	-6.13951200	-3.64865100	-4.01314700
H	-6.42723700	-4.69470800	-4.15601200
H	-7.01260900	-3.09190400	-3.66316800
H	-5.83368500	-3.23035800	-4.97681900
C	0.05067500	1.44600200	0.31576300
C	1.12455300	0.55241600	0.43349600
C	1.30532800	-0.63726800	-0.26530300
C	0.00971000	-0.32881000	-2.09914600
C	-0.35112600	1.01057100	-2.06990300
C	-0.61567200	1.70315400	-0.88539400
H	-0.02831600	2.21927900	1.07895000
H	1.97525100	0.94617200	0.99517700
H	2.32792400	-1.00228500	-0.38069700

H	0.45471600	-0.74447600	-3.00320500
H	-0.27289000	1.61644400	-2.97487600
H	-1.11376800	2.66920800	-0.96534300
H	-0.60595500	-1.02714500	-1.55222100
H	0.58125200	-1.43331800	-0.18688800

Product

0 1

N	3.61381200	2.21476800	3.88041600
N	-3.31729400	-2.12055900	4.19121200
N	-1.27072900	5.58311000	1.38709900
N	-4.92010200	-2.37466900	-2.02398300
N	-4.28145200	-0.25253700	-3.88921700
N	-2.44417600	0.50596200	5.12575800
N	0.27293400	-5.54942100	0.11532800
C	0.54904900	3.68654200	2.57465700
H	0.97670400	4.50993400	2.00819400
C	-5.58903900	-0.86416000	-3.79160700
H	-6.24960600	-0.34322300	-3.07932700
N	1.65865900	1.82580500	-5.08185800
C	-1.20356800	0.54230800	4.86783600
H	-0.49894000	-0.19750300	5.27832000
N	-2.23387000	5.29050000	-1.33769400
C	-0.60531500	1.60670700	4.03774500
C	-1.72285500	4.55044800	1.96707000
H	-2.80192200	4.33702200	2.01923400

C	-0.84707900	3.56982100	2.63766900
C	2.82924700	2.93639600	3.19403500
H	3.19902500	3.74926800	2.54980100
C	0.62013200	1.14325600	-4.83209300
H	0.46513300	0.13750300	-5.25329300
C	-1.41568200	2.53128100	3.36541700
H	-2.49488100	2.42519100	3.43937300
C	0.77680400	1.72742900	3.96411900
H	1.42795400	1.02891400	4.48375300
C	1.36225900	2.77138400	3.23365300
C	-2.10370600	-3.96885000	-0.35910900
H	-1.68302400	-4.50816800	-1.20455800
C	-3.71862000	-1.90575700	3.00812600
H	-4.53415300	-1.19944900	2.78749100
C	-1.50584100	-4.12103100	0.89964800
C	-1.68687100	0.97533200	-3.94265400
H	-1.81729500	0.03326900	-4.46934400
C	-0.30798300	-4.96237000	1.07847000
H	0.05610300	-5.04978800	2.11456600
C	-0.48301000	1.66676800	-4.00111400
C	-3.71937700	-2.44940300	0.58961800
H	-4.58318800	-1.80529600	0.44553700
C	-4.05938800	0.77452300	-3.18012100
H	-4.82569200	1.20177700	-2.51469100
C	-0.35219800	2.88310200	-3.31676200

H	0.59410100	3.41427700	-3.37884600
C	-2.91983500	-0.55320800	5.98924600
H	-2.10953100	-1.21296800	6.34007900
C	-1.26868200	4.70777600	-1.91728300
H	-0.26291800	5.15342400	-1.96439600
C	-2.03124700	-3.44697600	1.99272200
H	-1.58716700	-3.54909000	2.97945500
C	-2.23057700	6.47572300	0.77378500
H	-3.27257100	6.15319300	0.93250200
C	-5.46402500	-2.33044600	-3.36947400
H	-4.79511900	-2.83329500	-4.08749100
C	1.41695400	-6.37823100	0.43803400
H	1.71483200	-6.29171600	1.49591900
C	-2.76927800	1.49093200	-3.21586700
N	5.42617000	0.89793200	2.02337700
C	-3.83452100	-3.00531300	-1.84704000
H	-3.30889600	-3.50502000	-2.67613500
C	-3.20508400	-3.13841800	-0.51908200
C	-3.97103700	-1.40362000	5.27096000
H	-4.75076400	-0.72472000	4.88763000
C	-1.41947600	3.40487100	-2.59497800
C	-2.63158800	2.70128300	-2.54649800
H	-3.45727800	3.12656500	-1.98169800
C	-3.14239300	-2.60519400	1.84324900
C	-1.97238700	6.58484900	-0.73216100

H	-0.92107900	6.88593900	-0.87242100
C	5.03419500	2.51527300	3.81552300
H	5.24010600	3.30675000	3.07612800
N	3.92959300	0.19172800	-4.19331700
C	5.78693100	1.25710500	3.37728500
H	5.58495500	0.44935700	4.09990900
N	3.09537000	-4.70903000	-0.13202800
C	4.16575300	-0.33543300	-1.85941400
C	4.96316200	-0.26616900	1.82976300
H	4.84539900	-0.99048800	2.65146700
C	2.65135600	1.24336300	-5.95919400
H	2.37320300	0.23512000	-6.30702900
C	4.54879100	0.12355000	-0.60650700
H	4.84830200	1.15734200	-0.45531200
C	3.76782800	-2.53585100	-0.93013200
C	4.01319000	1.16734400	-5.26507100
H	4.25151600	2.17129700	-4.87637400
C	4.17151000	-2.06777900	0.32746200
H	4.15444300	-2.75600600	1.16930900
C	3.77619400	-1.67333200	-2.01690200
H	3.46427700	-2.01080500	-3.00212600
C	4.55784300	-0.74471900	0.49469700
C	2.62093700	-6.04766500	-0.44423100
H	2.30101200	-6.11921200	-1.49698800
C	3.26638300	-3.91131900	-1.10360400

H	3.02847400	-4.20074400	-2.13978300
C	4.18275600	0.58312500	-3.01420200
H	4.44686700	1.62847800	-2.79039600
C	5.50034200	2.96538700	5.19856400
H	5.29445100	2.18502600	5.93729400
H	6.57440100	3.17496400	5.19478600
H	4.96991100	3.87101700	5.50410300
C	3.75639900	-7.03675500	-0.18315700
H	4.08607600	-6.96459400	0.85776000
H	3.43013000	-8.06251300	-0.38040800
H	4.61148000	-6.81313300	-0.82641200
C	-6.83172200	-3.00906800	-3.37176300
H	-7.49829000	-2.51416300	-2.65915300
H	-7.28303300	-2.96607900	-4.36775800
H	-6.73767100	-4.05747900	-3.07713100
C	-2.89836000	7.62113400	-1.36374900
H	-3.94294700	7.32862300	-1.22121700
H	-2.74130800	8.60561100	-0.91257200
H	-2.71032400	7.69651100	-2.43784600
C	-4.59015400	-2.41725800	6.23026700
H	-3.81742300	-3.08692900	6.61956700
H	-5.07478100	-1.90936200	7.06966900
H	-5.33686600	-3.02510400	5.71285100
H	-3.39306300	-0.08874100	6.86326700
H	6.86127500	1.47920500	3.39683600

H	-6.05446300	-0.83196900	-4.78458000
H	1.13612500	-7.42184400	0.24539000
H	-2.10301600	7.47117100	1.21637700
H	2.74909500	1.89785900	-6.83433800
C	5.09295500	0.72187400	-6.24882300
H	5.17073200	1.42612400	-7.08273300
H	6.06343500	0.66496700	-5.74913200
H	4.85557000	-0.27047600	-6.64390700
C	0.99395000	-0.51714900	1.11637700
C	0.19712600	0.43478100	0.61478500
C	-0.72283600	0.12622000	-0.54069900
C	-0.12775700	-0.93678200	-1.46863500
C	0.46280100	-2.07783700	-0.68058600
C	0.99555800	-1.86189200	0.52763400
H	1.63913700	-0.31015400	1.96819200
H	0.16405100	1.42513100	1.06193900
H	-1.68156500	-0.24281600	-0.13821700
H	-0.89721900	-1.29540700	-2.16299000
H	0.50168300	-3.07316500	-1.12188200
H	1.47005900	-2.68101500	1.06665600
H	0.66839600	-0.48547300	-2.08660600
H	-0.94833000	1.03457000	-1.10734500

7. Claisen rearrangement reaction

Without CC2 cage

Reactant

0 1

C	-0.70143800	1.08982600	0.20880800
C	0.79668400	1.00288000	-0.11561800
C	-2.13880900	-0.96466900	0.15589100
C	-1.47454000	-0.00186600	-0.47623400
C	1.39251000	-0.29836900	0.37139300
H	-2.66334200	-1.74670800	-0.38337500
H	-1.44347000	0.00200500	-1.56621000
H	1.03739500	-0.62993000	1.37258500
O	2.20111300	-0.94858200	-0.23971300
H	-2.18384500	-1.00209800	1.24209600
H	-0.84969300	1.01840000	1.29361200
H	0.98953800	1.08600600	-1.18952700
H	1.33308500	1.82137500	0.38373400
H	-1.07501400	2.07279700	-0.10064300

TS

0 1

C	-0.41271600	1.45523300	0.32284100
C	1.47479000	0.62337800	-0.29124600
C	-1.33587600	-0.74467700	0.18759600
C	-1.29085500	0.57417300	-0.28744900
C	1.19415100	-0.61894300	0.24849900
H	-1.93935500	-1.48369000	-0.32827900
H	-1.59651100	0.76343100	-1.31289600
H	1.55189300	-0.84241000	1.26537900

O	0.30276400	-1.39034400	-0.26207100
H	-1.24828900	-0.91505400	1.25778100
H	-0.22120800	1.36913100	1.38890600
H	1.27154200	0.79849700	-1.34128900
H	2.23889800	1.24605800	0.16693100
H	-0.25604000	2.45180900	-0.08141000

Product

0 1

C	-2.37835900	0.83216600	0.08997200
C	2.56948400	0.60519300	-0.12288700
C	-0.64421200	-0.95292500	0.28844100
C	-1.53578600	-0.02197800	-0.48151600
C	1.31981400	0.32761400	0.23027800
H	-0.90911800	-1.99583100	0.09176200
H	-1.45352000	-0.08211700	-1.56558800
H	0.70851300	1.01424500	0.81609700
O	0.71500300	-0.84254400	-0.10996400
H	-0.74416300	-0.77228500	1.36848200
H	-2.46008600	0.90688700	1.17228900
H	3.15197400	-0.09224300	-0.71375100
H	3.02199900	1.54004500	0.18049400
H	-3.02126900	1.48124000	-0.49580300

With CC2 cage

Reactant

0 1

N	2.51215900	-4.86041800	1.95806000
N	-4.18048200	-3.02648600	-2.18796000
N	-1.42208500	-1.04515000	5.32244600
N	-3.91027400	3.43148400	-2.27786500
N	-2.69892100	5.13513000	-0.26801700
N	-3.68805800	-4.34135600	0.33276600
N	0.28542200	-0.06271200	-5.65027300
C	-0.05164000	-2.75391700	3.45840700
H	0.53589400	-2.29580800	4.25032800
C	-4.00087600	5.38956600	-0.84682900
H	-4.81819200	4.92111300	-0.27495600
N	3.38665700	4.50137900	1.75917800
C	-2.43367000	-4.51418000	0.38560500
H	-1.89323100	-5.12532400	-0.35436900
N	-1.42825000	1.86791400	5.08566600
C	-1.61090100	-3.91086300	1.45245000
C	-2.03964400	-1.48677700	4.30652900
H	-3.08345600	-1.21197900	4.08615700
C	-1.41156800	-2.42435600	3.35515400
C	1.94912300	-4.00408600	2.70504700
H	2.49385600	-3.48532200	3.50941800
C	2.31631800	4.61396800	1.08979200
H	2.29169900	5.10193700	0.10305300
C	-2.18537100	-3.01041900	2.36197300
H	-3.24276300	-2.77765600	2.26193900

C	-0.26410700	-4.22666100	1.55866500
H	0.20569000	-4.92122800	0.86662100
C	0.52229100	-3.65344200	2.56968200
C	-1.64770200	1.02933300	-3.81283000
H	-0.98449100	1.72767600	-4.31689100
C	-4.29677800	-1.77977700	-1.98628600
H	-5.08838900	-1.35683800	-1.35386000
C	-1.43980100	-0.34449800	-3.99803700
C	-0.15532300	4.45711600	0.93390000
H	-0.13531000	5.04453100	0.01935300
C	-0.38013900	-0.83784000	-4.89991200
H	-0.23034200	-1.92886700	-4.90786000
C	1.01921700	4.12796600	1.60110500
C	-3.55272400	0.55744100	-2.40520700
H	-4.37563100	0.92595400	-1.79662500
C	-2.65820500	4.39673600	0.76131800
H	-3.57079100	3.97694100	1.21368000
C	0.95849200	3.36495900	2.77645600
H	1.88826200	3.11787800	3.28237600
C	-4.41120800	-4.96070900	-0.75498100
H	-3.75415400	-5.52933000	-1.43379600
C	-0.32587600	2.18848400	4.54808400
H	0.64385900	1.93529100	5.00403000
C	-2.28722200	-1.25364300	-3.37660200
H	-2.15778300	-2.32441000	-3.51246900

C	-2.13876200	-0.15547700	6.20994100
H	-3.16612400	0.05109400	5.86885600
C	-4.04872400	4.87844600	-2.28919900
H	-3.22033200	5.35253000	-2.84114400
C	1.27150100	-0.66309500	-6.52281400
H	1.28961500	-1.76277300	-6.45019800
C	-1.39668600	4.04927800	1.44309700
N	4.67669600	-3.44575800	0.63655400
C	-2.91774300	2.93769400	-2.89294500
H	-2.17539100	3.57293200	-3.40110900
C	-2.69572300	1.48370500	-3.02028800
C	-5.16111300	-3.89895000	-1.56366000
H	-5.80744100	-3.33794800	-0.87016600
C	-0.26673400	2.95517000	3.28721300
C	-1.44561500	3.30905600	2.61723300
H	-2.39678600	2.99018900	3.03661500
C	-3.35876600	-0.80762200	-2.58987400
C	-1.38959400	1.17022000	6.35874300
H	-0.35332900	0.94272900	6.65859800
C	3.91160700	-5.15779800	2.20750600
H	4.31725100	-4.52392700	3.01327900
N	5.12774600	2.93510300	0.06594000
C	4.70421300	-4.86181900	0.93173300
H	4.29746100	-5.47507500	0.11105000
N	3.03035100	-0.57418900	-4.86768200

C	4.48807100	0.67481200	-0.44631500
C	4.18154100	-3.08913200	-0.47485100
H	3.78990300	-3.82020500	-1.19982900
C	4.60347000	5.01951500	1.17327000
H	4.43697600	5.48360800	0.18746000
C	4.51176500	-0.64889400	-0.02627800
H	4.85002400	-0.91432100	0.97205700
C	3.65866000	-0.02886900	-2.61059100
C	5.63688300	3.89922900	1.02471900
H	5.78162900	3.43920900	2.01627200
C	3.69070800	-1.36212800	-2.17915600
H	3.38737000	-2.14079200	-2.87458800
C	4.05316800	0.98112300	-1.74310000
H	4.04905700	2.02180800	-2.05674300
C	4.11813000	-1.67603600	-0.89529400
C	2.66838800	-0.12539500	-6.20027500
H	2.62790400	0.97511500	-6.25449500
C	3.25385400	0.31102200	-3.98823400
H	3.18065800	1.38668800	-4.21218500
C	4.96502600	1.74023900	0.45722300
H	5.19587700	1.42694100	1.48736700
C	4.04021000	-6.63011700	2.59133800
H	3.63932900	-7.26301500	1.79397500
H	5.08834900	-6.89495000	2.76076700
H	3.47669800	-6.83674300	3.50485300

C	3.69444100	-0.65754900	-7.19740300
H	3.73404100	-1.74986000	-7.14939100
H	3.43451200	-0.35683400	-8.21693200
H	4.68972300	-0.27100100	-6.96348500
C	-5.38044600	5.23820800	-2.94339100
H	-6.20568200	4.77096400	-2.39795900
H	-5.52752000	6.32256600	-2.94855100
H	-5.40797800	4.87818800	-3.97504700
C	-2.05512800	2.04971900	7.41515500
H	-3.08529000	2.27429900	7.12306400
H	-2.06423900	1.54530200	8.38611300
H	-1.51710700	2.99555400	7.51900000
C	-6.00550900	-4.55253900	-2.65536900
H	-5.36289500	-5.09879300	-3.35256000
H	-6.72807700	-5.24960000	-2.21994400
H	-6.55222200	-3.79307700	-3.22052300
H	-5.14883900	-5.64971500	-0.32527800
H	5.74793600	-5.15421100	1.10026800
H	-4.16315400	6.47416600	-0.86263100
H	1.02557000	-0.38629000	-7.55524600
H	-2.18917900	-0.63201200	7.19691200
H	5.01325700	5.77818100	1.85122200
C	6.96316700	4.45381300	0.51087900
H	7.35979700	5.20675400	1.19878600
H	7.69882100	3.65157200	0.41102300

H	6.82340300	4.91134900	-0.47305800
C	-0.71205900	0.60239500	0.12890200
C	-2.04898900	0.58932300	0.87612000
C	0.68460600	-1.45452600	-0.21987300
C	0.20141700	-0.50400400	0.57648000
C	-2.90093100	-0.61864000	0.56526600
H	1.34705200	-2.23121500	0.15298500
H	0.46503300	-0.50676500	1.63603900
H	-2.39714500	-1.45801000	0.04081400
O	-4.07209500	-0.69116400	0.86321800
H	0.43734800	-1.47898500	-1.28032500
H	-0.88925800	0.52157400	-0.95195900
H	-1.87854600	0.60179700	1.96395600
H	-2.64818900	1.47884200	0.64616700
H	-0.23049900	1.57366400	0.30593900

TS

0 1

N	4.06942500	1.32372600	3.98547400
N	-4.02995200	0.31265600	3.97782700
N	1.21564200	5.63484000	0.13964500
N	-5.29686300	-0.75669800	-2.22543900
N	-3.63609600	0.41301400	-4.32991300
N	-2.23279700	2.54589700	4.58657100
N	-2.19752300	-5.20458500	1.24377100
C	1.99411800	3.55533500	1.99083000

H	2.75216600	3.92823000	1.30675000
C	-5.08420500	0.44571600	-4.31674900
H	-5.48203600	1.34688700	-3.82216400
N	2.71068300	-0.32566800	-5.15503500
C	-1.03932900	2.11664800	4.61772200
H	-0.72070500	1.33567700	5.32643800
N	0.41419200	5.11131500	-2.63216400
C	0.00956300	2.62976900	3.71818800
C	0.34686100	5.07823800	0.87741300
H	-0.72038600	5.34535200	0.82006900
C	0.69043900	4.05732700	1.88400200
C	3.69284100	2.11400900	3.06728200
H	4.39394000	2.49749500	2.30915400
C	1.45918900	-0.44602200	-4.99377800
H	0.92065400	-1.36822100	-5.26172700
C	-0.29326100	3.59326100	2.74635300
H	-1.31518300	3.95742000	2.67679500
C	1.30524900	2.14178400	3.81852000
H	1.56619500	1.39357000	4.56269800
C	2.30660900	2.60503100	2.95437800
C	-3.62488200	-2.96497800	0.13872100
H	-3.43410600	-3.81628000	-0.50971600
C	-4.33406600	0.34205400	2.74765400
H	-4.84260700	1.20588000	2.29094100
C	-3.12837700	-2.99692000	1.45020500

C	-0.75341600	0.51286500	-4.41175200
H	-1.23830200	-0.39155800	-4.77095600
C	-2.37747400	-4.16656700	1.94810600
H	-1.99236500	-4.08370400	2.97635100
C	0.62917400	0.64809400	-4.45138800
C	-4.53254100	-0.76466900	0.53585200
H	-5.07541900	0.09688500	0.15480500
C	-3.02515100	1.43278500	-3.88714100
H	-3.56370000	2.30282600	-3.47923300
C	1.22366000	1.82531300	-3.97428500
H	2.30665300	1.91042100	-4.00984900
C	-3.17487600	1.97845500	5.52546200
H	-2.73951300	1.16430100	6.12829300
C	1.08389400	4.09805700	-2.99380400
H	2.18472900	4.09295500	-2.98954100
C	-3.34383200	-1.91170200	2.29166900
H	-2.97117600	-1.91230500	3.31294300
C	0.75361900	6.64690100	-0.78544500
H	-0.33815800	6.79545500	-0.74425500
C	-5.66479600	-0.79157700	-3.63004300
H	-5.25305600	-1.68480900	-4.12798400
C	-1.45245200	-6.29441700	1.83614000
H	-1.15841600	-6.09215200	2.87893600
C	-1.55325300	1.54996000	-3.90964400
N	5.15145200	-1.02181900	2.61093900

C	-4.73116700	-1.78139200	-1.73821100
H	-4.50414400	-2.66958100	-2.34831500
C	-4.30960400	-1.84868300	-0.32542500
C	-4.41222000	1.45039500	4.79774500
H	-4.81643000	2.26842500	4.17916300
C	0.44112300	2.85708600	-3.47166000
C	-0.95361500	2.71566700	-3.44769200
H	-1.55119500	3.53678800	-3.05879000
C	-4.05996200	-0.79304300	1.84194000
C	1.14947100	6.29537500	-2.22145500
H	2.23692600	6.11477700	-2.23745600
C	5.47350000	0.95370200	4.02125100
H	6.02477700	1.39195300	3.17264500
N	3.88031100	-2.43303300	-3.52773200
C	5.58620900	-0.56992800	3.91503500
H	5.01109500	-1.02720600	4.73669800
N	0.73627000	-5.48446300	1.15624900
C	3.64831100	-2.39237400	-1.13368600
C	4.18681700	-1.84251800	2.56529400
H	3.69295400	-2.21525400	3.47608900
C	3.42914600	-1.45754000	-5.69864500
H	2.76962900	-2.31171800	-5.92317800
C	4.15583100	-1.90197000	0.06355300
H	4.94178600	-1.15098900	0.07460600
C	2.14712400	-3.84128700	0.09977600

C	4.51866900	-1.91714900	-4.72610300
H	5.15745900	-1.04834400	-4.49653700
C	2.66282700	-3.33682300	1.30223000
H	2.27045600	-3.72361000	2.23946500
C	2.63387100	-3.35957200	-1.10925500
H	2.24910500	-3.73155000	-2.05518200
C	3.65971200	-2.36836400	1.29012100
C	-0.19357900	-6.59326500	1.01831600
H	-0.49788500	-6.73158500	-0.03227300
C	1.13118400	-4.91293500	0.09535200
H	0.76424800	-5.21279300	-0.89855800
C	4.20109600	-1.91365500	-2.41659800
H	4.92901000	-1.09040500	-2.34557000
C	6.07500100	1.44362500	5.33681400
H	5.53461600	1.00773600	6.18250700
H	7.13066900	1.16414000	5.40858700
H	5.99675500	2.53148500	5.40892500
C	0.48842300	-7.86059200	1.53059500
H	0.78581800	-7.73183100	2.57564200
H	-0.18627500	-8.71893700	1.45719300
H	1.38706500	-8.07241100	0.94554200
C	-7.18865600	-0.79521800	-3.73780600
H	-7.60349800	0.08449400	-3.23661400
H	-7.50208400	-0.78565600	-4.78615900
H	-7.60258700	-1.68631000	-3.25875200

C	0.79942300	7.43920500	-3.17087900
H	-0.27862600	7.62561900	-3.15379300
H	1.32167800	8.35571600	-2.87989700
H	1.08431200	7.18635700	-4.19551200
C	-5.46864100	0.98822300	5.79998700
H	-5.07381100	0.17337300	6.41423900
H	-5.76504700	1.81322100	6.45487000
H	-6.35644700	0.62136700	5.27824000
H	-3.49846500	2.77630900	6.20620000
H	6.64172100	-0.84428900	4.03420500
H	-5.42589900	0.44625700	-5.35971400
H	-2.09022500	-7.18669000	1.82116200
H	1.24102700	7.59423400	-0.52215300
H	3.91366600	-1.13768400	-6.62943200
C	5.36006900	-3.02867800	-5.34972600
H	5.83339700	-2.68290700	-6.27376500
H	6.14213300	-3.34922400	-4.65658700
H	4.73072500	-3.89446300	-5.57647800
C	-1.30665600	-0.55224800	-1.02083400
C	-1.68183400	1.35702400	-0.12863300
C	0.71979200	-0.66705800	0.21203700
C	0.07931600	-0.57088300	-1.03101900
C	-0.97676900	1.04309700	1.02014700
H	1.79957000	-0.58730800	0.27616600
H	0.61638900	-0.11866700	-1.86108700

H	-1.52973900	0.65725100	1.89268500
O	0.30139400	0.91998900	1.00822700
H	0.27655400	-1.30582600	0.97369800
H	-1.82390900	-1.11730500	-0.25230800
H	-1.17367500	1.89292400	-0.92283200
H	-2.76519100	1.45043500	-0.08803000
H	-1.88707800	-0.38867900	-1.92766400

Product

0 1

N	2.69355600	-5.11374000	-1.00424900
N	-3.53757800	-1.05738100	-4.24220900
N	-2.00401400	-4.16082400	3.28620500
N	-3.87422300	4.14368100	-0.51465100
N	-3.18329100	4.20899600	2.30027400
N	-3.25378800	-3.64040400	-2.96402400
N	1.06964500	3.37660600	-4.51354100
C	-0.24096000	-4.37286500	1.02110100
H	0.18482700	-4.52858100	2.00942400
C	-4.40762400	4.75944100	1.76024800
H	-5.22882300	4.02492800	1.73397500
N	2.60768500	2.51956800	4.56245700
C	-2.00300500	-3.78515700	-2.81919100
H	-1.31224100	-3.80200200	-3.67692700
N	-2.26722000	-1.62823400	4.74957300
C	-1.38922400	-3.96598800	-1.48910100

C	-2.41912200	-3.83896000	2.13299400
H	-3.42181900	-3.41936600	1.96150800
C	-1.59235700	-4.01790100	0.92423400
C	1.95731200	-4.88599300	0.00223300
H	2.33891100	-4.96349400	1.03234000
C	1.62753000	3.01818000	3.93172400
H	1.67921100	4.01432000	3.46484500
C	-2.16016200	-3.81690400	-0.32736800
H	-3.20361900	-3.52949200	-0.42495000
C	-0.04438500	-4.30289900	-1.38328900
H	0.57662100	-4.42023100	-2.26779300
C	0.53539200	-4.51254900	-0.12392100
C	-1.18187700	3.14773300	-2.73844800
H	-0.55011000	4.02779100	-2.64677700
C	-3.68909000	-0.26390600	-3.26530200
H	-4.51892800	-0.36947000	-2.54840000
C	-0.81863300	2.15617600	-3.66056400
C	-0.75321400	2.95555700	3.23387400
H	-0.68288700	3.98613900	2.89475800
C	0.38352100	2.31063000	-4.50205500
H	0.63498900	1.44821000	-5.13909900
C	0.34279300	2.30674100	3.78986300
C	-3.13485500	1.87131600	-2.12722000
H	-4.03692200	1.78629400	-1.52556200
C	-3.16532600	2.96432900	2.54350600

H	-4.03964700	2.31888700	2.36761300
C	0.21397300	0.97885400	4.22712400
H	1.08098400	0.49297000	4.66763100
C	-3.76466100	-3.46475600	-4.30451700
H	-2.97412900	-3.49707700	-5.07217700
C	-1.14240200	-1.05233900	4.65153500
H	-0.21271200	-1.52534600	5.00612900
C	-1.62175400	1.03286900	-3.80954300
H	-1.37393200	0.25874700	-4.53134700
C	-2.90358200	-3.94266700	4.39800200
H	-3.89336500	-3.57660800	4.07914100
C	-4.17315200	5.28026500	0.33857600
H	-3.32801700	5.98749100	0.37276700
C	2.22305200	3.41807400	-5.38574500
H	2.33392700	2.50125300	-5.98741600
C	-1.98091100	2.28775300	3.10545700
N	4.81201600	-3.14497400	-0.81295300
C	-2.73328100	4.10375100	-1.06473100
H	-1.98612200	4.89916200	-0.91762500
C	-2.33538700	3.01342000	-1.97687900
C	-4.50465600	-2.12791100	-4.40830900
H	-5.26788400	-2.10408600	-3.61320600
C	-1.00059900	0.31585300	4.11624100
C	-2.09613400	0.97559500	3.54238300
H	-3.03575100	0.43927500	3.44999800

C	-2.79015400	0.88833700	-3.04805600
C	-2.30377900	-2.93585000	5.38171400
H	-1.29026300	-3.28201600	5.64415100
C	4.07636800	-5.48187100	-0.75757000
H	4.30469400	-5.47796600	0.32111500
N	4.70291400	2.29525400	2.60178600
C	4.97532400	-4.44271000	-1.43337600
H	4.74905900	-4.42791100	-2.51211200
N	3.68736900	2.46254800	-3.71682800
C	4.32745200	0.79593000	0.76032800
C	4.40666800	-2.19508800	-1.54825900
H	4.18842000	-2.33996400	-2.61803200
C	3.82919300	3.29049100	4.62605100
H	3.74949400	4.25951100	4.10654500
C	4.40766700	-0.51732200	0.31351800
H	4.62482500	-1.33064100	1.00133700
C	3.89753000	1.53751900	-1.50532000
C	4.98335200	2.49090500	4.01316900
H	5.04385400	1.52707800	4.54514600
C	3.98169500	0.20961900	-1.94649500
H	3.86294400	0.00648300	-3.00785000
C	4.06515900	1.82306000	-0.15639600
H	4.01807400	2.84543100	0.20995200
C	4.24214700	-0.81644700	-1.04644700
C	3.49880900	3.62660000	-4.56426700

H	3.36721900	4.54127500	-3.96288800
C	3.68219100	2.64083300	-2.46163700
H	3.54246800	3.63803600	-2.01541400
C	4.57735200	1.10543900	2.18257300
H	4.67641100	0.23684600	2.85202900
C	4.33048200	-6.87120800	-1.33696600
H	4.10427100	-6.87931200	-2.40739900
H	5.37472300	-7.16507400	-1.19328200
H	3.69040000	-7.61069300	-0.84890700
C	4.71172900	3.77574500	-5.47880500
H	4.84378800	2.87046200	-6.07878000
H	4.58636700	4.63075400	-6.15011800
H	5.61877300	3.92709300	-4.88799900
C	-5.41938600	5.98343200	-0.19243100
H	-6.26161400	5.28567500	-0.22018800
H	-5.68373800	6.83279800	0.44476900
H	-5.24814700	6.35007100	-1.20785900
C	-3.16036100	-2.83966400	6.64265600
H	-4.16989300	-2.50434400	6.38658400
H	-3.22542100	-3.81213600	7.14014200
H	-2.73023600	-2.11816900	7.34219600
C	-5.16991200	-1.98114800	-5.77434400
H	-4.41498300	-2.01045800	-6.56568400
H	-5.88989400	-2.78805900	-5.94122300
H	-5.69617300	-1.02554200	-5.84216600

H	-4.48089600	-4.27048200	-4.50678600
H	6.01906000	-4.75321300	-1.30149700
H	-4.71356000	5.60294800	2.39064700
H	2.10815800	4.27030200	-6.06642100
H	-3.03723800	-4.89715100	4.92180200
H	4.06434800	3.47884700	5.68079600
C	6.30143500	3.24723000	4.15561400
H	6.52620500	3.43635000	5.20974400
H	7.12218600	2.66924300	3.72307500
H	6.24454100	4.20419800	3.62839400
C	0.46419200	0.74190900	-0.71325100
C	-4.00871600	-0.69408700	0.94065100
C	-0.41673200	-0.98520800	0.84602000
C	0.00310700	0.41024700	0.48978800
C	-2.73313800	-0.68001500	0.55361000
H	0.21802500	-1.39608000	1.63825500
H	-0.07624000	1.15081800	1.28474400
H	-2.42293300	-0.37998600	-0.44842500
O	-1.73459900	-1.03926700	1.39286900
H	-0.34626900	-1.64780900	-0.02827600
H	0.54105500	0.00827000	-1.51380900
H	-4.28128800	-0.99643800	1.94740800
H	-4.79084700	-0.40726400	0.24897400
H	0.79165700	1.75044400	-0.94728500

8. [2+4] cycloaddition reaction

Without CC2 cage

Reactant

0 1

C	-2.60255000	-0.25584400	0.24244100
C	-2.05360900	0.61732200	-0.59438900
C	1.13834400	1.43728900	0.54746900
C	1.81960100	0.56522000	-0.19875700
C	1.40072900	-0.82242300	-0.45730900
C	0.69020800	-1.57202200	0.38838600
H	-3.50867400	-0.02877500	0.79689400
H	-2.48835400	1.59911600	-0.76095400
H	1.51042300	2.44219100	0.71933800
H	2.74648300	0.88397600	-0.67345100
H	1.71842200	-1.25336500	-1.40589200
H	0.39690500	-1.19861000	1.36600700
H	0.39352400	-2.58503500	0.13537700
H	0.18169000	1.17710900	0.99320100
H	-1.14233200	0.38378400	-1.13799700
H	-2.16442800	-1.23764700	0.40042900

TS

0 1

C	1.56806200	0.68819300	-0.23071000
C	1.56806600	-0.68819100	-0.23070300
C	-0.45053300	-1.42336600	0.49867900
C	-1.30999600	-0.70489300	-0.29634500

C	-1.30999600	0.70489400	-0.29634300
C	-0.45053200	1.42336400	0.49868300
H	2.05662200	1.23378900	0.57069000
H	2.05662700	-1.23377700	0.57070400
H	-0.38456200	-2.50307200	0.39836600
H	-1.84187000	-1.21878400	-1.09368200
H	-1.84187000	1.21878700	-1.09367900
H	-0.11884000	1.03233800	1.45374100
H	-0.38456100	2.50307100	0.39837300
H	-0.11884100	-1.03234300	1.45373900
H	1.44343800	-1.23293400	-1.15890700
H	1.44342900	1.23292500	-1.15891800

Product

0 1

C	-1.21009500	-0.77388500	-0.20430900
C	-1.20964300	0.77459100	-0.20431000
C	0.09050400	1.37966400	0.36518300
C	1.29512000	0.66612100	-0.18990100
C	1.29473200	-0.66687500	-0.18990100
C	0.08970000	-1.37971700	0.36518200
H	-2.06413300	-1.15838900	0.36286000
H	-2.06345700	1.15959200	0.36285900
H	0.12624800	2.45293300	0.15772800
H	2.13493300	1.23761200	-0.57654000
H	2.13421200	-1.23885500	-0.57654100

H	0.08159100	-1.27416900	1.46012300
H	0.12481800	-2.45300700	0.15772600
H	0.08233400	1.27411800	1.46012500
H	-1.32889800	1.13649200	-1.23000000
H	-1.32956200	-1.13571700	-1.22999900

With CC2 cage

Reactant

0 1

N	2.83138700	2.03968100	4.37800200
N	-4.73163200	-0.33908000	3.23235600
N	-0.06590500	5.75907400	0.05271400
N	-4.71829900	-1.68146600	-3.04065300
N	-3.04141700	-0.30467200	-4.93107000
N	-3.59209300	2.18088400	4.04444900
N	-1.42478500	-5.35796200	1.06876600
C	0.73510700	3.81022900	1.98976000
H	1.51474800	4.30931100	1.42064500
C	-4.45221600	-0.53793600	-5.15021100
H	-5.08264900	0.28180300	-4.76878700
N	3.34211700	-0.01229700	-4.58129500
C	-2.36913000	1.87815500	4.18543200
H	-2.03619600	1.11340300	4.90501000
N	-0.19340000	4.97923600	-2.72835000
C	-1.30089800	2.53971900	3.41262400
C	-0.94338300	5.02244200	0.59478600

H	-2.00525100	5.06727800	0.30602000
C	-0.60385500	4.06707800	1.66667600
C	2.47283500	2.72880500	3.37554200
H	3.20041600	3.23556200	2.72346600
C	2.12931100	-0.37593900	-4.52129700
H	1.80704400	-1.38517300	-4.82213800
C	-1.61477300	3.43113800	2.37691100
H	-2.66199300	3.61590200	2.15033900
C	0.03057200	2.29245600	3.72607300
H	0.29917400	1.61013100	4.52893000
C	1.05566500	2.93397900	3.01922200
C	-2.79170400	-3.28165600	-0.39843100
H	-2.31592500	-4.06732500	-0.98009100
C	-4.73494100	-0.30968700	1.96470000
H	-5.29503000	0.45309900	1.40120000
C	-2.57015100	-3.24422200	0.98455700
C	-0.27271400	0.18461000	-4.31068400
H	-0.53098900	-0.77860400	-4.74370700
C	-1.77702800	-4.29150000	1.65772900
H	-1.55802700	-4.11266500	2.72203700
C	1.05323900	0.54139000	-4.09779400
C	-4.24774400	-1.36723400	-0.22151000
H	-4.90775800	-0.65453800	-0.70985500
C	-2.71429400	0.73381600	-4.28161700
H	-3.46444800	1.45121200	-3.91246700

C	1.35314100	1.79862400	-3.55392100
H	2.39719200	2.06336300	-3.40447800
C	-4.55553900	1.45677300	4.84346400
H	-4.08129100	0.75634800	5.55024700
C	0.67043800	4.05263400	-2.77568200
H	1.72436300	4.22036800	-2.50313100
C	-3.17856900	-2.25593400	1.74810700
H	-3.03058500	-2.21173000	2.82424900
C	-0.49044200	6.64911600	-1.00377400
H	-1.57698200	6.61067900	-1.18577500
C	-4.88125200	-1.84458100	-4.47378500
H	-4.23656200	-2.65125600	-4.86030000
C	-0.72273500	-6.35237700	1.85198600
H	-0.62521700	-6.06696200	2.91218100
C	-1.30686300	1.07931900	-4.00242200
N	4.52570000	-0.05258600	3.28776200
C	-3.87413500	-2.42503500	-2.45539800
H	-3.28688200	-3.18024900	-3.00101600
C	-3.62813100	-2.35057900	-1.00300600
C	-5.50444100	0.67086700	3.93375000
H	-5.96458000	1.38288800	3.22877000
C	0.33518500	2.69497900	-3.25108900
C	-0.99858400	2.32607200	-3.47276100
H	-1.78068400	3.04495700	-3.24163900
C	-4.02775100	-1.31605200	1.14930400

C	0.24334700	6.29674600	-2.30179400
H	1.32492200	6.30997400	-2.08859600
C	4.25474400	1.95854500	4.65573800
H	4.84096500	2.49411600	3.89032200
N	4.69114200	-1.89557700	-2.84256500
C	4.67508000	0.48827100	4.62178200
H	4.09149700	-0.06867000	5.37291000
N	1.45694900	-5.39572600	1.47561900
C	4.00846400	-1.83891200	-0.53961200
C	3.80261200	-1.08555500	3.15289400
H	3.29728600	-1.55942200	4.00955900
C	4.30947300	-0.98257300	-5.04584400
H	3.84664000	-1.93979800	-5.33660400
C	4.18150700	-1.21065000	0.68675300
H	4.76458000	-0.29788900	0.76617200
C	2.67876000	-3.55595600	0.53375200
C	5.35625400	-1.24964100	-3.95993700
H	5.78647300	-0.27916900	-3.66255600
C	2.85925200	-2.91375300	1.76697600
H	2.39894500	-3.34936600	2.65031600
C	3.25011800	-3.01585500	-0.61214000
H	3.12641900	-3.49589200	-1.57964100
C	3.60622400	-1.74497500	1.84784800
C	0.67705700	-6.60417700	1.28423700
H	0.57121800	-6.84880800	0.21434900

C	1.88673700	-4.79744300	0.44461400
H	1.69912200	-5.17572900	-0.57226800
C	4.64726400	-1.26866300	-1.74154400
H	5.09526200	-0.27132100	-1.61380700
C	4.52346700	2.56337200	6.03163500
H	3.94309700	2.03555600	6.79433600
H	5.58605800	2.49414500	6.28406400
H	4.22924100	3.61589900	6.04902900
C	1.35769800	-7.75672800	2.01774000
H	1.45560100	-7.51969300	3.08138200
H	0.77733100	-8.67832500	1.91209700
H	2.35910900	-7.92781500	1.61435000
C	-6.34373600	-2.15610300	-4.77843500
H	-6.98549500	-1.35536200	-4.39901100
H	-6.50144400	-2.25590500	-5.85670100
H	-6.64358300	-3.08946200	-4.29505600
C	-0.08395400	7.30403500	-3.40075400
H	-1.15823300	7.29881200	-3.60790200
H	0.21440200	8.31317300	-3.10069000
H	0.44101100	7.04582900	-4.32406400
C	-6.58977600	-0.02074000	4.75511400
H	-6.13733400	-0.73059300	5.45397400
H	-7.17155100	0.71267900	5.32182700
H	-7.26897000	-0.57378400	4.10117200
H	-5.14638600	2.18307600	5.41448100

H	5.73642700	0.42882600	4.89364900
H	-4.62134400	-0.63573500	-6.22931400
H	-1.29105000	-7.28888400	1.79512500
H	-0.21895400	7.67371200	-0.72198400
H	4.82030700	-0.56246800	-5.92083400
C	6.45587400	-2.16945500	-4.48290500
H	6.96199400	-1.71776600	-5.34154400
H	7.19717700	-2.35969800	-3.70253500
H	6.02945300	-3.12981700	-4.78773300
C	2.57831500	1.67920100	0.16360900
C	3.70501600	2.30821600	-0.15878700
C	0.05217200	-0.65016100	1.93950000
C	0.31112900	-0.74584300	0.63291900
C	-0.64153100	-0.37350900	-0.42561800
C	-1.51511200	0.63068100	-0.32003400
H	2.49480600	1.10319100	1.08351000
H	4.57788400	2.26877800	0.48993900
H	0.80427600	-0.89197100	2.68611600
H	1.28363900	-1.11517500	0.30673400
H	-0.60262600	-0.94915700	-1.35015700
H	-1.54425600	1.26236900	0.56580400
H	-2.21911600	0.85107100	-1.11730100
H	-0.92348100	-0.32710200	2.29782300
H	3.80386200	2.88583100	-1.07433200
H	1.69052400	1.70629900	-0.46595800

TS

0 1

N	2.64053400	-3.63792900	3.81369900
N	-5.10469200	-1.22259100	2.56491600
N	2.14387200	2.73681800	4.59211700
N	-4.26476200	3.03467300	-2.19492000
N	-1.77787600	4.65588600	-2.55426600
N	-3.13298100	-0.94855700	4.73359300
N	-3.49597700	-3.24312100	-3.32229200
C	1.84520100	-0.11017300	4.25841900
H	2.84905600	0.30464200	4.21330100
C	-3.13553400	5.16378100	-2.58966800
H	-3.48347700	5.50435000	-1.60080900
N	4.21257400	2.39314500	-3.30445400
C	-2.12657500	-1.66578400	4.44996200
H	-2.21339200	-2.75099400	4.28319400
N	2.30991000	4.73493100	2.38853200
C	-0.75884100	-1.11554400	4.35820800
C	0.98622700	2.22156200	4.56462000
H	0.07304900	2.83262300	4.64109600
C	0.76811400	0.76594000	4.44724500
C	2.77193000	-2.37957200	3.89498000
H	3.75546700	-1.89029200	3.81760500
C	2.99724100	2.74698300	-3.37408500
H	2.40388600	2.61692900	-4.29304100

C	-0.52403000	0.25890700	4.50604000
H	-1.37592900	0.91684900	4.66074400
C	0.31544000	-1.97355800	4.15315200
H	0.16329200	-3.04310700	4.03254000
C	1.62538100	-1.47440600	4.10799600
C	-4.00481800	-0.58983800	-2.33910600
H	-3.70072100	-0.69691100	-3.37696900
C	-5.06002400	-0.22872400	1.77889200
H	-5.26636400	0.79686200	2.12390600
C	-4.03662200	-1.73404300	-1.52971900
C	0.99396300	3.84269900	-2.41853900
H	0.48796100	3.73562600	-3.37522100
C	-3.71074100	-3.06154600	-2.08631500
H	-3.68623600	-3.89068600	-1.36180000
C	2.28981800	3.37595100	-2.24240400
C	-4.70332000	0.75909100	-0.46736900
H	-4.94214700	1.74241900	-0.06908100
C	-1.09851800	4.87989500	-1.50614700
H	-1.52819900	5.40023900	-0.63527600
C	2.90698200	3.50417700	-0.98933000
H	3.91371900	3.11339400	-0.86369000
C	-4.41355400	-1.61352000	4.85739800
H	-4.35544100	-2.69018100	4.62786900
C	2.88150500	4.20059000	1.39110600
H	3.89410500	3.77287700	1.45834600

C	-4.38295500	-1.61516600	-0.18981600
H	-4.39821100	-2.48323600	0.46429300
C	2.23289200	4.17278400	4.75408100
H	1.24424100	4.65904700	4.78942800
C	-4.12196200	4.13262600	-3.13831400
H	-3.73543600	3.77519700	-4.10696100
C	-3.21088400	-4.59096200	-3.76354600
H	-3.22164900	-5.32168400	-2.93817500
C	0.30564200	4.45122700	-1.35906900
N	3.62083000	-4.42894700	1.18320800
C	-4.16601100	1.85609500	-2.65436100
H	-3.93142400	1.66382900	-3.71315500
C	-4.32191000	0.65468400	-1.81212300
C	-5.46628900	-0.97391800	3.94985700
H	-5.49555100	0.10653600	4.16742400
C	2.23854200	4.11257800	0.06522800
C	0.94207400	4.60936200	-0.13533900
H	0.43587500	5.08460300	0.70177400
C	-4.72247400	-0.36531700	0.34851200
C	3.05579100	4.81191000	3.63348300
H	4.01176400	4.26753000	3.56109400
C	3.84296900	-4.43104300	3.62587200
H	4.72896300	-3.78809600	3.49440900
N	4.24278700	-0.47672300	-3.85655800
C	3.67843600	-5.27305900	2.35778900

H	2.78407400	-5.90799300	2.46775700
N	-0.80431200	-4.43710600	-3.47771300
C	3.23071700	-2.05282700	-2.34313100
C	2.58657400	-4.51162700	0.45472300
H	1.75773400	-5.19577800	0.69488000
C	4.80397200	1.79946100	-4.48396400
H	4.09268100	1.72056500	-5.32244700
C	3.38050400	-2.77094500	-1.16191600
H	4.24124300	-2.61465300	-0.51658600
C	1.15784900	-3.22640500	-2.79015400
C	5.35357000	0.40643400	-4.16946200
H	6.04252000	0.49923500	-3.31372800
C	1.31805500	-3.94423700	-1.59697200
H	0.56471200	-4.68095200	-1.32993200
C	2.11184400	-2.28461500	-3.15586900
H	2.01380600	-1.71851000	-4.07873000
C	2.42284000	-3.72277200	-0.78271300
C	-1.85068500	-4.65592700	-4.46198300
H	-1.83410200	-3.87822300	-5.24322300
C	0.01085600	-3.48826000	-3.68300300
H	-0.07266900	-2.82492600	-4.55773200
C	4.26857100	-1.08121700	-2.74246300
H	5.08475400	-0.93330700	-2.01811100
C	4.03253500	-5.33123900	4.84494200
H	3.15483600	-5.97026700	4.98063600

H	4.91661000	-5.96470500	4.72418200
H	4.15673400	-4.72842000	5.74827000
C	-1.63834700	-6.03017800	-5.09436900
H	-1.66202000	-6.80667200	-4.32383000
H	-2.41804900	-6.24003100	-5.83288800
H	-0.66596800	-6.07491200	-5.59167700
C	-5.49537400	4.77398900	-3.33830900
H	-5.88941300	5.12588100	-2.37992500
H	-5.43148000	5.62206900	-4.02694400
H	-6.19947300	4.04484500	-3.74754200
C	3.32203400	6.28451700	3.94319400
H	2.37620800	6.82861600	4.02560900
H	3.87318500	6.38849900	4.88277500
H	3.90846200	6.74239600	3.14235000
C	-6.83941600	-1.59071300	4.21244900
H	-6.81896100	-2.66206400	3.99090700
H	-7.13337400	-1.45086300	5.25719100
H	-7.59416800	-1.12596600	3.57268000
H	-4.74752700	-1.50015900	5.89664100
H	4.55653900	-5.92397800	2.26373800
H	-3.14468000	6.02609400	-3.26953800
H	-3.98117500	-4.87741200	-4.49045700
H	2.74730300	4.36782300	5.70376000
H	5.64217800	2.43356500	-4.79874600
C	6.10004600	-0.16003100	-5.37570000

H	6.93134600	0.49354400	-5.65716700
H	6.49868200	-1.15166100	-5.14634600
H	5.42037300	-0.25545300	-6.22778400
C	-0.11523000	1.72959100	1.28173000
C	0.92897100	0.86982500	1.04436000
C	0.34427100	-0.54270900	-0.62897200
C	0.07995300	0.34193900	-1.64436600
C	-0.92599900	1.32538500	-1.52121800
C	-1.67048600	1.45400500	-0.37591300
H	-0.84618600	1.49081600	2.04911300
H	1.06407700	-0.01245200	1.66213100
H	1.19849300	-1.21015800	-0.68554900
H	0.79890700	0.43287300	-2.45877800
H	-0.98479800	2.11588800	-2.26632700
H	-1.90380200	0.59195800	0.23863900
H	-2.35574000	2.29372000	-0.27508000
H	-0.44164200	-0.85609200	0.05019600
H	1.80909500	1.21725100	0.51236900
H	-0.05013700	2.77158300	1.00030700

Product

0 1

N	1.24692200	3.75834200	-4.29732900
N	-5.74240600	0.85695100	-1.10488100
N	1.00888900	-2.61506600	-4.95612400
N	-3.22984300	-3.32962200	3.11794400

N	-0.64352800	-4.79023100	2.82651000
N	-4.37579800	0.66937900	-3.68087800
N	-2.60640800	2.97492700	4.10151900
C	0.62207500	0.17364800	-4.39189700
H	1.63311100	-0.16972200	-4.59528100
C	-1.91122200	-5.38337900	3.20273200
H	-2.47990200	-5.75198800	2.33352400
N	5.16585200	-2.10542000	1.98572600
C	-3.38206100	1.45658000	-3.65714000
H	-3.50085300	2.53816000	-3.48665800
N	1.96848500	-4.60198300	-3.01813000
C	-1.99722900	1.00020900	-3.89163300
C	-0.12336600	-2.20831200	-4.55598900
H	-0.97017400	-2.89508800	-4.40058600
C	-0.40438900	-0.77798100	-4.31620500
C	1.44137800	2.50646400	-4.34402200
H	2.44519100	2.07897000	-4.49289500
C	4.03644500	-2.53344000	2.37146100
H	3.70559500	-2.44340000	3.41810200
C	-1.70614000	-0.36094000	-4.06514500
H	-2.52303500	-1.07653600	-4.01146400
C	-0.97197500	1.93530000	-3.97668500
H	-1.17123300	2.99719900	-3.85590300
C	0.34545700	1.52493600	-4.22768800
C	-3.11836000	0.30813500	3.19175700

H	-2.50904500	0.43576600	4.08250900
C	-5.37834900	-0.12892700	-0.39588200
H	-5.57339300	-1.16925600	-0.70041200
C	-3.51902900	1.45032000	2.48575800
C	1.92087900	-3.75576600	1.96738200
H	1.68676400	-3.68940100	3.02706800
C	-3.15730200	2.79553200	2.97397100
H	-3.41635200	3.63462000	2.30931000
C	3.08897700	-3.20083500	1.45841100
C	-4.26389600	-1.08887700	1.59682500
H	-4.53922700	-2.08671600	1.26336000
C	-0.26616000	-4.93494100	1.62406300
H	-0.88644600	-5.45966100	0.88000200
C	3.34912300	-3.27646000	0.08343800
H	4.26021300	-2.82283800	-0.29830500
C	-5.68914900	1.25101800	-3.49395800
H	-5.64718300	2.33510800	-3.29840300
C	2.72906100	-3.98019900	-2.21689700
H	3.63454200	-3.45494000	-2.55867800
C	-4.27331400	1.30916400	1.32713200
H	-4.57931600	2.17629800	0.74738000
C	1.16159100	-4.02892100	-5.22579400
H	0.24910700	-4.60584800	-5.00314500
C	-2.78068200	-4.40353100	3.99061000
H	-2.17454500	-4.01012400	4.82342000

C	-2.31465000	4.33318800	4.50310300
H	-2.58714700	5.07422800	3.73375600
C	1.01317100	-4.40198000	1.11795700
N	2.90075000	4.61893000	-2.05775800
C	-3.02396200	-2.13993000	3.50759400
H	-2.48161900	-1.92091300	4.44100000
C	-3.48524000	-0.95778200	2.75449700
C	-6.44074000	0.57266000	-2.34602500
H	-6.46819500	-0.51065900	-2.54950700
C	2.45679900	-3.91787400	-0.76743800
C	1.29356400	-4.49276500	-0.23960200
H	0.60309500	-4.98381100	-0.92112800
C	-4.64579700	0.03570300	0.87420900
C	2.32551200	-4.61804300	-4.42489300
H	3.21846900	-4.00120900	-4.61960400
C	2.40323700	4.62332700	-4.45665600
H	3.32618900	4.03513200	-4.59014000
N	5.17733200	0.75658900	2.56725100
C	2.56237600	5.46165000	-3.18501700
H	1.63649400	6.03712800	-3.02195700
N	-0.05983000	4.33079500	3.61390000
C	3.66712000	2.22387100	1.40121400
C	2.09958400	4.61257300	-1.07533200
H	1.18382800	5.22416000	-1.06714900
C	6.01767000	-1.48125200	2.97479800

H	5.55516800	-1.44861700	3.97496100
C	3.43301900	2.93982500	0.23295500
H	4.09323000	2.84273500	-0.62522900
C	1.71720600	3.24531500	2.41484700
C	6.38165800	-0.05578100	2.55196000
H	6.81760700	-0.10401500	1.54040000
C	1.49155300	3.96303100	1.23200100
H	0.64315300	4.64137700	1.19181700
C	2.79953300	2.37769400	2.49301000
H	3.00277100	1.81413000	3.40029400
C	2.34136300	3.81596900	0.14347100
C	-0.82892100	4.48654800	4.83589600
H	-0.56621200	3.71239200	5.57562600
C	0.83809200	3.43635700	3.58615500
H	1.03091300	2.77362300	4.44430500
C	4.84304900	1.33510200	1.49003000
H	5.42691600	1.22425200	0.56300200
C	2.18150900	5.52338000	-5.66969500
H	1.26938200	6.11325800	-5.53872500
H	3.02729000	6.20423100	-5.80585000
H	2.06966800	4.92178700	-6.57541500
C	-0.54765200	5.87238500	5.41316100
H	-0.80943700	6.64416600	4.68311000
H	-1.12923900	6.03588300	6.32555000
H	0.51361100	5.97763000	5.65288200

C	-4.01427700	-5.11673400	4.54374200
H	-4.62049700	-5.51145700	3.72268100
H	-3.72273700	-5.94424000	5.19774700
H	-4.63155000	-4.42014400	5.11691900
C	2.58980600	-6.06189800	-4.84670400
H	1.70365200	-6.67622800	-4.66081000
H	2.84050500	-6.11443700	-5.91056200
H	3.41991600	-6.48077300	-4.27200700
C	-7.86441600	1.11679500	-2.24618300
H	-7.84260000	2.19076100	-2.03799800
H	-8.40994700	0.94825100	-3.17973500
H	-8.40446700	0.62415700	-1.43344600
H	-6.26090100	1.08894800	-4.41656300
H	3.38729400	6.16752300	-3.34148100
H	-1.69479000	-6.23771000	3.85754100
H	-2.89150200	4.54809300	5.41145800
H	1.39051900	-4.14138700	-6.29309100
H	6.94493300	-2.06411200	3.04052400
C	7.39198200	0.55069300	3.52339200
H	8.30579600	-0.05044000	3.55544800
H	7.65395900	1.56663100	3.21667000
H	6.96430100	0.59880500	4.52932700
C	-1.48220400	-0.75017000	-0.50785900
C	-0.19303400	0.02556100	-0.77278200
C	0.30316800	0.66514200	0.52587200

C	0.26290500	-0.30626400	1.67676900
C	-0.39400500	-1.46762900	1.63433500
C	-1.19752300	-1.91482900	0.43954800
H	-2.21065000	-0.07138600	-0.04737900
H	-0.34601800	0.79249100	-1.53907500
H	1.32326800	1.04788500	0.39990100
H	0.80903800	-0.02758300	2.57835000
H	-0.36945900	-2.13936400	2.49255700
H	-2.12359200	-2.39524400	0.78064500
H	-0.64067000	-2.68930100	-0.10569600
H	-0.31638800	1.54165300	0.77351300
H	0.57207700	-0.66554400	-1.15416500
H	-1.92504300	-1.11425800	-1.44207400
